# The role of GLUT2 in glucose metabolism in multiple organs and tissues

**DOI:** 10.1007/s11033-023-08535-w

**Published:** 2023-06-26

**Authors:** Bo Sun, Hui Chen, Jisu Xue, Peiwu Li, Xu Fu

**Affiliations:** 1 Endorcrine and Metabolism Department, Lanzhou University Second Hospital, Lanzhou, 730000 China; 2 Department of Infantile Endocrine Genetic Metabolism, Gansu Maternal and child Health Care Hospital, Lanzhou, 730000 China; 3 EndEnorcrine and Metabolism Department, Shenzhen Bao ’an People’s Hospital (Group), Shenzhen, 518100 China; 4 Key Laboratory of Emergency Medicine, Lanzhou University Second Hospital, Lanzhou, 730000 China

**Keywords:** GLUT2, Glucose metabolism, Blood glucose regulation, Blood glucose homeostasis

## Abstract

The glucose transporter family has an important role in the initial stage of glucose metabolism; Glucose transporters 2 (GLUTs, encoded by the solute carrier family 2, SLC2A genes) is the major glucose transporter in β-cells of pancreatic islets and hepatocytes but is also expressed in the small intestine, kidneys, and central nervous system; GLUT2 has a relatively low affinity to glucose. Under physiological conditions, GLUT2 transports glucose into cells and allows the glucose concentration to reach balance on the bilateral sides of the cellular membrane; Variation of GLUT2 is associated with various endocrine and metabolic disorders; In this study, we discussed the role of GLUT2 in participating in glucose metabolism and regulation in multiple organs and tissues and its effects on maintaining glucose homeostasis.

**Supplementary Information:**

The online version contains supplementary material available at 10.1007/s11033-023-08535-w.

## Introduction

Glucose is the primary source of energy for the cells in the body. The metabolism, utilization, surveillance, and glucose regulation are critical for the proper functioning of the body. As a consequence, a whole set of glucose-sensing systems is developed consisting of cells or molecular mechanisms that directly respond to variations of physiological glucose concentrations. Glucose provides ATP in animal cells through aerobic and anaerobic metabolisms. Due to its hydrophilic feature, glucose requires glucose cotransporters (SGLTs) and glucose transporters (GLUTs) to pass the bilayer lipid membrane and enter the cells [1]. GLUT2 is a member of this GLUTs family with relatively high glucose transport activity, mainly expressed on β cells and other tissues with high glucose concentrations (such as the intestine, liver, kidney, and nervous system) [2] that have a critical role in response to blood glucose metabolism and regulation. In this study, we discussed the role of GLUT2 in participating in glucose metabolism and regulation in multiple organs and tissues and its effects on maintaining glucose homeostasis.

## Structures and functions of GLUT2

GLUT2 protein consists of 14 subunits and is encoded by the SLC2A2 gene, located at q26.2 of chromosome 3. This gene mainly encodes glycoproteins on membranes of cells of the liver, pancreatic cells, intestine, and renal epithelium. A previous study performed RNA-seq in samples of 27 different tissues from 95 subjects to investigate the tissue specificity of the SLC2A2 encoding gene and found that liver tissue had the highest specificity, followed by the duodenum and small intestine [3]. GLUT2 has only a relatively low affinity to glucose (Km ≈ 17 mmol/L), as well as a low affinity to fructose, mannose, and galactose [4], but a high affinity to glucosamine (Km ≈ 0.8 mmol/L) [5]. In the physiological state, GLUT2 transports glucose into cells and interacts with glucokinase (GCK) to act as a glucose sensor to rapidly regulate the glucose concentrations on both sides of the cell membrane to achieve balance, thus matching the environmental glucose concentration. In the fasting state, glucose-6-phosphatase in the cellular endoplasmic reticulum hydrolyzes glucose-6-phosphate to glucose and phosphates. Glucose is then transported to the cytoplasm and transported out of cells by GLUT2 [6]. GLUT2 mediates the passive transmembrane transport of glucose, during which it interacts with cells of multiple organs participating in glucose metabolism. The transport activity of GLUT2 can control the gene expression of relevant mechanisms in cells and regulate intracellular metabolism pathways. Therefore, GLUT2 can promote communication among different organs and maintain glucose homeostasis by participating in the abovementioned mechanisms, pathways, and signal generation.

**Fig. 1:**
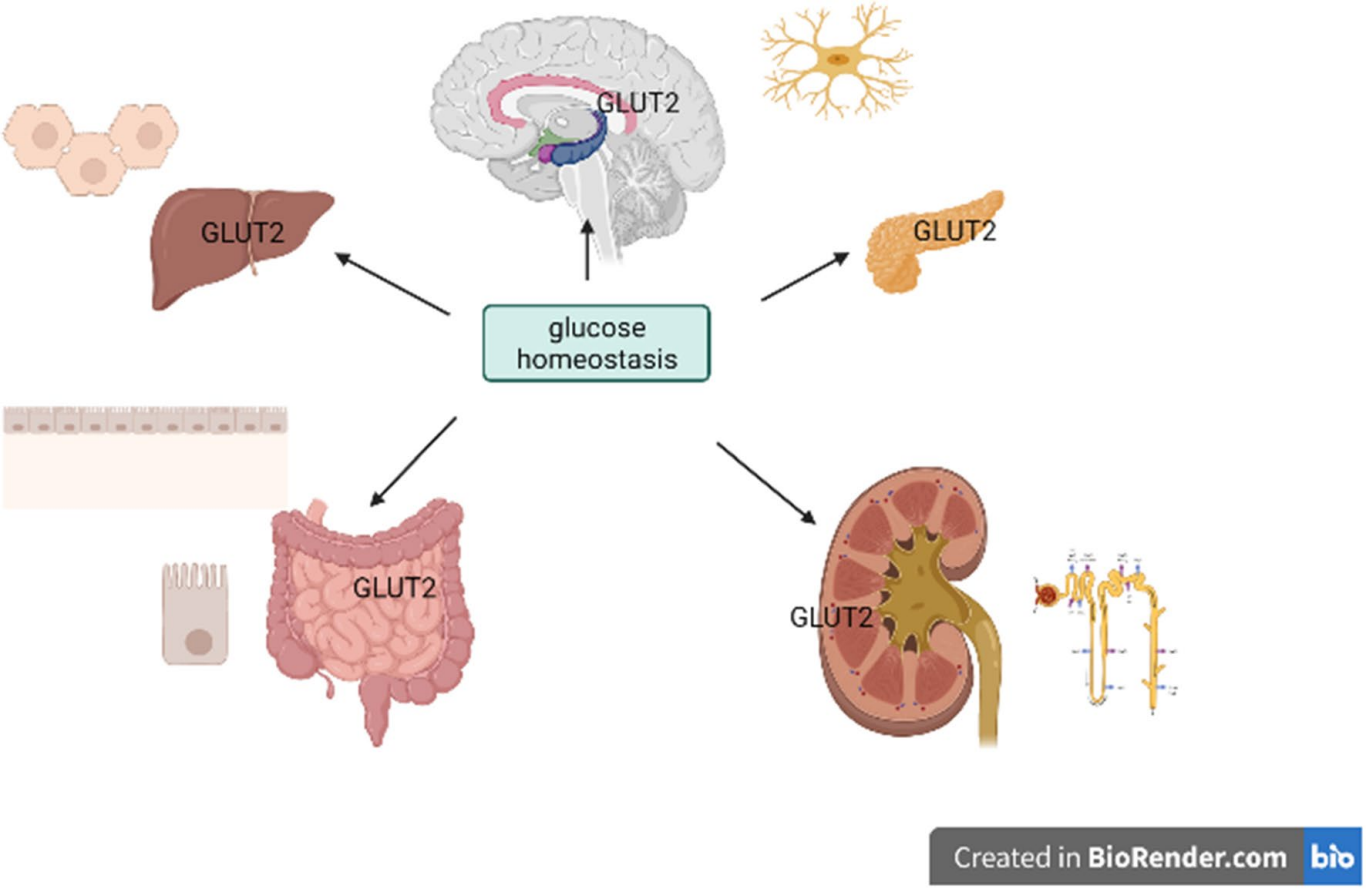
Glucose homeostasis

The level of GLUT2 expression and the importance of blood glucose regulation varies from tissue to tissue (Table 1). Besides the intestine, liver, kidney, and pancreatic β cells, GLUT2 is also expressed in the central nervous system (CNS), which senses glucagon induced by hypoglycemia to regulate blood glucose (Fig. 1).

**Table 1:** Organs, sites, and functions of GLUT2 expression

Organ	Site	Function
Intestine	Apical membrane of enterocytes	Glucose absorption (unessential) [9]
Kidney	Epithelial cells of the proximal convoluted tubule	Glucose absorption (essential) [10]
Liver	Hepatocyte membrane (mainly) Hepatocyte cytoplasm (small amount)	Glucose absorption (essential) Glucose output (not indispensable) [6, 11]
Pancreatic β cells	Pancreatic β cell membrane (apical membrane)	Glucose absorption, glucose-stimulated insulin secretion (essential) [7, 12]
Central nervous system	The nucleus of hypothalamic nuclei, gliocyte, astrocytes, and nucleus of the solitary tract	Participate in the secretion of glucagon following hypoglycemia [13] Satiety control [14] Excite vagus nerve [15]

## The role of GLUT2 in blood glucose utilization and metabolism

### The role of GLUT2 in pancreatic β cells

**Fig. 2:**
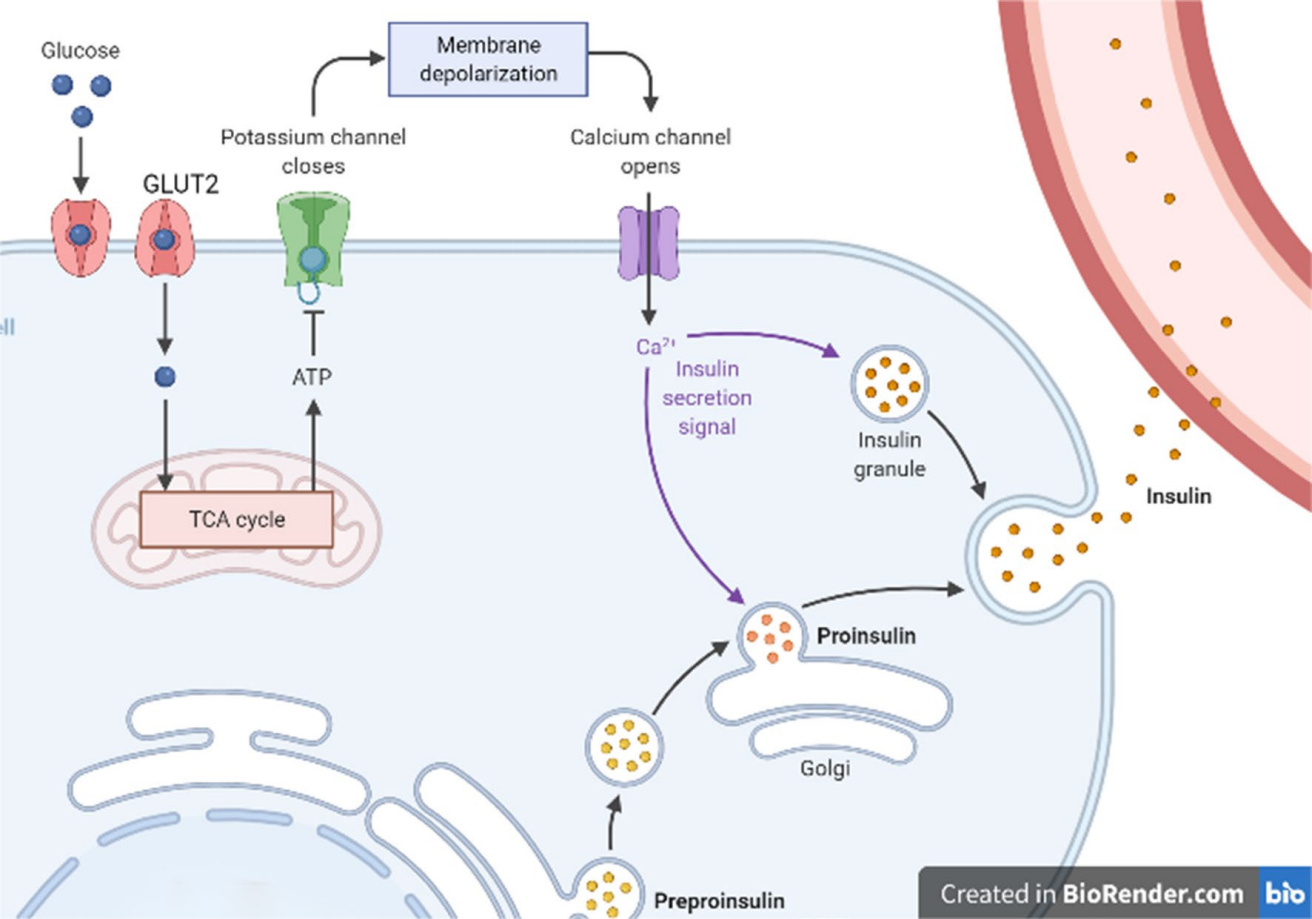
When blood glucose is elevated, β cells uptake glucose through GLUT2. Glucose is then metabolized to glucose 6-phosphate, followed by glycolysis or glucose oxidation to generate ATP through a series of reactions, which consequently increases the ratio of ATP/ADP in the cytoplasm, leading to the closure of the K^+^-ATP channel and opening of the Ca^2+^ channel. This allows large amounts of Ca^2+^ to enter the cells, finally promoting the migration of inclusion bodies with insulin to fuse with the cellular membrane, and secrete insulin particulates from cells through exocytosis [7, 8]. This pathway is the major trigger pathway for insulin secretion

Pancreatic β cells secrete insulin after glucose uptake, a process mediated by GLUT2 expressed on the cell surface. GLUT2 has an important role in pancreatic β cells through the canonical insulin secretion pathway, i.e., the K^+^-ATP-dependent pathway. When blood glucose is elevated, β cells uptake glucose through GLUT2. Glucose is then metabolized to glucose 6-phosphate, followed by glycolysis or glucose oxidation to generate ATP through a series of reactions, which consequently increases the ratio of ATP/ADP in the cytoplasm, leading to the closure of the K^+^-ATP channel and opening of the Ca^2+^ channel. This allows large amounts of Ca^2+^ to enter the cells, finally promoting the migration of inclusion bodies with insulin to fuse with the cellular membrane, and secrete insulin particulates from cells through exocytosis [7, 8] (Fig. 2). This pathway is the major trigger pathway for insulin secretion.

GLUT2 is a uniporter secreted in the first phase of insulin secretion. Gillam et al. showed that homozygous, but not heterozygous, mice deficient in GLUT2 are hyperglycaemic and relatively hypo-insulinemic and have elevated free fatty acids, plasma levels of glucagon, and beta-hydroxybutyrate. Also, GLUT2 knockout mice do not survive after initiating food intake following weaning [16]. Moreover, expressing GLUT1 or GULT2 in β cells of mice with GLUT2 gene knockout could restore the function of glucose-stimulated insulin secretion (GSIS), with GLUT1 inducing the second-phase insulin secretion [16, 17] and GLUT2 inducing the first stage insulin secretion. It was also reported that when the expression of GLUT2 in pancreatic β cells is no less than 20%, the GSIS could be maintained at a normal level.

The expression of GLUT2 in β cells is regulated by multiple factors, both at mRNA and protein levels [18]. GLUT-2 allows the fast balancing of blood glucose inside and outside cells during physiological conditions. There is an N-glycosylation site in the protein sequence of GLUT2 in vertebrates, which is a conserved amino acid that could interact with galectin-9 after glycosylation, hence anchoring GLUT2 at the cell surface and stabilizing the expression of GLUT2 on the surface of β cells. The glycosylation of this site is triggered by a special Golgi apparatus enzyme (i.e., N-acetylglucosamine transferase, OGT). When glucose-stimulated insulin secretion (GSIS) is impaired, the lack or reduction of OGT expression alters the structure of the N-glycosylation site, reduces GSIS, and, in turn, leads to the progression of diabetes [19, 20]. Previous studies have shown that a high-fat diet could reduce the expression of OGT to influence the glycosylation of GLUT2 and consequently influence the expression of GLUT2 [21]. These findings suggested that the Golgi apparatus enzyme OGT could affect the glycosylation site of GLUT2 protein and therefore influence the expression of GLUT2 protein.

The expression of GLUT2 in β cells is regulated by various factors at the genetic level. For instance, GLUT2 in pancreatic β cells is regulated by *PDX1*, which binds to the TATA box of GLUT2. Conditional knocking out the *PDX-1* gene in mice pancreatic β cells or functional inhibiting the transcription of the *PDX-1* gene could reduce the expression of GLUT2. On the contrary, overexpression of *PDX-1* could upregulate GLUT2 expression, indicating that *PDX1* regulates the transcription of GLUT2 [21]. Nuclear transcription factor Foxo1 inhibits the transcription of *PDX-1*, while the obese-related miRNA-27a that exists on pancreatic β cells could inhibit Foxo1 from upregulating GLUT2 expression and thus increase the secretion of insulin [22]. The expression of GLUT2 in pancreatic β cells could also be associated with the level of lncRNA-p3134 in circulation.[23]A previous study on Wiskott–Aldrich syndrome showed that endosomal WASH protein deficiency could influence the transport of GLUT2 protein in pancreatic β cells, consequently reducing the intracellular GLUT2 level and synthesis of protein, in turn, inducing the elevation of blood glucose [24].

Several studies have demonstrated that thyroid functions affect GLUT2 expression in β cells. Gholami et al. discovered that the expression of GLUT2 in pancreatic islets of rats with transient thyroid dysfunctions was lower than in young rats (12% and 10%, respectively) but higher than in aged rats (10.85 and 8.42 folds, respectively) [25]. Another study demonstrated that elevation of glucocorticoids could increase the degradation of GLUT2 [12].

These findings demonstrate that GLUT2 protein exists in pancreatic β cells and is a uniporter in the first stage of insulin secretion. Various factors at hormone, genetic, mRNA, and protein levels could regulate GLUT2.

### The role of GLUT2 in the liver

GLUT2 accounts for > 97% of all glucose transporters in hepatocytes. Yet, the expression of GLUT2 on cell membranes of liver tissues varies in healthy humans. For instance, GLUT2 is mainly expressed on membranes of hepatocytes close to hepatic sinus cavities, while a small amount is expressed in the cytoplasm. Furthermore, the intensity of expression decreases gradually from the central venous area to the portal area [11].

The expression of GLUT2 on hepatocytes is not only responsible for the bi-directional transportation of glucose from and into cells. In previous studies, the tamoxifen-dependent recombination system (LG2KO mice) was used to inactivate the GLUT2 on the surface of hepatocytes in mice, which then underwent regular feeding, fasting, and then regular feeding. The findings showed that the glucose uptake by the liver was inhibited, but the homeostasis of blood glucose was affected. The glucose uptake was also investigated, showing that the glucose uptake in mice’s tibialis anterior muscle and extensor digitorum longus was elevated. These findings suggest that although inactivation of GLUT2 inhibits the glucose uptake by the liver, the glucose uptake by other tissues remains elevated, consequently ensuring the system’s blood glucose balance [26]. Further assessment of glucose output in LG2KO mice showed that the inactivation of GLUT2 on hepatocytes did not influence the output of glucose [27]. The inactivation of GLUT2 on the surface of hepatocytes of LG2KO mice led to the aggregation of glucose in the cytoplasm and further accumulation of glucose-6-phosphate, which consequently upregulated the expression of nuclear carbohydrate responsive element binding protein (CHRBP). Consequently, CHRBP promoted the transformation of glucose to fatty acid and upregulated the expression of L-pyruvate kinase and lipid genes, which contradicts the model of fat motivation and energy saving in the fasting state [28]. Therefore, the inactivation or reduced expression of GLUT2 protein on the liver surface could be compensated by the expression of other transporters, and thus the influence on blood glucose balance is less substantial. However, in the state of fasting or starvation, the inactivation or reduced expression of GLUT2 on the liver surface could influence the fat and energy metabolism in the body.

Endocrine hormones and hepatitis viruses partially influence the expression of GLUT2 on the liver surface. Treating female mice with different doses of 17β-estradiol and progesterone significantly downregulated the expression of GLUT2 in the liver, indicating that certain doses of estrogen have some inhibitory effects on the expression of GLUT2 in the liver [29]. Previous animal studies have also shown that after inducing hyperthyroidism or hypothyroidism in mice with drugs, the contents of GLUT2 on hepatocyte membranes increased or decreased correspondingly, indicating that thyroxine has certain regulatory effect on GLUT2 on hepatocyte membrane [30].

The expression of GLUT2 in the liver is also influenced by the hepatitis C virus (HCV). In liver tissues of individuals with HCV infection, the GLUT2 expression is reduced. Previous studies [31] have shown that the expression of GLUT2 mRNA in HCV-infected cells is relatively low; the luciferase reporting assay showed that the activity of GLUT2 promoter was reduced in hepatocytes with HCV infection. After infection, the inhibitory effects of HCV on glucose uptake were mainly mediated by altering the expression of GLUT2; while after interferon treatment that suppresses the virus, the glucose uptake in the liver was restored, as well as the GLUT2 expression, GLUT2 mRNA expression, and GLUT2 promoter activity [31].

The normal level of GLUT2 is critical for glucose uptake in the liver but has no significant influence on systemic blood glucose homeostasis; however, the fat and energy metabolism in the body could be influenced in a fasting or starving state.

### The role of GLUT2 in the small intestine

The glucose metabolism in the small intestine relies on different transporters, such as GLUT1, GLUT5, and GLUT2. Glucose is transported through the apical membrane of enterocytes by GLUT1 and then through the basilar membrane into the blood by GLUT2, which regulates the glucose concentration in enterocytes and plasma. The glucose level in the canal of the small intestine is low before food intake; at that time, the level of GLUT2 in the apical membrane of enterocytes is also relatively low. In the fasting state, GLUT2 on the basilar membrane of enterocytes transports glucose from the blood to meet the energy demands of enterocytes [32]. After food intake, carbohydrates reaching the jejunum increase the glucose concentration in the intestinal canal. Then, the GLUT1 transports glucose from the intestinal canal to the apical membrane and activates protein kinase C (PKC) βII, through which the GLUT2 on the apical membrane is activated. More GLUT2 on the basilar membrane enters the apical membrane and participates in glucose transport, which transports glucose from the intestinal canal to blood vessels [33]. With the continuous absorption of glucose, the glucose concentration in the intestinal canal gradually decreases. The glucose transport signaling system starts to enter the resting state, and GLUT2 is gradually inactivated and translocated to the basilar membrane of enterocytes, finally returning to the state before food intake.

GLUT2 has an important role in glucose absorption in the small intestine, and 2 signaling pathways mainly regulate the process. The first pathway is the L-voltage-gated calcium channel, which allows GLUT2 to translocate the basilar membrane to the apical membrane. When the glucose concentration in the intestinal canal is increased, GLUT1 starts to transport glucose, and the apical membrane undergoes depolarization, allowing large amounts of Ca^2+^ to enter the enterocytes through this voltage-gated channel and trigger the reassembling of cytoskeletal structures, allowing GLUT2 to translocate to the apical membrane through the terminal web [34]. The second pathway is the sweet-taste receptor pathway. Sweet-taste receptors (T1R2 and T1R3) are taste receptor family 1 members (T1Rs). When the concentrations of glucose or fructose in the intestinal canal are elevated, T1R2 and T1R3 are activated [35]. After receiving the taste signals, PLCβ2 and PKCβII are translocated to the apical membrane of enterocytes, and then PLCβ2 is activated to produce diglyceride, which in turn activates PKCβII and induces the insertion of GLUT2 into the apical membrane.

During glucose absorption in the small intestine, GLUT2 is transiently inserted into the apical membrane of enterocytes to mediate facilitated diffusion. Food intake, Ca^2+^, and sweet-taste receptors have important regulatory effects on the insertion of GLUT2 into the apical membrane. Mice models of obesity and diabetes showed that hyperglycemia could alter the GLUT2-dependent transcription in enterocytes, influence the adhesion and integrity of tight junction between enterocytes, consequently changing the permeability of the intestinal barrier [36]. The continuous insertion of GLUT2 into the apical membrane could upregulate glucose absorption in the small intestine and therefore induce obesity, insulin resistance, and diabetes [37, 38]. Obesity could also induce T cell-mediated inflammation in the jejunum. Cytokines secreted by T cells can attenuate insulin signal transduction in intestinal cells that are associated with GLUT2 dislocation in the apical membrane of enterocytes [39]. Other studies demonstrated the influences of GLUT2 on body weight and food intake from another aspect. For instance, a study on body weight management by gastroenteroanastomosis and gastric band surgery showed that gastroenteroanastomasis could change the anatomical structures of the gastrointestinal canal and reduce the area of GLUT2 protein distribution, consequently influencing the food intake and energy metabolism of mice [40]. Another study showed that in GLUT2 gene knockout mice, the adaptation to intestinal GLUT2 absence was achieved through delaying the distribution of glucose from oral intake in tissues, reducing the length of microvilli, and modifying enteric microorganisms, which in turn induced malabsorption of glucose to restrict weight gain of mice.[41].

These findings demonstrate that GLUT2 could translocate from the apical membrane to the basilar membrane of enterocytes in the intestinal canal to regulate glucose absorption in a starving or full state and thus regulate energy metabolism to induce body weight changes.

### The role of GLUT2 in the kidney

Kidneys are capable of complete filtration of glucose in plasma and complete reabsorption of glucose. The reabsorption of glucose in the proximal tubule is achieved under the joint effects of SGLT1, SGLT2, and GLUT2. SGLT1 is expressed on the apical membrane of epithelial cells in the S3 segment proximal tubule; SGLT2 is expressed on the apical membrane of epithelial cells in S1 and S2 segments proximal tubule, and GLUT2 is expressed on the basolateral membrane of cells at S1, S2, and S3 segments [42, 43]. During the process of glucose transportation in kidneys, glucose is actively absorbed through SGLT1 and SGLT2 expressed on the brush border membrane (BBM) of the proximal tubule by electrochemical driving mechanisms, which increases the cellular glucose concentration [44]. The glucose is then transported out of cells by GLUT1 and GLUT2 expressed on the lateral basilar membrane [45], which is similar to the transportation mode of glucose in the small intestine.

In a previous study, streptozotocin was used to induce the diabetes model in rats, and the consequent investigations showed that the elevation of glucose concentration in plasma or liquid in renal tubule promotes the expression of GLUT2 and influences the transportation capability of GLUT2 as well. The inappropriate elevation of GLUT2 in BBM of proximal tubule may indicate that high blood glucose could influence the inherent activity of GLUT2 protein and thus alter the absorption of glucose by kidneys [46]. In another study, SGLT1, SGLT2, and GLUT2 genes were knocked out in mice, and then the 24-h excretion of glucose was measured. The findings showed that SGLT1 knockout mice absorbed 98% of loading glucose. In addition, 67% of glucose was reabsorbed in SGLT2 knockout mice, and massive glucosuria was induced in mice with functional GLUT2 knockout [47, 48]. Subjects with Fanconi syndrome, an inherited metabolic disease, have massive glucosuria, which is somewhat associated with a missense mutation of GLUT2 in the kidney [49]. These findings demonstrate that the normal expression of GLUT2 in kidneys has an important role in the reabsorption of glucose in kidney tubules.

## GLUT2-related blood glucose surveillance and regulation

### GLUT2 and central nervous system

GLUT2 is expressed in the central nervous system [50, 51]. The immunohistochemical assay on mice brain tissue showed GLUT2 protein expression in most brain structures, as well as in the third ventricle formed by neurons, astrocytes, endothelial cells, and glial cells [52, 53]. Studies have also suggested that abnormal expression of the GLUT2 gene could influence the development of the brain and nervous system. A zebrafish model showed that mutation of GLUT2 induces severe abnormalities in the development of metacoel in embryos by affecting the structures of the midbrain and afterbrain. GLUT2 knockout can damage the development of GAB AND progenitor cells of GLUT in afterbrain. Also, GLUT2 can promote the uptake and acquirement of glucose, thus having an important role in the processes of brain development. GLUT2 protein in brain structures participates in the sensing of glucose in the brain [54]. Knocking out GLUT2 in the nervous system could lead to inhibitory effects of glucose on the parasympathetic nerve. Moreover, it could impair the regulation of the quality and functions of pancreatic β cells [55].

#### Feeding behavior and body temperature regulation

Wan et al. found that the injection of specific antisense oligodeoxynucleotides into the brain of mice through a carotid artery or lateral ventricle could successfully silence the expression of GLUT2 in the brain. The findings showed reduced body weight and impaired insulin responses to blood glucose changes in the carotid artery in the mice without changing the feeding conditions [56]. In another study, RIP GLUT1;GLUT2^-/-^ mouse model (in which GLUT1 gene was expressed, while homologous genes of GLUT2 were knocked out) underwent intraventricular injection of glucose and 2-DG, after which the feeding response disappeared, which is associated with the disturbance of feeding center and mechanisms suppressing feeding center, and the mechanism is associated with the adrenocortical pathway [14]. The thermoregulation mechanisms in mice of the same model also vary. Compared to control mice, injection of glucose and 2-DG into the lateral ventricle induces hypothermia in gene knockout mice, which is associated with the reduced sympathetic activation of uncoupling protein-1 and deiodinase-2 in brown adipose tissue (BAT) [57]. These findings demonstrate that GLUT2 in CNS could regulate feeding behaviors and body temperature through the adrenocortical pathway.

#### Sensing of glucose and hypoglycemia regulation in nerve

Tarussio et al. studied mice with inactivation of GLUT2 in the nervous system (NG2KO mice, and found no abnormalities in metabolism and growth. However, after the injection of glucose into the lateral ventricle, the activation rate of the parasympathetic nerve in NG2KO mice was lower than in controls. Yet, the sympathetic nerve activities were not significantly different between the two groups during basic activities; however, when blood glucose was increased, the sympathetic nerve activities were not significantly inhibited [55]. This further suggested that GLUT2 has a certain role in regulating sympathetic nerves on blood glucose regulation in the system.

Expression of GLUT2 fluorescent protein was induced in neurons of mice (Glut2cre;Rosa26tdTomato), and then a rapid section of the brainstem was performed for patch-clamp analysis. The findings showed that GLUT2 in the solitary nucleus could be activated by hypoglycemia, while the signaling pathways mediating this event include the AMPK pathway and potassium channel [58]. Hybridization of Glut2Cre mice with Rosa26ChR2 mice resulted in the expression of channelrhodopsin-2 in the solitary nucleus, and patch-clamp analysis showed that blue-ray pulse could induce the discharge of GLUT2 expressing neurons [59]. In animal experiments with sympathetic nerve activities, GLUT2 expressing neurons in the solitary nucleus were activated, further increasing the sympathetic nerve discharge, which is a potent stimulation of glucagon release [59]. Therefore, the expression of GLUT2 on the solitary nucleus appears to exert the linking effects in mechanisms underlying hypoglycemia sensing and feedback regulation of the nervous system. When hypoglycemia occurs, neurons with GLUT2 expression firstly stimulate the increase of sympathetic nerve activities; however, the activities of a sympathetic nerve do not increase unless the blood glucose further decreases or the blood glucose concentration is even lower [15].

The investigations in RIPGlut1; Glut2^-/-^ mice showed that the glucagon secretion stimulated by hypoglycemia, as well as inhibitory effects of hyperglycemia on glucagon, disappeared. GLUT2 is not expressed in pancreatic α cells, and the abnormal secretion of glucagon indicates that the extrapancreatic GLUT2 system could participate in the secretion of glucagon [14]. Substantial blood glucose elevation was found in GLUT2 gene knockout mice [13]; however, hyperglycemia was alleviated, and blood glucose returned to normal level after ganglion block, indicating extrapancreatic systems with GLUT2 expression and participation in blood glucose regulation could regulate the activities of autonomic nerve.

#### Functions and number of pancreatic islet cells

Further studies in NG2KO mice showed that the proliferation rate of pancreatic islet cells was reduced by approximately half after weaning. Such a low proliferation rate reduced the number of pancreatic islet cells in adult mice by about 30%. However, feeding weaned NG2KO mice with high-fat carbohydrate-free food did not significantly change the proliferation rate of pancreatic islet cells [60]. Therefore, it was suggested that GLUT2 has an important role in influencing the proliferation of pancreatic islet cells, as well as the functions of mature pancreatic islet cells during the processes of transformation from dairy food (rich in fat) to carbohydrate-rich food. The findings also showed that the first phase insulin secretion in NG2KO mice was reduced. However, the first phase of insulin secretion in vitro from pancreatic islet cells acquired from young NG2KO mice was not significantly abnormal [61]. Therefore, the lack of GLUT2 in the CNS has certain influences on the stimulation of insulin secretion by glucose and the pancreatic islet cells in adulthood. Such influences could be more substantial by high-fat food stimulation and are also associated with blood glucose elevation to a certain extent.

### GLUT2 and glucose sensors in the hepatic portal vein

The hepatic portal vein is mainly innervated by an afferent branch of the vagus nerve [62]. Absorption of glucose in the small intestine after food intake could activate the vagus nerve, which in turn innervates the hepatic portal vein to exert some physiological effects. Some studies showed that after the inactivation of glucose sensors in the hepatic portal vein, intraperitoneal injection of glucose in mice could inhibit the first phase secretion of insulin. However, the first phase of insulin secretion in vitro was normal in pancreatic islets obtained from the same mice. GLUT2 is a uniporter in the first phase of insulin secretion. These findings indicate that activating indirect signals at the area where glucose enters the hepatic portal vein in vivo could be further transduced to pancreatic β cells through the nervous system and then influence the first phase secretion of insulin through the GLUT2 protein. Still, after the insulin was isolated from the system, pancreatic β cells could not receive signals from the hepatic portal vein area, and thus the first phase secretion of insulin is normal [63]. After infusing glucose into the portal vein of RIPGlut1; Glut2^-/-^ mice, the findings showed that GLUT2 protein was required for functions of signals of the glucose-sensing system in the hepatic portal vein, as well as signals stimulating the utilization of glucose by peripheral tissues.

### GLUT2 and bile acid

Burcelin et al. investigated the impact of *GLUT2* gene inactivation on the regulation of hepatic glucose metabolism during the fed to fast transition and found no abnormalities in systemic glucose homeostasis; yet, the mice gradually developed glucose intolerance due to GSIS deficiency [64]. However, pancreatic cell numbers and insulin levels did not substantially change. To validate whether there were abnormalities in signal transduction between liver and pancreatic β cells, investigators divided mice into a fasting group, a regular feeding group (control group), and an LG2KO group (study group, in which the liver *GLUT2* gene was inactivated), after which the genomic expression was measured. The findings showed expression modifications of glycolysis and fatty acid synthase genes in the LG2KO group, and most cholesterol synthesis-related genes were downregulated, while no significant changes were observed in the control group. Bile acid is the precursor of cholesterol; the measurement of bile acid in feces and plasma further showed that bile acid in feces was 30% lower in the LG2KO group than in the control group, and the plasma bile acid level was also lower. Furthermore, the pancreatic islets from the study group were incubated in bile acid for 24 h, and the findings showed that GSIS functions improved significantly. However, no such changes were found in pancreatic islet cells of the Fxr gene (which encodes the nuclear receptor of bile acid) in knockout mice [64].

Bile acid exerts the sensitization effects on regulating GSIS functions through the Fxr-dependent potassium channel [65]. Therefore, it is believed that bile acid links hepatocyte metabolism with pancreatic β cell functions.

## Discussion

GLUT2 not only participates in glucose absorption and metabolism through the effects in pancreatic β cells, liver, small intestine, and kidneys but also is expressed in CNS and various glucose sensors to exert systemic or local glucose regulation effects. Variations of GLUT2 are associated with various endocrine and metabolic disorders. Whole-genome analysis showed that GLUT2 variations are associated with fasting blood glucose (FPG) impairment [66, 67], elevated risk of progression from FPG impairment to diabetes, and development of type 2 diabetes [66, 68, 69]. Sites of GLUT2 have very important effects on serum cholesterol levels. In addition, the low-risk GLUT2 allele was significantly associated with the risks of cardiovascular diseases [70].

Other studies showed that subjects with Thr110Ile encoded by GLUT2 with polymorphism favored high-glucose food [71]. GLUT2 controls the number of pancreatic islet cells and the volume of relevant organs during the development phase of the body; after maturation, GLUT2 regulates blood glucose through the CNS and local blood glucose regulation system. In addition, the expression of GLUT2 in vital organs participates in blood glucose metabolism and transportation to maintain blood glucose homeostasis. However, the association between GLUT2 in the CNS and various organs are still not fully understood. In addition, whether GLUT2 mediated regulatory system or glucose transportation of GLUT2 in organs is first activated during blood glucose abnormalities. It is also unclear whether the risk of complications, such as dyslipidemia and cardiovascular diseases, is higher in subjects with GLUT2 gene mutations than in other people after the development of diabetes. Providing answers to such questions could help elucidate the mechanism underlying the development and progression of diabetes and the occurrence of complications, which could be used to prevent and treat diabetes and prognoses in patients.

## Conclusion

GLUT2 covertly regulates blood glucose metabolism-related behaviors, influencing the development of blood glucose-related organs and directly participating in blood glucose metabolism and regulation. However, the mechanisms underlying these processes and influencing factors need to be further investigated.

## Supplementary Information

Below is the link to the electronic supplementary material.

Supplementary material 1 (PDF 3180.5 kb)

Supplementary material 2 (CAJ 2813.7 kb)

## Data Availability

All data generated or analysed during this study are included in this published article.
