# Supplementary material for: The role of GLUT2 in glucose metabolism in multiple organs and tissues
Source: Mol Biol Rep. 2023 Jun 26;50(8):6963–74. doi: 10.1007/s11033-023-08535-w (PMC10374759; doi:10.1007/s11033-023-08535-w)
Supplement: Supplementary file 1 — Supplementary material 1 (PDF 3180.5 kb) [file 11033_2023_8535_MOESM1_ESM.pdf]

分类号：R3　　　　　　　　　　　　　　　　　　　　密级：公开

蘭州大學

研　究　生　学　位　论　文

论文题目（中文）	雌二醇和黄体酮联用对 STZ 所致卵巢切除小鼠糖尿病发生的预防作用研究
论文题目（外文）	The study of preventative effects of estradiol combined with progesterone on streptozotocin-induced diabetes in ovariectomized mice
研究生姓名	剡　媛
学科、专业	生物学 • 生理学
研究方向	内分泌代谢生理
学位级别	硕　士
导师姓名、职称	李红芳　教授
论文工作起止年月	2016 年 3 月至 2017 年 9 月
论文提交日期	2018 年 4 月
论文答辩日期	2018 年 5 月
学位授予日期	2018 年 6 月

校址：甘肃省兰州市

万方数据

学　　院：基础医学院　　　　学　　号：220150914470

学生姓名：刿媛　　　　导师姓名：李红芳

学科名称：生物学·生理学

论文题目：雌二醇和黄体酮联用对 STZ 所致卵巢切除小鼠糖尿病发生的预防作用研究

原 创 性 声 明

本人郑重声明：本人所呈交的学位论文，是在导师的指导下独立进行研究所取得的成果。学位论文中凡引用他人已经发表或未发表的成果、数据、观点等，均已明确注明出处。除文中已经注明引用的内容外，不包含任何其他个人或集体已经发表或撰写过的科研成果。对本文的研究成果做出重要贡献的个人和集体，均已在文中以明确方式标明。

本声明的法律责任由本人承担。

论文作者签名：刿媛　　　　日　期：2018.6.10

关于学位论文使用授权的声明

本人在导师指导下所完成的论文及相关的职务作品，知识产权归属兰州大学。本人完全了解兰州大学有关保存、使用学位论文的规定，同意学校保存或向国家有关部门或机构送交论文的纸质版和电子版，允许论文被查阅和借阅；本人授权兰州大学可以将本学位论文的全部或部分内容编入有关数据库进行检索，可以采用任何复制手段保存和汇编本学位论文。本人离校后发表、使用学位论文或与该论文直接相关的学术论文或成果时，第一署名单位仍然为兰州大学。

本学位论文研究内容：

☑可以公开

☐不宜公开，已在学位办公室办理保密申请，解密后适用本授权书。

（请在以上选项内选择其中一项打“√”）

论文作者签名：刿媛　　　　导师签名：李红芳

日　　期：2018.6.11　　　　日　　期：2018.6.11

万方数据

雌二醇和黄体酮联用对 STZ 所致卵巢切除

小鼠糖尿病发生的预防作用研究

中文摘要

目的：雌激素和孕激素与糖尿病发生关系密切，其在糖尿病发生发展过程中的作用与机体所处的生理状态及血中雌、孕激素水平有关，而且二者联用作用如何还不完全清楚。因此，本文通过腹腔注射链脲佐菌素（Streptozotocin, STZ），建立小鼠 1 型糖尿病模型，探讨雌、孕激素在糖尿病发生发展中的相互作用和关系，为糖尿病诊治及性激素在糖、脂代谢中的作用提供理论依据。

方法：购买雌性昆明小鼠，切除卵巢，术后恢复一周，随机分为 11 组，分别为：①卵巢切除组（OVX），②卵巢切除+腹腔注射 STZ（OVX+STZ），③卵巢切除+腹腔注射 STZ+皮下注射 0.01mg/kg 体重 17β-雌二醇（E0.01），④卵巢切除+腹腔注射 STZ+皮下注射 0.1mg/kg 体重 17β-雌二醇（E0.1），⑤卵巢切除+腹腔注射 STZ+皮下注射 1mg/kg 体重 17β-雌二醇（E1），⑥卵巢切除+腹腔注射 STZ+皮下注射 0.1mg/kg 体重黄体酮（P0.1），⑦卵巢切除+腹腔注射 STZ+皮下注射 2mg/kg 体重黄体酮（P2），⑧卵巢切除+腹腔注射 STZ+皮下注射 8mg/kg 体重黄体酮（P8），⑨卵巢切除+腹腔注射 STZ+皮下合并注射 0.1mg/kg 体重雌二醇和 0.1mg/kg 体重黄体酮组（EP0.1），⑩卵巢切除+腹腔注射 STZ+皮下合并注射 0.1mg/kg 体重 17β-雌二醇和 2mg/kg 体重黄体酮（EP2），⑪卵巢切除+腹腔注射 STZ+皮下合并注射 0.1mg/kg 体重 17β-雌二醇和 8mg/kg 体重黄体酮（EP8）。STZ 按每天 50mg/kg 体重的剂量连续腹腔注射 5 天，同时隔天皮下注射黄体酮、17β-雌二醇或联合注射 17β-雌二醇和黄体酮。每周测血糖和体重 1 次。给药五周后，进行糖耐量和胰岛素抵抗实验，同时部分小鼠摘眼球采血后颈椎脱臼处死，分别冻存和固定组织，采用 HE 染色对胰岛细胞进行观察，利用化学发光法和 Elisa 技术检测血清中黄体酮、17β-雌二醇、C 肽（C-Peptide）及胰岛素（Insulin）水平，通过 RT-PCR 及免疫荧光技术检测骨骼肌糖转运蛋白 4（GLUT4），肝脏糖转运蛋白 2（GLUT2），葡萄糖激酶（Gck），葡萄糖-6-磷酸酶（G-6-P），磷酸烯醇式丙酮酸羧基酶（PCK）及胰岛素受体（Insulin receptor，IR）的表达水平。

万方数据

结果：STZ 所致的 1 型糖尿病小鼠血糖明显升高，体重下降，出现糖尿症；糖耐量明显受损，胰岛细胞萎缩，C 肽和胰岛素分泌明显减少；骨骼肌 GLUT4 和肝脏 Gck 表达下调，肝脏 GLUT2、G-6-p 和 PCK 表达明显上调，但无明显的胰岛素抵抗，胰岛素受体表达也无明显变化。

中剂量（0.1mg/kg 体重）和大剂量（1mg/kg 体重）17β-雌二醇可明显延缓 STZ 所致小鼠 1 型糖尿病的发生，使其血糖降低；低剂量（0.01mg/kg 体重）17β-雌二醇无明显影响。中剂量（0.1mg/kg 体重）的 17β-雌二醇还可改善糖耐量，保护胰岛细胞，使 C 肽和胰岛素分泌增加，骨骼肌 GLUT4 和肝脏 Gck 表达上调，肝脏 GLUT2、PCK 和 G-6-P 表达显著下调。

中剂量（2mg/kg 体重）黄体酮单独给药可明显延缓 STZ 所致小鼠 1 型糖尿病的发生，保护胰岛细胞，使 C 肽和胰岛素分泌增加，降低血糖；黄体酮还可使骨骼肌 GLUT4 和肝脏 Gck 表达上调，肝脏 GLUT2、PCK 和 G-6-P 表达显著下调，但剂量不同，作用效果不同，且不表现剂量效应关系，对糖耐量也无明显改善作用。

低剂量（0.1mg/kg 体重）和中剂量（2mg/kg 体重）黄体酮与 17β-雌二醇（0.1mg/kg 体重）联合给药对 STZ 所致小鼠 1 型糖尿病发生的延缓作用、胰岛细胞的保护作用、C 肽和胰岛素的分泌及血糖降低作用均具有一定的协同效应；但对于骨骼肌 GLUT4 及肝脏 GLUT2、Gck、PCK 和 G-6-P 的表达，低剂量（0.1mg/kg 体重）和大剂量（8mg/kg 体重）黄体酮与 17β-雌二醇 0.1mg/kg 体重联用主要表现出拮抗效应，而中剂量（2mg/kg 体重）黄体酮与 0.1mg/kg 体重 17β-雌二醇联用主要表现出协同效应。

结论：

1. 17β-雌二醇和黄体酮单独给药均可延缓 STZ 所致小鼠 1 型糖尿病的发生，但不表现剂量效应关系；皮下注射中剂量的 17β-雌二醇（0.1mg/kg 体重）和黄体酮（2mg/kg 体重）可使血药浓度达到生理剂量范围，可明显抑制 STZ 所致小鼠血糖的升高。

2. 17β-雌二醇和黄体酮延缓糖尿病发生机制是多方面的，可能与其保护胰岛细胞、促进胰岛素释放、改变骨骼肌葡萄糖转运及肝脏糖代谢有关。

3. 生理剂量的黄体酮与 17β-雌二醇联用对 17β-雌二醇延缓糖尿病的发生及影响糖转运和糖代谢具有一定的协同效应，可加强 17β-雌二醇对糖尿病的预防作用。

关键词：17β-雌二醇，黄体酮，1 型糖尿病，胰岛素抵抗，糖耐量，葡萄糖转运体 2、4，磷酸烯醇式丙酮酸羧基酶，葡萄糖激酶，葡萄糖 6-磷酸酶

万方数据

The study of preventative effects of estradiol combined with progesterone on streptozotocin-induced diabetes in ovariectomized mice

Abstract

Objective: Estrogen and progesterone are closely related to the occurrence of diabetes. Their role in the development of diabetes is related to the physical state of the body and the levels of estrogen and progesterone in the blood. It is not entirely clear how the estrogenic and progestogenic effects are combined. Therefore, the present study was to establish a model of type 1 diabetes mellitus in mice by intraperitoneal injection of streptozotocin (STZ) to investigate the interaction and relationship between estrogen and progesterone in the development of diabetes mellitus, and to provide a theoretical basis for the diagnosis and treatment of diabetes and the role of sex hormones in glucose and lipid metabolism.

Methods: Female Kunming mice were purchased and their ovaries were removed and recovered for one week. They were randomly divided into 11 groups: ① ovariectomy group (OVX), ② ovariectomy + intraperitoneal injection of STZ (OVX+STZ), ③ ovariectomy + intraperitoneal injection of STZ + subcutaneous injection of 17-β estradiol at the dose of 0.01 mg/kg body weight (E0.01), ④ ovariectomy + intraperitoneal injection of STZ + subcutaneous injection of 17-β estradiol at the dose of 0.1 mg/kg body weight (E0.1), ⑤ variectomy + intraperitoneal injection of STZ plus subcutaneous injection of 17-β estradiol at the dose of 1 mg/kg body weight (E1), ⑥ ovariectomy + intraperitoneal injection of STZ + subcutaneous injection of progesterone at the dose of 0.1 mg/kg body weight (P0.1), ⑦ ovariectomy + intraperitoneal injection of STZ + subcutaneous injection of progesterone at the dose of 2 mg/kg body weight (P2), ⑧ Ovariectomy + intraperitoneal injection of STZ + subcutaneous injection of progesterone at the dose of 8 mg/kg body weight (P8), ⑨ ovariectomy + intraperitoneal injection of STZ + subcutaneous injection of 17-β estradiol at the dose of 0.1 mg/kg body weight and progesterone at the dose of 0.1 mg/kg body weight (EP0.1), ⑩ Ovariectomy + intraperitoneal injection of STZ + subcutaneous injection of 17-β estradiol at the dose of 0.1 mg/kg body weight and

万方数据

progesterone at the dose of 2 mg/kg body weight (EP2), ⑪ Ovariectomy + intraperitoneal injection of STZ + subcutaneous injection of 17-β estradiol at the dose of 0.1 mg/kg body weight and progesterone at the dose of 8 mg/kg body weight (EP8). STZ was continuously administered intraperitoneally at the dose of 50 mg/kg body weight per day for 5 days, while progesterone, 17-β estradiol, or a combination of 17-β estradiol and progesterone was injected subcutaneously on alternate days. Blood glucose and body weight were measured once a week. Five weeks after administration of sex hormone, glucose tolerance and insulin resistance tests were performed. At the same time, the blood was taken from the eyeball of some mice and then the other mice were dissociated and killed, and their tissues were frozen and fixed respectively. HE staining was used to observe the islet cells; Chemiluminescence and Elisa techonology were used to detect the serum levels of 17β-estradiol, progesterone, C peptide and insulin; RT-PCR and immunofluorescence techniques were used to detect the expression levels of skeletal muscle glucose transporter 4 (GLUT4), liver glucose transporter 2 (GLUT2), glucokinase (Gck), Glucose-6-phosphatase (G-6-P), phosphoenolpyruvate carboxylase (PCK) and insulin receptor (IR).

Results: The blood glucose was significantly increased, the body weight increased slowly and there appeared obvious glucosuria in STZ-induced type 1 diabetic mice; Glucose tolerance was significantly impaired, the number of islet cells was decreased, the blood concentrations of C-peptide and insulin were also significantly reduced; skeletal muscle GLUT4 and liver Gck expressions were decreased and the expressions of GLUT2, G-6-P and PCK in liver were significantly up-regulated, but there were no obvious change in insulin resistance and insulin receptor expression between the groups of OVX+STZ and OVX .

Medium-dose (0.1mg/kg body weight) and high-dose (1mg/kg body weight) 17β-estradiol could significantly delay the development of type 1 diabetes mellitus in mice caused by STZ. Meanwhile, the value of blood glucose was markedly declined; low dose (0.01mg/kg body weight) 17β-estradiol showed no significant effect on blood glucose. Medium-dose (0.1mg/kg body weight) 17β-estradiol also improved glucose tolerance, protected islet cells, promoted C-peptide and insulin secretion, increased skeletal muscle GLUT4 and liver Gck expressions, significantly down-regulated the liver GLUT2, PCK, and G-6-P mRNA expressions.

Administration of moderate dose (2mg/kg body weight) of progesterone alone could effectively decrease the glucose level in STZ-induced diabetes mice, protected

万方数据

islet cells, increased the level of C-Peptide and insulin; progesterone could also up-regulate the mRNA expressions of GLUT4 in skeletal muscle and Gck in liver, down-regulate the expressions of GLUT2, PCK, and G-6-P mRNA in the liver. The different administration doses showed the different effects on STZ-induced diabetes mice. There was no dose dependent effect and also had no apparent improvement in glucose tolerance.

The combination of low dose (0.1mg/kg body weight) or moderate dose (2mg/kg body weight) of progesterone with 0.1 mg/kg 17β-estradiol delayed the development of type 1 diabetes in mice induced by STZ, protected islet cells, promoted C-peptide and insulin secretion, decreased the level of blood glucose, and showed a certain synnergistic effects; but for the expressions of skeletal muscle GLUT4 as well as the liver GLUT2, Gck, PCK and G-6-P mRNA, when the low dose (0.1mg/kg body weight) or high dose (8mg/kg body weight) progesterone respectively combined with 0.1 mg/kg body weight 17β-estradiol there were apparent the antagonistic effects. However, the combination of medium dose (2 mg/kg body weight) progesterone and 0.1 mg/kg body weight17β-estradiol showed a synergistic effect.

Conclusion:

1. Estradiol and progesterone alone can delay the occurrence of type 1 diabetes in mice caused by STZ, but there is no the dose-response relationship; the middle dose of estradiol (0.1mg/kg body weight) and progesterone (2mg/kg body weight) injected subcutaneously can make the plasma concentration of estradiol and progesterone reach the physiological dose range, which shows significant inhibitory effect in the increase of mice blood glucose induced by STZ.

2. The mechanisms of estradiol and progesterone in delaying diabetes mellitus are various, which probably relate to the protection of islet cells, the promotion of insulin release and the changes of skeletal muscle glucose transport and liver glucose metabolism.

3. The physiological dose of progesterone combined with estradiol has a certain synergistic effect on the delaying effect of diabetes and the changes of glucose transport and glucose metabolism caused by estradiol in STZ-induced diabetes, and can enhance the preventive effect of estradiol on diabetes.

Key words: 17β-estradiol, progesterone, type 1 diabetes, insulin resistance, glucose tolerance, glucose transporter 2 and 4, phosphoenolpyruvate carboxylase, glucokinase, glucose 6-phosphatase

万方数据

目　录

中文摘要......III

Abstract......V

第一章　前　言......1

1.1 雌激素及其作用机制......1

1.1.1 雌激素的来源......1

1.1.2 雌激素受体作用机制......1

1.2 雌激素与能量代谢......2

1.2.1 雌激素在下丘脑能量平衡中的作用......2

1.2.2 雌激素与瘦素的相互作用......3

1.2.3 雌激素与神经肽 Y 的相互作用......3

1.3 雌激素与糖尿病......4

1.3.1 糖尿病概述......4

1.3.2 雌激素和胰岛素敏感性......5

1.3.3 雌激素与胰岛β细胞功能的关系......6

1.4 雌激素疗法与应用前景......8

第二章　材料和方法......10

2.1 实验动物......10

2.2 实验仪器......10

2.3 实验试剂......11

2.4 实验方法......12

2.4.1 动物实验......12

2.4.2 ELISA 实验......13

2.4.3 RT-PCR 实验......13

2.4.4 免疫荧光......16

2.4.5 Western Blot 实验......17

第三章　结　果......20

3.1 1 型糖尿病小鼠的建立......20

3.2 17β-雌二醇对糖尿病小鼠空腹体重和血糖的影响......22

3.3 黄体酮对糖尿病小鼠空腹体重和血糖的影响......23

3.4 17β-雌二醇和黄体酮联用对体重和血糖的影响......24

万方数据

3.5 17β-雌二醇和黄体酮对糖尿病小鼠糖耐量的影响......................................25
3.6 17β-雌二醇和黄体酮对糖尿病小鼠胰岛的影响..26
3.7 血清雌二醇和黄体酮及 C 肽、胰岛素水平检测.. 28
3.8 17β-雌二醇和黄体酮对糖尿病小鼠胰岛素受体表达的影响......................29
3.9 17β-雌二醇和黄体酮对 GLUT mRNA 表达的影响......................................31
3.9.1 17β-雌二醇和黄体酮对骨骼肌 GLUT4 mRNA 表达的影响............ 31
3.9.2 17β-雌二醇和黄体酮对肝脏 GLUT2 mRNA 表达的影响................ 32
3.10 17β-雌二醇和黄体酮对肝脏糖代谢酶表达的影响....................................33
3.10.1 17β-雌二醇和黄体酮对肝脏 Gck mRNA 表达的影响.................... 33
3.10.2 17β-雌二醇和黄体酮对肝脏 G-6-P 和 PCK mRNA 表达的影响...34
第四章 讨 论...35
4.1 小鼠 1 型糖尿病模型...35
4.2 雌二醇对 STZ 所致小鼠 1 型糖尿病发生的延缓作用..................................35
4.3 黄体酮对 STZ 所致小鼠 1 型糖尿病发生的延缓作用..................................36
4.4 17β-雌二醇和黄体酮联用对 STZ 所致小鼠 1 型糖尿病发生的延缓作用.36
4.5 17β-雌二醇和黄体酮延缓糖尿病发生与胰岛β细胞的保护有关................36
4.6 17β-雌二醇和黄体酮延缓糖尿病发生与促进糖转运有关..........................37
4.7 17β-雌二醇和黄体酮延缓糖尿病发生与肝脏糖代谢限速酶表达有关......38
第六章 结 论...39
参考文献...40
附 录 50
硕士期间科研成果...52
致 谢...53

万方数据

第一章 前 言

1.1 雌激素及其作用机制

1.1.1 雌激素的来源

17β-雌二醇（17β-estradiol, E2 或 E），是循环雌激素最丰富的形式，是女性的主要激素，主要由绝经前妇女的卵巢合成和分泌。是由卵巢雄烯二酮的芳构化的雌酮（Estrone, E1）转化产生。在月经周期正常的女性中，E2 作为一种内分泌激素，作用于远处的靶组织。另外两种天然存在形式的丰度较低，雌酮（Estrone, E1）和雌三醇（Estriol, E3），而第三种形式雌四醇（Estetrol, E4）仅在怀孕期间产生[1]。少量 E2 也可由其他组织产生，包括脂肪、脑和骨组织以及血管内皮和主动脉平滑肌细胞[2]。然而，绝经后妇女的卵巢不能产生 E2。男性自然低循环水平的 E2，不能作为循环激素发挥作用。绝经期女性体内的 E2 在性腺以外的组织，如乳腺、脑、肌肉、骨骼和脂肪组织中作为旁分泌或内分泌因子局部作用合成[3]。因此，在绝经后的女性和正常男性体内，E2 不是起主要作用的循环雌激素。E2 功能依赖于来自雄激素循环源的雌激素生物合成。因此，在这些个体中，E2 作用的主要驱动因素是雄激素对雌激素的芳构化。因此，E2 的组织代谢或失活也是控制细胞雌激素作用的基本参数[4]。组织雌激素磺基转移酶（Estrogen sulfotransferase, EST）是雌激素作用的关键介质。EST 是一种胞质酶，通过将磺酸基与雌激素结合[5]，从而阻止与雌激素受体的结合并增强尿中激素的排泄，在靶细胞中提供分子开关，从而抑制雌激素活性。

1.1.2 雌激素受体作用机制

对雌激素生殖行为的早期研究建立了经典的雌激素受体（Estrogen receptor, ER）作为配体激活的转录因子的范例[6]。ER 调节基因转录是一个高度动态的过程。 ER 以两种主要形式存在，即 ERα和 ERβ，它们各自具有多种亚型，并显示出不同的组织表达模式和功能[7]。ER 作用的典型“基因组”机制通常在数小时内发生，引起靶基因的激活或抑制。在这个典型的信号通路中，配体激活的 ER 从其分子伴侣热休克蛋白中解离出来，并作为二聚体直接结合到靶基因启动子中的雌激素应答元件（Estrogen response element, ERE）上，或间接结合到激活蛋白 1 或与特异性蛋白 1 应答元件的 DNA 结合[5]。与 ER 二聚体和辅因子（共激活剂或共抑制剂）相互作用以调节基因表达。而作用于不同反应元素的 ERs 会产生不同的生理作用。例如，当通过激活蛋白 1 位点连接时，ERα表现出 E2 依赖性转录的激活，而 E2 结合 ERβ对转录没有影响[8]。另外，根据配体的特性和浓度，形成 ERα-ERα同源二聚体，ERβ-ERβ同源二聚体或

万方数据

ERα-ERβ异二聚体，其中 ERα在异二聚体形成中起主导作用[5]。当这些受体单独存在于细胞中，E2 结合的 ERα和 ERβ有重叠结合位点。当两种 ER 都存在时，ERα和 ERβ之间的共享点却很少。每个 ER 会限制另一个 ER 结合位点的占用率[9]。配体激活的 ER 以循环方式促进转录。只要 E2 存在，ER 转录复合物似乎不停地与目标启动子结合、分离，并在两种状态之间来回转换。ER 转录复合体的定期循环可能代表了一种机制，可以持续评估和适应外部环境[6]。

虽然生殖功能主要通过经典的核 ER 作为配体激活的转录因子介导，但与能量代谢有关的 ER 作用的大部分还涉及核外 ERs，间接调节基因表达或独立于核发挥作用。E2 可以通过核外和膜相关形式的 ER 在数秒或数分钟内快速激活信号发挥作用[9]。ERα和 ERβ定位于小窝穴，与其他信号分子聚集在一起，从而促进相互作用和快速的细胞内信号。这些信号蛋白包括 G 蛋白、生长因子受体、酪氨酸激酶（Src）、连接蛋白（Selective estrogen receptor modulator, MNAR）和 G 蛋白偶联受体。这种多蛋白复合物为膜 ER 提供必要的相互作用以激活生长因子受体和 G 蛋白。反过来，这些 E2 诱导的快速信号通过磷酸化修饰蛋白质功能。它们也可以调节基因表达，从而调节蛋白质的产生。在心血管系统中，骨、神经系统和胰腺β细胞的核外 ER 激活磷酸化转录因子的蛋白激酶，促进 ERs 的核移位和随后的靶基因的激活[10]。

1.2 雌激素与能量代谢

1.2.1 雌激素在下丘脑能量平衡中的作用

随着女性进入更年期，循环雌激素水平下降，伴随能量平衡的改变，导致腹内脂肪增加[11]。动物卵巢切除（Ovariectomy, OVX）会导致肥胖症[12,14]，雌激素替代治疗可以预防肥胖[15]。尽管 OVX 诱发的啮齿动物食物摄入量暂时增加[16,17]，E2 替代可减少食物摄入量，但食欲过盛并不能完全解释 OVX 后代谢和肥胖的变化[14]。事实上，在两性小鼠中并不是食欲过盛或能量消耗减少，而由芳香酶失活引起 E2 合成减少促进了肥胖症的发生[18]。同样，雄性和雌性小鼠的 ERα缺乏症通过减少能量消耗和轻微增加食物摄入量，导致体重增加引起肥胖[19,20]。内源性 E2 通过增加能量消耗来促进体重动态平衡，外源性雌激素可通过影响能量摄入和能量消耗来促进能量平衡[21]。下丘脑是中枢神经系统中控制食物摄入，能量消耗和体重稳态的一个区域。所有这些特征的显著变化可以通过损伤特定的下丘脑细胞核，如腹内侧下丘脑（Ventromedial hypothalamus, VMH）[22,23]或下丘脑外侧[24,25]区域来诱导。ERα在啮齿动物大脑中的腹内侧核（Ventromedial nucleus, VMN）、弧形核（Arcuate nucleus, ARC）、内侧视前区和室旁核[26,27]大量表达。ERβ也在下丘脑细胞核中表达，但 ERβ表达相对于 ERα很少。E2 对能量平衡的影响主要由 ERα介导。ERα基因突变引起雌性小鼠肥胖[19,28]，小

万方数据

鼠 ERα的缺失可阻断雌激素替代的抗肥胖作用。ERβ在介导雌激素的生理抗肥胖作用方面效果较差，因为与 ERα不同，ERβ的缺失不会促进肥胖[28]或与肥胖相关的任何代谢症状。这一区别与药理学研究结果一致，表明选择性 ERα激动剂丙基吡唑三醇（Propylpyrazole triol, PPT）可抑制去卵巢小鼠的食物摄入量，但选择性 ERβ激动剂二芳基丙腈在任何剂量下均未影响摄食行为[29,30]。然而，E2 还通过 ERβ抑制食物摄入，因为雌性大鼠共同注射 ERβ反义寡核苷酸可阻断脑室注射 E2 的厌食作用[31]。另外，高脂饮食（High fat diet, HFD）喂养的雌性小鼠使用 ERβ选择性激动剂可增加棕色脂肪组织中产热解偶联蛋白-1 的表达，缓解肥胖[32]。因此，在特定情况下，ERβ的激活可以抑制食物摄入并增加能量消耗。雌激素 G 蛋白耦联受体（G protein-coupled ER, GPER）在体重调节中的作用仍然需要验证。在一项缺乏 GPER 的雌性小鼠的研究中，有 4 只 GPER 突变小鼠，其中 1 只出现了肥胖表型[33]。

1.2.2 雌激素与瘦素的相互作用

瘦素已被证明是一种强大的分解代谢信号，能够抑制食物摄入并增加能量消耗。这些效应通过长型瘦素受体（leptin receptor, leprb）介导[34-36]。Leprb 定位于包括 VMN 和 ARC 在内的多个脑区域，并且 leprb 与几种参与调节食物摄取和生殖的神经肽共定位[37,38]。据报道，ARC 中的 leprb 表达与 ERα共定位[39]。此外，据报道雌激素可能通过瘦素受体基因上的 ERE 下调 ARC[40]中 leprb mRNA 的表达[41]。这两种受体广泛的下丘脑共定位，表明这些外周信号之间紧密耦合，相互作用，调节能量代谢。雌激素可能是大脑中瘦素分解代谢作用的调节剂。事实上，更高水平的 E2 与啮齿动物中枢瘦素敏感性增加有关[42,43]。尽管在发情周期中循环瘦素水平没有明显变化，但 ARC leprb 表达在发情期最高，与神经肽 Y mRNA 表达呈负相关[40]。卵巢切除使中枢瘦素敏感性降低，这种缺陷可以通过 E2 替代治疗恢复。同样，给雄性大鼠使用外源 E2 会增加中枢对瘦素的敏感性[43]。在 leprb 转录和翻译的下游，中枢瘦素敏感性差异与雌激素的存在与否相关。因此，可能有一个阈值，超过该阈值，雌激素会增强对瘦素的中枢敏感性，通过减少食物摄入并增加能量消耗，达到减轻体重的作用[16]。

1.2.3 雌激素与神经肽 Y 的相互作用

神经肽 Y（Neuropeptide Y, NPY）NPY 是一种影响合成代谢的肽。NPY 中枢给药可使食物摄入量大幅增加，并减少能量消耗和脂肪氧化[44-47]。ARC 神经元共表达 NPY mRNA 和 leprb 蛋白。瘦素缺乏或瘦素抵抗会增加 NPY（和 AgRP）mRNA。因此，瘦素是 ARC NPY 功能的关键决定因素[46]。下丘脑中的 NPY 神经元不仅影响摄食，而且影响生殖。因此，E2 可以通过调节 NPY 基因表达来调节神经内分泌系统的功能。E2 刺激 NPY 和 NPY Y1 受体 的表达和 NPY 的释放[48,49]。

万方数据

1.3 雌激素与糖尿病

1.3.1 糖尿病概述

糖尿病（Diabetes mellitus, DM）是一种影响全球数百万人的以高血糖症为特征的复杂的慢性代谢疾病。已经确定有多种形式的糖尿病，包括 1 型，2 型，3 型，新生儿和妊娠糖尿病[5]。糖尿病患者通常并发多种代谢疾病。而大多数糖尿病患者在发病早期却未得到及时确诊。糖尿病患者的血糖水平通常会随年龄增长而增加，并导致心血管疾病、中风、慢性肾衰竭、足部溃疡和眼睛受损。全球糖尿病患病率在 2000 年为 2.8%，预计到 2030 年将增至 4.4%。全球糖尿病患病人数估计将从 2000 年的 1.71 亿增加到 2030 年的 3.66 亿[5]。糖尿病导致的急性并发症包括糖尿病酮症酸中毒，这是一种危及生命的疾病，特别是 1 型糖尿病患者，细胞由于胰岛素分泌不足而无法获得机体所需的能量。当血液中的葡萄糖不能进入细胞。肾脏滤过血液中的一些糖，并通过尿液除去。但当细胞不能利用糖所提供的能量时，身体便通过分解脂肪来获取能量。这通常会使分解产生的脂肪酸进入血液，引起代谢失衡，危及生命的糖尿病酮症酸中毒。糖尿病引起的另一种急性并发症是非酮症性高渗性昏迷，当血糖水平升高时，身体试图通过将糖排入尿液来排除血液中多余的糖，最初导致排尿增加。然而，在后期阶段，排尿减少，口渴增加。机体缺乏足够的液体维持正常的生命活动，脱水可能进一步导致癫痫，昏迷，甚至死亡。

糖尿病治疗和诊断面临诸多困难，其多种急、慢性并发症风险等给临床和公共卫生带来了很大的负担。在美国，糖尿病是导致残疾的第六大原因，是导致死亡的第七大原因。在 20 岁及以上的美国成年人，糖尿病在疾病医疗保健支出中位居前列，2013 年糖尿病的诊断和治疗在医疗支出上高达 1014 亿美元[5,50]。

血液中的葡萄糖水平决定了糖尿病的严重程度，但糖尿病发病的年龄通常决定了其类型。1 型糖尿病发病早，患者年龄通常在 30 岁以下。1 型糖尿病患者占糖尿病总患病人数的 5%。早发型 1 型通常伴有遗传基因突变。1 型糖尿病也被称为胰岛素依赖性糖尿病，患者通常通过注射胰岛素控制过高的血糖水平。1 型糖尿病（DM1）是由自身免疫选择性破坏胰腺β细胞引起的一种多因素疾病，此病特征在于胰腺中的β细胞自身免疫性破坏导致胰岛素绝对缺乏，多饮、多尿、体重减轻和高血糖症[51]。1 型糖尿病患者后期完全依赖非内源性胰岛素控制血糖。对于真正的 1 型糖尿病，必须及时监测血糖水平，并通过胰岛素水平的个体化维持葡萄糖稳态[5]。

2 型糖尿病是一种迟发型疾病，多发生在年龄超过 30 岁的人群中。2 型糖尿病占糖尿病人口的 90%以上[52]。患者不能调节血液中的糖水平。遗传和环境的相互作用以及高卡路里饮食（高脂肪饮食）和缺乏运动等因素都会诱发胰岛功能异常，胰岛β细胞不能产生胰岛素或调节胰岛素抵抗，最终导致迟发型 2 型糖尿病[53]。

万方数据

3 型糖尿病已被确定为阿尔茨海默病（Alzheimer disease, AD）的一种可能形式。3 型糖尿病患者会出现认知功能障碍和氧化应激，这些都会影响葡萄糖代谢。同时，也是 AD 患者胰岛素抵抗和线粒体功能障碍的特征性表现。已有多项研究发现 AD 患者和 3 型糖尿病患者代谢变化（碳水化合物、脂质和蛋白质）和脑功能障碍之间潜在的联系[5]。

新生儿糖尿病是一种发生在六个月以内的单基因疾病。平均每 10 万-50 万新生儿就有一例此病患者。糖尿病新生儿也是由于胰岛素分泌不足而导致高血糖，这种情况经常与 1 型糖尿病混淆[54,55]。

妊娠糖尿病是妊娠期母亲发作的糖尿病，通常发生在孕后期并伴随着碳水化合物的不耐受。以下情况与妊娠母亲的妊娠糖尿病有关：1）25 岁或以上，2）超重母亲，体重指数高于 30，3）母亲患有多囊卵巢综合症，4）葡萄糖耐受不良，5）服用糖皮质激素等药物治疗哮喘或自身免疫性疾病，6）服用高血压β受体阻滞剂的孕妇或抗精神病药物[56-58]。

目前，世界各地的基础科学，制药公司和临床医生正致力于改善糖尿病患者胰岛素抵抗和胰岛素缺乏的靶点研究。已有几种食品药品监督管理局（Food and drug administration, FDA）批准的靶向糖尿病治疗药物，包括二甲双胍[59]，捷诺维（Januvia）和α-葡萄糖苷酶抑制剂药物[60]，如奥利司他（Orlistat）[61]。由于糖尿病是一种多基因疾病，研究人员正在尝试基于糖尿病个体特定遗传基因差异的个性化基因组治疗方法，而不是让基因组成不同的众多患者共用一套治疗方案。临床医生通过增加日常锻炼，无糖、低脂和新鲜水果蔬菜的饮食，从有助于糖尿病患者维持正常血压和血糖水平的生活方式着手，预防和控制该病的发生和发展。由于大多数糖尿病患者在患病早期未得到及时诊断和治疗，加强糖尿病教育，提高人们对疾病的认识，通过合理膳食和运动可在一定程度上达到预防疾病发生发展的目的。

1.3.2 雌激素和胰岛素敏感性

胰岛素抵抗是 2 型糖尿病的关键发病机制。它也是代谢综合症的一个特征。与同龄男性相比，月经史正常的绝经前妇女体重正常，胰岛素敏感性较强，是女性绝经前 2 型糖尿病发病率低的原因之一[62,63]。在高脂喂养的雄性小鼠中观察到胰岛素介导的葡萄糖利用减少 40-50％[64,65]，而体内 E2 含量正常的女性和雌性啮齿动物葡萄糖的利用没有发生减少，并且在正常含量的 E2 保护下不会出现高脂饮食和急性脂肪酸诱导的胰岛素抵抗[66,67]。更年期或 OVX 后，胰岛素敏感性急剧下降与脂肪量增加并伴随循环炎症标志物、低密度脂蛋白（Low-density lipoprotein, LDL）、甘油三酯和脂肪酸升高[11,68,69]。卵巢切除的小鼠和大鼠会出现胰岛素抵抗，运动刺激时葡萄糖在肌肉中的利用受限[70]，并且更容易受到 HFD 或脂质过量供应的有害影响。在恢复 E2 到

万方数据

生理浓度可维持正常的胰岛素作用和葡萄糖耐量[71]。生理和遗传证据都表明，当 E2 浓度紧密保持在的生理浓度范围内时，E2 和 ER 有利于啮齿动物和人的胰岛素敏感性。相反，E2 在超生理水平或 ER 过度刺激时，可诱导继发于高胰岛素血症后的肝脏胰岛素抵抗或肌肉总葡萄糖转运蛋白 4（Glucose transporter 4, GLUT4）表达减少[48,49]。此外，有 2 项研究前瞻性报道认为，绝经后妇女 E2 水平下降与 2 型糖尿病风险增加有关[73,74]。很明显，为了更好地理解类固醇激素（包括 E2, 睾酮和孕酮）对葡萄糖代谢和胰岛素的调节作用，需要对啮齿动物和人类进行额外剂量反应策略研究。

已证明在高血糖和 2 型糖尿病的胰岛素受体敲除组织中，肝胰岛素抵抗的主要作用与骨骼肌胰岛素抵抗不同[75]。然而，由于 E2 缺乏或 E2 抵抗引起的肝脏对胰岛素抵抗的直接作用还不清楚。在一项研究中，全球缺乏 ERα的雌性小鼠在骨骼肌中没有表现出胰岛素抵抗，但是在血糖正常的高胰岛素条件下，他们的确表现出胰岛素控制肝脏葡萄糖产生（HGP）的作用下降[76]。这些表明 ERα缺乏可导致肝脏胰岛素抵抗。然而，Ribas 等[20]报道，ERα缺陷的雌性小鼠在正常血糖和高胰岛素条件下会出现肝脏胰岛素敏感性的轻微改变。E2 可抑制 HFD 小鼠[77]和抗瘦素雌性小鼠[78]脂肪生成基因表达，防止甘油三酯的积累和肝脂肪的变性。虽然 ERα激动剂 PPT 不能复制这种效应[79]。然而，这种效应可由新型 ERβ激动剂复制[32]。尽管如此，E2 和 PPT 治疗仍然通过依赖部分 ERα的途径[78,79]，改善 HFD 雌性小鼠[77,80]和具有遗传瘦素抗性的肥胖雌性小鼠[78]的胰岛素抵抗。然而，ERα在肝脏中的直接作用尚不清楚。总之，这些数据表明 ERα（和可能的 ERβ）激活通过预防肝脏中的异位脂质蓄积（脂毒性）来防止肝胰岛素抵抗。ERα缺乏会影响啮齿动物的骨骼肌和肝脏的脂质稳态，从而降低胰岛素抑制 HGP 的能力并促进骨骼肌葡萄糖利用。因此，HFD 期间 ERα的活化和遗传瘦素抵抗改善了由骨骼肌中异位脂质积聚诱导的胰岛素抵抗[77,79,80]。而 ERα通过中枢机制介导胰岛素敏感性的作用仍有待确定。

1.3.3 雌激素与胰岛β细胞功能的关系

在啮齿动物模型的研究中，E2 治疗可以保护胰腺β细胞免受 T1DM 和 T2DM 相关的各种损伤。包括氧化应激，淀粉样多肽毒性，脂毒性和细胞凋亡[81]。已经在啮齿动物和人类β细胞中鉴定了三种 ERs-ERα，ERβ和 GPER。与在乳房或子宫细胞中充当配体激活的转录因子的经典核 ER 不同，β细胞 ER 主要在核外。它们通过与激酶如 Src，ERK 和 AMPK（AMP-activated protein kinase, AMPK）的胞质相互作用或通过 STAT 家族的转录因子发挥作用[81-83]。ERα的激活通过涉及 Src，ERK 和刺激核易位并结合 NeuroD1（一种促胰岛素转录因子）的胰岛素启动子途径[82]，增强葡萄糖刺激后胰岛素的生物合成[82,84]。这种作用可以通过增强胰岛素生物合成来帮助胰岛适应妊娠代谢需求的增加。ERα的激活减少了过量胰岛素从头合成脂肪酸和脂肪生成以及毒

万方数据

性脂质中间体的积累[83,85]。这种抗脂肪作用至少涉及 2 条途径。首先，核外 ERα激活并促进信号转导和转录激活因子 3（Signal transducer and activator of transcription 3, STAT3）的核易位，这导致脂肪生成的主调节因子，肝 X 受体（Liver X receptor, LXR）β及其转录靶标（Sterol regulatory element-binding protein 1c, SREBP -1c）和碳水化合物应答元件结合蛋白。抑制 LXRβ和 SREBP-1c mRNA，可能通过与质膜相关的一系列 ERα介导，并激活由 STAT3 介导的 Src[83]。在β细胞中，慢性 LXR 激活导致过量的脂肪生成，其又与脂毒性和细胞凋亡相关[86]。因此，β细胞中与 LXR mRNA 表达有关的 ER 抑制可以减少脂肪生成和预防胰岛脂毒性[83]。在第二条途径中，ERα的激活诱导 AMPK 抑制 SREBP-1c 基因和蛋白质的表达[83]。ERβ的激活似乎主要通过导致心房钠尿因子受体激活，ATP 敏感性钾通道关闭的膜通路[87]增强葡萄糖刺激的胰岛素分泌[90,91]。然而，GPER 的激活保护了β细胞免受脂质积聚[74]的影响并促进其生存[90,91]。尽管 GPER 对胰岛素生物合成没有影响，但是通过激活表皮生长因子受体和 ERK[92]，GPER 的激活也增强了葡萄糖刺激后的胰岛素分泌[90,92]。然而，已经提出 GPER 诱导 ERα36 的表达，ERα36 是 ERα经典长亚型 ERα66 的一个短亚型[93]。ERα66 和 ERα36 都在β细胞中表达[85]。因此，目前尚不清楚 GPER 介导的β-细胞效应是由于内在的 GPER 作用，还是由于 GPER 与膜表面的 ERα36 相互作用。重要的是，上述 ER 配体对β细胞存活，功能和β细胞营养稳态的有益作用都有在人类β细胞中观察到[81,85,91,94]。1 型糖尿病肥胖女性相对于健康女性表现出 E2 缺陷[95]。因此，接受胰岛移植的 1 型糖尿病女性可能已经丧失了部分内源性 E2 相关的胰岛保护，并且可能受益于短期补充的 E2。从治疗的角度来看，激素依赖性癌症的风险使雌激素普通疗法作为糖尿病β细胞衰竭的慢性治疗方法让患者望而生畏。为了使 E2 与靶向β细胞优先结合发挥有益作用，而没有一般雌激素治疗的不良影响，DiMarchi 和合作者[96]构建了一种在单个分子中结合胰高血糖素样肽-1（Glucagon-like peptide-1, GLP-1）和 E2 的新型融合肽。通过结合 GLP-1 和 E2 的药理学作用，利用同时表达 ER 和 GLP-1 受体（胰高血糖素样肽-1 受体）的胰腺β细胞，以及促胰岛素和抗细胞凋亡活性对β细胞功能和存活的协同作用。合成两种与 GLP-1 稳定结合的 E2 缀合物，该缀合物可以避免 E2 在循环过程提前释放作用于非靶细胞带来的副作用，使 E2 尽可能多地与靶细胞如胰腺β细胞结合。与 E2 稳定连接的两种融合肽，是 E2 分别和激动剂 GLP-1（aGLP1-E2）和非活性 GLP-1（iGLP1-E2）结合形成的两种缀合物，而与非活性 GLP-1（iGLP1-E2）缀合物通常与 GLP-1R（GLP-1 receptor）结合，其在药理学上虽不能激活 GLP-1R 信号传导，但它可有效将 E2 运送至β-细胞。Tiano 等人[97]在由多次低剂量注射 STZ 诱导的β细胞破坏模型中测试了 GLP1-E2 缀合物在预防胰岛素缺陷型糖尿病的效率。他们观察到 iGLP1-E2 耦联物会改善 STZ 诱导的胰岛素缺陷型糖尿病，由此证明，失活的 GLP-1 能够结合 GLP-1R 并将 E2 导向β细胞同时起到保护作用。最重要的是，

万方数据

aGLP1-E2 偶联物比 GLP-1 激动剂和 iGLP1-E2 单独预防 STZ 诱导的糖尿病更有效。与一般 E2 疗法相比，所有缀合物没有产生 E2 在妇科方面的其他副作用[97]。这些观察结果提供了在单个分子中结合 GLP-1 和 E2 的概念的证据，两者的协同作用可以保护β-细胞，而没有一般雌激素疗法相关的副作用。

1.4 雌激素疗法与应用前景

雌激素（Estrogen, E）类型和给药方法影响着其临床使用效果。在安慰剂对照实验中，如女性健康倡议（Women’s health initiative, WHI）只使用共轭马雌激素（Conjugated equine estrogen, CEE）和孕激素。然而，雌激素的生理形式是 E2，并且可用的是一些口服制剂以及用于透皮或透皮吸收的贴剂、乳膏和凝胶。与口服 HRT（Hormone replacement therapy, HRT）相比，经皮给药避免药物的肝脏代谢，从而保持更稳定的血清水平[98]。用透皮 E2 治疗会比相应剂量的 CEE 法导致相对更高的血清 E2、E1 和 E1 硫酸盐水平[99]，更年期妇女 E2 透皮给药是一种安全有效的方法模拟生理条件将激素输入机体循环[100]，而没有口服 CEE 治疗引起的代谢并发症[101]。有报道表明口服 E2 可能加剧胰岛素抵抗和增加脂肪细胞，加重心血管风险[102]。然而，透皮 E2 对胰岛素抵抗的影响很小，导致脂联素水平升高。这表明与代谢综合征的肥胖妇女口服 CEE 相比，透皮 E2 可能是更好的治疗方法。另外，口服雌激素与增加的促炎症因子（基质金属肽酶 9）相关，这种副作用是透皮用药中未观察到的[103]。对绝经后妇女进行的 100 多项随机实验，分析了 HRT 对代谢综合征的影响[104]。作者得出结论：在非糖尿病女性中，口服和透皮雌激素（含或不含孕激素）能减轻体重；减少腹部脂肪，改善胰岛素抵抗，降低低密度 /高密度脂蛋白胆固醇比值和降低血压[104]；在糖尿病妇女中，口服和透皮雌激素（含或不含孕激素）均能降低空腹血糖，改善胰岛素抵抗，并降低低密度 /高密度脂蛋白胆固醇比值。雌激素对代谢的有益作用是剂量依赖性的，并且通过加入孕激素而减少[103]。这些雌激素对代谢综合征临床特征的有益影响，可能是年轻女性进行 HRT 时死亡率和心血管疾病减少的原因[104,105]。口服雌激素疗法，特别是 CEE 导致更强的有益代谢作用。如先前在啮齿动物模型中所讨论的，口服治疗对血糖更强的作用可能是首先经肝代谢而导致更好地抑制肝胰岛素抵抗的结果[76]。然而，口服雌激素治疗也与甘油三酯升高、炎症标志物如 C-反应蛋白和凝血抑制剂如蛋白 S[104]相关。这可能增加心血管和血栓形成风险，并可能导致年长妇女开始 HRT 时心血管风险增加的原因[106-108]。相反，透皮雌激素对甘油三酯或炎症和凝血因子没有不良影响[104,109]。进一步的研究有助于确定透皮雌激素治疗是否可以安全降低绝经后妇女的心血管疾病发病率。选择性雌激素受体调节剂和芳香酶抑制剂对代谢的影响有助于确定经皮雌激素治疗是否可以安全降低绝经后妇女的心血管疾病发病率。

万方数据

雌激素为药物干预提供了抗糖尿病和肥胖的可能性。靶向雌激素作用的优点在于 E2 和 SERM 生物学和临床用药效果和毒理知识的掌握，这些知识已经从临床前模型和人体内数十年的体内研究积累起来。E2 促进能量平衡，改善体内脂肪分布，改善胰岛素抵抗（或增强胰岛素敏感性），改善β细胞功能并减少炎症。然而，雌激素所面临的挑战是，作为药物长期给药时，它们的治疗指数相对较窄。因此，糖尿病和肥胖症治疗基本进展虽然在啮齿动物中取得成功，但在扩展到临床实践时仍存在问题。然而，WHI 在 10 年后不恰当地得出结论，激素治疗的风险超过了其益处，并夸大了乳腺癌、冠心病、中风和肺栓塞与雌激素-孕激素治疗的风险。北美更年期协会指出：HRT 在更年期症状的短期治疗中发挥明显的有益作用[106,110]。因此，至少在这段时间内，可以缓解由 E2 缺陷引起的代谢功能障碍。

由于近年来肥胖和代谢综合征的急剧增加，出现了很多重要的临床问题，其中最主要的是如何增强 E2 在代谢性疾病预防和慢性疗法中的作用。显然，针对参与能量平衡和葡萄糖体内平衡的 ER，并开发雌激素样药物，使其仅产生代谢益处而没有其他副作用。这可以通过组织靶向融合蛋白的方法实现，通过使用融合肽[96,97]或通过将 GLP-1 介导的 E2 递送给具有高密度 GLP-1Rs 的组织，以最小化其带来的毒副作用，SERMs 将保持 E2 在所需组织中的有益代谢作用，同时拮抗乳房和子宫中的 ER。了解 E2 及其 ER 在胰岛细胞、骨骼肌、肝脏和脂肪组织中的功能和分子机制可为雌激素有益作用提供新的药理靶点。

万方数据

第二章　材料和方法

2.1 实验动物

雌性昆明小鼠(25±5g) 购于兰州大学实验动物中心。

2.2 实验仪器

仪器名称	销售公司
电子天平 CP114	奥豪斯仪器（上海）有限公司
pH 计 Delta320	梅特勒-托利多仪器（上海）有限公司
PCR 扩增仪	Thermo 公司
Western blot 转膜仪	Bio-rad 公司
Western blot 电泳仪	Bio-rad 公司
Odyssey 双色红外荧光成像系统	美国 LI-COR 公司
凝胶成像仪	美国 UVP
高速离心机	德国 Eppendorf
水平 DNA 电泳仪	Bio-rad 公司
4 ℃恒温冰箱	美国 Thermo
－80℃超低温冰箱	美国 Thermo
－20℃低温冰箱	中科美菱
制冰机	中科美菱
烘箱	上海恒科
摇床	德国 IKA
酶标仪	Thermo 公司
恒温培养箱	上海精宏设备公司
水浴锅	上海科伟
罗氏血糖仪	爱尔兰罗氏
荧光显微镜	日本 Olympus
空调	广东格力

万方数据

2.3 实验试剂

试剂名称	销售公司
链脲佐菌素（STZ）	Sigma 公司
17β-雌二醇	Sigma 公司
DAB 显色试剂盒	碧云天
预染 Marker	Thermo 公司
PVDF 膜	Millipore 公司
甲叉双丙烯酰胺	Amresco 公司
甘氨酸	Solarbio 公司
丙烯酰胺	Amresco 公司
beta-actin 一抗	Cst 公司
TEMED	Sigma 公司
PCR MIX	Promega 生物公司
引物	Takara 公司
Gelred	Biotium 公司
PMSF	Solarbio 公司
琼脂糖	Invitrogen 公司
过硫酸铵	Sigma 公司
cDNA 反转录试剂盒	Promega 公司
Tris 碱	Solarbio 生物公司
RIPA 裂解液	Solarbio 生物公司
Tween-20	Sigma 公司
4x 蛋白上样缓冲液	Solarbio 公司
山羊血清	北京中杉金桥
脱脂牛奶	BD 公司
荧光二抗	LI-COR 公司
胰岛素	诺和锐公司
Iusulin receptor 一抗	Abcam 公司
DAPI	Sigma 公司
抗荧光衰减封片剂	Solarbio 公司
酪蛋白	Solarbio 公司
Trizol 试剂	Invitrogen 公司

万方数据

2.4 实验方法

2.4.1 动物实验

（1）将购买的小鼠适应性喂养（室温 23 ± 2℃）一周后，禁食 12h 后称重，通过小鼠尾静脉（剪刀剪去尾尖端，弃掉第一滴血）测量术前血糖，0.3%戊巴比妥钠麻醉后行卵巢切除手术（OVX），结扎输卵管后切除卵巢，缝合时先缝内层肌肉，后缝外皮层。手术前后用碘伏棉球消毒手术部位，所有器械经高压灭菌或 75%酒精浸泡消毒后使用。

（2）术后小鼠恢复一周，禁食不禁水 12h，称重测血糖，随机分为以下 11 组（6-12 只/组）。

Groups	Abbreviation
卵巢切除组	OVX
OVX 后注射 STZ 组	OVX+STZ
OVX 后注射 STZ 和 0.01 mg/kg 体重 17β-雌二醇	E0.01
OVX 后注射 STZ 和 0.1 mg/kg 体重 17β-雌二醇	E0.1
OVX 后注射 STZ 和 1 mg/kg 体重 17β-雌二醇	E1
OVX 后注射 STZ 和 0.1 mg/kg 体重黄体酮	P0.1
OVX 后注射 STZ 和 2 mg/kg 体重黄体酮	P2
OVX 后注射 STZ 和 8 mg/kg 体重黄体酮	P8
OVX 后注射 STZ 和 0.1 mg/kg 体重 17β-雌二醇+0.1 mg/kg 体重黄体酮	EP0.1
OVX 后注射 STZ 和 0.1 mg/kg 体重 17β-雌二醇+2 mg/kg 体重黄体酮	EP2
OVX 后注射 STZ 和 0.1 mg/kg 体重 17β-雌二醇+8 mg/kg 体重黄体酮	EP8

（3）小鼠 OVX 手术一周后，开始造模，禁食不禁水 12h，连续 5 天腹腔注射链脲佐菌素（STZ，50mg/kg 体重），同时按分组隔天皮下注射雌二醇和黄体酮，给药 5 周，每周称重测血糖。

（4）给药五周后进行葡萄糖耐量和胰岛素抵抗实验，腹腔注射葡萄糖（1.5g/kg 体重）和胰岛素（1U/kg 体重）分别在 0min，30min，60min，90min，120min 测血糖。葡萄糖耐量实验前小鼠禁食不禁水 12h，胰岛素抵抗实验前小鼠禁食不禁水 4h。

（5）动物实验结束后，将小鼠摘眼球采血（血样室温静置 1-2h，4℃，3000r/min 离心取上层血清备用），颈椎脱臼处死，快速取肝脏、骨骼肌、胰腺等组织，液氮速冻后－80℃冰箱保存。

（6）动物实验试剂配制方法：

戊巴比妥钠（0.3%）：称取 0.3g 戊巴比妥钠，加入 100mL 无菌蒸馏水，搅拌溶

万方数据

解；

STZ缓冲液：称柠檬酸2.1g加入100mL无菌蒸馏水配成A液，称柠檬酸钠2.94g加入100mL无菌蒸馏水配成B液，使用时将A、B液按1:1.32的比例混合，调节pH=4.2-4.5即可；

STZ注射液：按体重（空腹，50mg/kg体重）称取STZ，溶于STZ缓冲液，避光冰浴，30min以内注射完毕；

雌二醇注射液：按体重（空腹）称取雌二醇加入少量酒精吹打（难溶）溶解后，加入芝麻油混匀，4℃保存；

黄体酮注射液：按体重（空腹）称取黄体酮直接加入芝麻油，搅拌加速溶解，4℃保存；

胰岛素注射液：按体重（空腹，1U/kg体重）调节胰岛素注射笔至所需单位胰岛素，注入一定体积的生理盐水中，混匀即可，4℃保存；

葡萄糖注射液：按体重（空腹，1.5g/kg体重）称取葡萄糖溶于灭菌水中，搅拌溶解。

2.4.2 胰岛细胞计数

取小鼠胰腺组织用4%的多聚甲醛固定，石蜡包埋切片后进行HE染色，利用光学显微镜和图像分析系统对不同处理组小鼠胰岛细胞进行观察，在10倍光镜下随机选取3-5个视野，利用图像分析系统对胰岛细胞进行计数，取平均值。

2.4.3 ELISA实验

取出血清室温解冻，按照C肽和胰岛素试剂盒说明书配制试剂加入反应后置于酶标仪450nm波长下测定OD值，通过标准曲线计算C肽和胰岛素浓度。

2.4.4 RT-PCR实验

（1）RNA提取

实验前清洗匀浆器、研钵，器械高压灭菌烘干备用。购买无酶枪头和离心管。使用75%酒精棉球擦拭桌面及移液枪等相关器械。提取RNA的关键是要抑制细胞中的RNA分解酶和防止所用器具及试剂中的RNA分解酶的污染。因此，操作者在实验中需配戴一次性干净手套和口罩。在操作过程中避免在敞口的试剂上空来回移动，所用试剂取完及时封口等等。尽可能减少空气及唾液中的 RNA 分解酶的污染。尽量使用一次性无酶塑料器皿，若用玻璃器皿，应在使用前，干热灭菌（280℃，120 min），0.1% DEPC（焦碳酸二乙酯）水溶液在室温浸泡处理12小时。然后在灭菌锅中于120℃高压灭菌20分钟以除去残留的DEPC（DEPC在高温高压后分解为二氧化碳和水）。

万方数据

提取过程中使用的无菌水需用 0.1%的 DEPC 处理后进行高温高压灭菌。RNA 提取实验所用的试剂、无菌水、器具和仪器都应专用，避免与其他实验混用后交叉污染。

1）取出冻存的组织，快速称取 （50 -100mg）后置于研钵中，加入适量液氮，待其挥发殆尽时，组织冻脆，研磨效果最佳，迅速研磨转移至提前加入 1mL 预冷 trizol 的离心管中振荡，使沉在管底的组织悬浮，转移至玻璃匀浆器，置于冰上匀浆。组织无大颗粒时即可，转移匀浆液至原来的离心管室温静置待其裂解 5min 后，转移到冷冻冰箱，待所有样本研磨匀浆完毕后，同时进行萃取；

2）加入 200μL（200μl/mLTrizol）三氯甲烷（$CHCl_3$），立即用力振荡 15s，室温静置萃取 3min 左右，12000g，4℃离心 15min；

3）吸取上层水相于 1.5mL 新离心管中，按（0.5mL/mLTrizol）加入 0.5mL 异丙醇，室温静置 10min，12000g，4℃离心 10min；

4）弃掉管内液体，此时可肉眼观察到沉在管底白色的 RNA 团，按（1 mL 75%乙醇 /mLTrizol）加入无水乙醇，涡旋振荡仪震起管底的 RNA，8000 g，4℃离心 5min；

5）弃掉液体，室温干燥 RNA，按 RNA 沉淀体积大小加入适量无酶水（Rnase-free water）将其溶解，必要时分装，分别用于测量 RNA 浓度、纯度和逆转录。

（2）cDNA 合成

选择 OD 值 260/280 在 1.8-2.0 之间的样本，用无酶水将 RNA 浓度调整到 1μg/μL，按照反转录试剂盒说明书规范操作，合成 cDNA。

1）去除基因组 DNA 反应

按下表中所列试剂在冰上配制反应所需预混液，为了保证反应液配置的准确和简便，可按照所有待反应样本数+2 的量加入试剂 1 和 2 配成 Master Mix，然后再分装到每个反应管中，后加入 RNA 样品，反应终体积 10μL。

试剂	使用量
1. 5xgDNA Eraser Buffer	2.0μL
2. gDNA Eraser	1.0μL
3. Total RNA (1.0μg/μL)	1.0μL
4. Rnase-free Water	6.0μL

加好上述溶液离心混匀后，于 PCR 仪上 42℃反应 2 min，然后取出置于冰上。

2）逆转录反应

按下表中列出的试剂在冰上配制反应预混液。同样可先按样本反应数+2 的量加入下表中的 2、3、4、5 试剂，配制 Master Mix，然后再分装 10μL 到每个反应管中。

万方数据

试剂	使用量
1. 步骤 1 的反应液	10.0μL
2. PrimeScript RT Enzyme Mix 1	1.0μL
3. RT Primer Mix	1.0μL
4. 5×PrimeScript Buffer 2（for Real Time）	4.0μL
5. Rnase-free Water	4.0μL
6. Total	20.0μL

于微型离心机离心混匀后，立即置于 PCR 仪进行逆转录反应。设置运行程序为 37℃ 15 min，85℃ 5 sec，逆转录体系可以根据个人实验的实际需要做相应调整（可扩大比例进行逆转录反应）。合成的 cDNA 需要长期保存时，于-20℃或更低温度保存，必要时分装保存，以避免反复冻融带来的负面影响。

3）特异性扩增

将反转录后得到的 cDNA 样品用无酶水稀释 3 倍，按照引物说明书用无酶水稀释引物，按下表的试剂冰上配制反应混合液。为了保证反应液配制的准确，反应时，可按反应数+2 的量加入下表中的 1、2、3、5 配制 Master Mix，然后再分装 24μL 到每个反应管中。

试剂	使用量
1. PCR GoTaq ® Green Master Mix (2X)	12.5μL
2. PCR Forward Primer（10 μmol/L）	1.0μL
3. PCR Reverse Primer（10 μmol/L）	1.0μL
4. cDNA template（<200ng）	1.0μL
5. Rnase-free Water	9.5μL

加好以上试剂，在引物适宜的退火温度和循环数条件下进行特异性扩增，新买的引物可根据引物说明书的退火温度摸最适温度和最佳循环数，温度可在 Tm 以下 3℃起递增，循环数一般在 25times 开始。

4）使用 Image J 软件对条带进行灰度分析，将分析所得灰度值通过 SPSS21.0 软件进行统计学意义分析，组间比较采用 t 检验分析。最后根据灰度平均值、标准差以及显著性 *P* 值作图，数据均以均数±标准差（$\bar{x}$±SD）表示。

万方数据

引物序列

Gene name	sequence(5'- 3')	Tm(°C)	Cycles
Actin –R	GCCACGATGGAGACATAGC	60	29
Actin– F	CATCCGTAAAGACCTCTATGCCAAC		
GLUT4 –R	ATGGAGCCACCGATCCACA	65	26
GLUT4 –F	GACGGACACTCCATCTGTTG		
GLUT2 –F	TTCCACACACACACAATGTCAGAAG	65	33
GLUT2 –R	TTGATCACACCGATGTCATAGCC		
Gck –F	AGTACGACCGGATGGTGGATG	65	28
Gck –R	TTGAGCAGCACAAGTCGTACCAG		
G-6-P –F	CAGCAACAGCTCCGTGCCTA	65	32
G-6-P –R	ATCCCAACCACAAGATGACGTTC		
PCK –F	TCTTTGGTGGCCGTAGACCTG	65	30
PCK –R	GCCAGGTATTTGCCGAAGTTGTAG		

2.4.5 免疫荧光

1）将新鲜或冻存的组织按照单位面积细胞密度的大小，选择合适的切片厚度进行切片，若对细胞密度不了解，可查阅文献或通过预实验确定最佳的切片厚度，避免因切片不当影响实验结果；

2）切片风干后立即用4℃预冷的无水甲醇固定10min；

3） 加入5%山羊血清（与二抗来源一致，PBS液稀释），将切片置于有水的湿盒中，于室温封闭1h左右(封闭的目的是防止样本内源性非特异性蛋白抗原的结合)；

4）倾去封闭液也可用干净的吸水纸轻柔吸取，不要过干，保证整个实验过程中组织的湿润，加入一抗稀释液（具体稀释比例按照抗体效价和蛋白表达丰度摸索），不要加太多，最好从组织中间开始滴加至液体刚好覆盖整个组织块即可，此时可形成一圈较厚的覆盖层，否则抗体太多易流走，其结果使组织覆盖的液体很薄，容易干片而无法保证一抗孵育的有效性。加好一抗后将湿盒置于4℃冰箱过夜，次日取出，在室温或37℃恒温箱复温1h左右；

5）轻柔甩去一抗，PBS溶液洗涤3次（5min/次），干净的吸水纸吸去多余的液

万方数据

体，组织依然保持湿润，滴加稀释好的荧光二抗溶液至组织完全覆盖，室温（25℃）反应1h或37℃孵育45min左右；

6）干净的吸水纸吸去二抗不用洗涤，直接滴加DAPI溶液（1μg/mL），在室温条件件避光孵育10min，PBS溶液洗涤3次（5min/次）；

7）加入一滴抗荧光衰减封片剂，轻轻盖上盖玻片，避免多次来回移动盖玻片而导致气泡产生。为防止盖玻片滑动，可在封片后的盖玻片周围平均等距离滴3～4小滴无色指甲油，风干后可起到固定盖玻片的作用；

8）荧光显微镜下观察抓取图片，此切片也可4℃避光保存一周左右。

2.4.6 Western Blot 实验

（1）提取小鼠肝脏组织蛋白：

1） 快速称取0.1g左右在-80℃冰箱冻存的组织，将组织放入培养皿中剪碎，用预冷的0.9%的氯化钠溶液清洗三遍，肝脏组织细胞含血量高，剪碎清洗可去除肝组织大部分血细胞，避免血细胞蛋白影响实验结果。洗完的组织除去多余的液体，转移至玻璃匀浆器，提前加入1mL预冷的RIPA蛋白裂解液和10微升蛋白酶抑制剂（PMSF，1mmol/L，异丙醇配制，避光保存，剧毒），在冰上匀浆（肝组织酥软易碎无需液氮研磨），每半小时补加PMSF一次；

2）将匀浆结束后的组织液转移至1.5mL离心管，于 4℃，12000rpm/min，离心15min；

3）吸取上清液，分装一小部分用于测定蛋白浓度，另一部分保存在超低温冰箱。

（2）蛋白含量测定（BCA法）

测量前，仔细阅读试剂盒配套说明书上的操作步骤和注意事项，按要求配制反应试剂，于酶标仪570nm波长测吸光值。根据标准曲线计算蛋白含量，为了使结果可靠，设置3-5个复孔。

（3）蛋白变性：若蛋白浓度过高，变性前可用RIPA裂解将其浓度稀释至30μg/μL左右，按要求加入蛋白上样缓冲液，混匀离心后于100℃水浴或PCR仪煮沸5min，取出冰浴5min，变性三次后，-80℃冰箱保存。

（4）SDS-PAGE 主要试剂配置：

1）30%丙烯酰胺母液：称1g亚甲双丙烯酰胺和29g丙烯酰胺于烧杯中，加入约60mL 热蒸馏水，搅拌溶解后，定容至 100mL，过滤后4℃避光保存；

2）10%过硫酸铵溶液：每次实验前称取0.005g APS加入500μL蒸馏水溶解；

3）Tris 电泳缓冲液：称甘氨酸14.4g、Tris碱3g、SDS 1g 于烧杯，加入800mL左右蒸馏水溶解后，定容至 1L，可重复使用3次左右；

4）Tris-HCl（pH8.8）：称 18.172g Tris，80mL蒸馏水溶解，用pH计将pH调

万方数据

至 8.8，定容至 100mL；

5）Tris-HCl（pH6.8）：称量 12.118g Tris，用 80mL 蒸馏水溶解，调 pH 至 6.8，定容至 100mL；

6）转膜缓冲液：称量 Gly 5.75g，Tris 1.2g，300mL 蒸馏水溶解后，加入 100mL 甲醇，定容至 500mL，4℃预冷一小时左右，现用现配。

（5）SDS-PAGE

1）准备工作

将制胶所用的玻璃板，梳子，烧杯清洗干净，烘干，制胶前装配好灌胶装置；

2）分离胶配置

按照蛋白分子量选择并配置适宜浓度的分离胶，加好分离胶后（预留好浓缩胶空间）用无水酒精封住液面，室温待其凝固，可根据烧杯中剩余分离胶来判断其是否凝固（半小时左右）；

3）配置浓缩胶

分离胶凝好后，配制浓缩胶，先不要加 APS 和 TEMED，分离胶凝固后倾去上面的酒精液面，用蒸馏水冲洗 4 至 5 次后，用干净的滤纸吸去多余的水分，不要触碰胶面，所有操作都要注意保护好分离胶自身的完整性，最后加入 APS 和 TEMED 快速搅匀浓缩胶溶液后，将其加在分离胶上，立即插好梳子，避免产生气泡，室温待凝；

4）上样

安装好电泳装置，电泳槽倒好电泳液，提前将冻存的蛋白取出，室温融解后，上样前 90℃，煮沸 3min，冰浴 2min，离心后再上样。调内参前，在 20-40μg 之间选一蛋白质量计算各样本上样体积，检测目的蛋白相对表达时，按照内参条带一致时的上样体积加样；

5）电泳

设置电泳电压和时间，浓缩胶 70V，分离胶 110V，待接近目的蛋白分子量的 Marker 条带分离时停止跑胶。

（6）转膜

1）可在电泳快结束前，裁好 PVDF 膜（左上角剪去一角，以示正面），用无水甲醇活化 15s，蒸馏水浸泡 2min，转移至含转膜缓冲液的培养皿中平衡 5min，转膜所用的滤纸也应在含转膜缓冲液的容器中平衡 5min；

2）半干转膜时，上下三层滤纸的中间分别是 PVDF 膜和含目的蛋白的分离胶，膜在下，胶在上。恒流转膜，具体转膜电流，时间根据 PVDF 膜说明书和蛋白分子量来确定。

（7）免疫杂交反应：

1）转膜结束，取出 PVDF 膜，于 5%的牛奶在摇床上（60 rpm/min）室温封闭 1h；

万方数据

2）封闭后，将PVDF膜放在含一抗稀释液的离心管中，要保证膜始终浸泡在液体中，置于加入碎冰的泡沫盒（将碎冰压实，否则冰块融化，表面塌陷，引起液面倾斜，导致一抗孵育不均匀），摇床上（60 rpm/min）孵育过夜，若目的蛋白表达丰度低或抗体效价不高时，可适当提高抗体稀释比例或额外在室温孵育1h左右；

3）将PVDF膜取出放在含TBST的培养皿中，于摇床（设置摇床转速为80 rpm/min）上洗涤3次（10min/次）；

4）荧光二抗按照说明书推荐的稀释比例配制，将洗完一抗的膜转移至含二抗稀释液的离心管中室温避光孵育1h；

5）将PVDF膜取出放在含TBST的培养皿中，于摇床（设置摇床转速为80 rpm/min）上避光洗涤3次（10min/次）；

6）使用Odyssey 双色红外荧光成像系统扫描PVDF膜，显影；

7）数据分析：

使用Image J 软件对条带进行灰度值测定。

2.4.7 数据统计分析

将分析所得灰度值多组间比较采用SPSS21.0进行统计学意义分析，两组间比较采用 t 检验分析。最后根据灰度平均值、标准差以及显著性 *P* 值作图，所有数据均以均数 ± 标准差（$\bar{x}$±SD）表示，$P<0.05$，认为有显著性差异。

万方数据

第三章　结　　果

3.1 1 型糖尿病小鼠的建立

腹腔注射 STZ 的模型组小鼠与 OVX 对照组相比，体重增长缓慢，且在术后第四周、第五周和第六周末体重明显低于 OVX 组（Fig. 1A）；血糖在术后第二周至第六周末（也就是造模第一周至第五周末）显著升高（P<0.001）（Fig. 1 B）；胰岛素抵抗实验结果并无显著性差异（Fig. 2）。

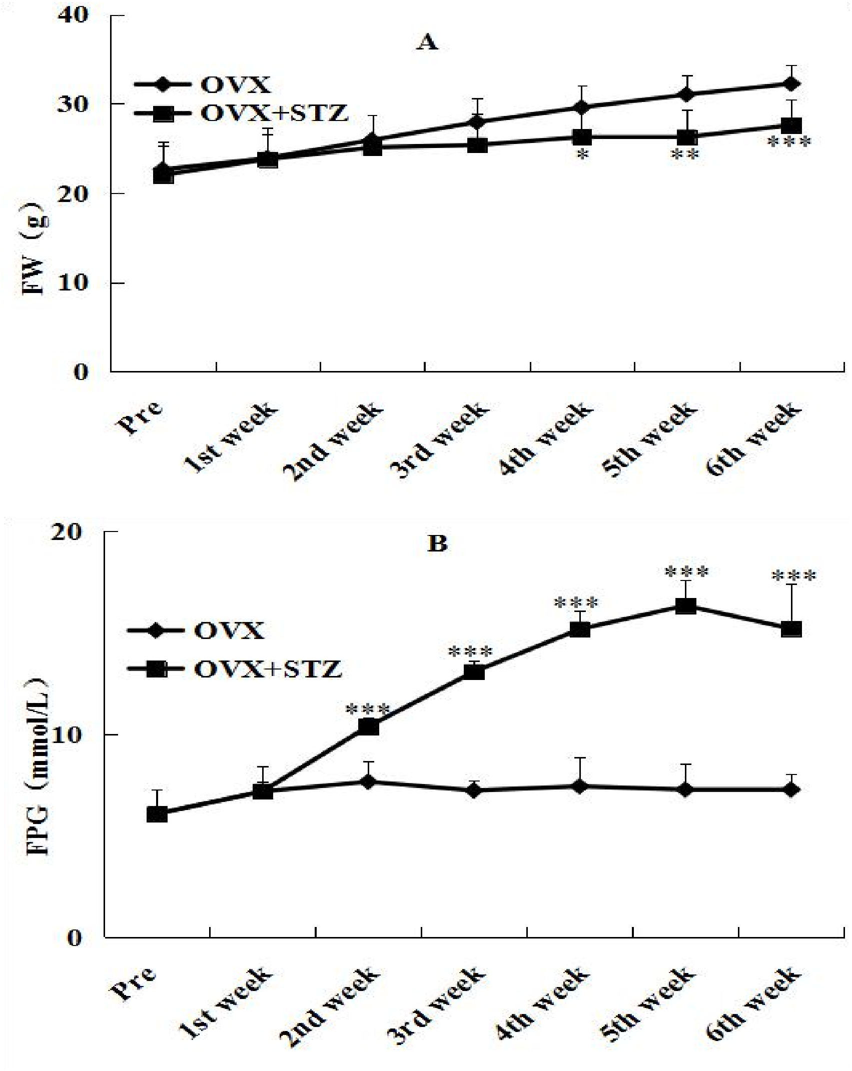

Fig. 1 The fasting weight (A) and fasting plasma glucose (B) of type 1 diabetes mice

图 1　1 型糖尿病小鼠空腹体重和血糖

Fasting weight (FW) and Fasting plasma glucose (FPG) in ovariectomized mice (OVX) and in ovariectomized and streptozotocin-induced diabetes mice (OVX+STZ). The results represent means±SD (n=12). $^{*}P$<0.05, $^{**}P$<0.01, $^{***}P$<0.001 versus OVX.

万方数据

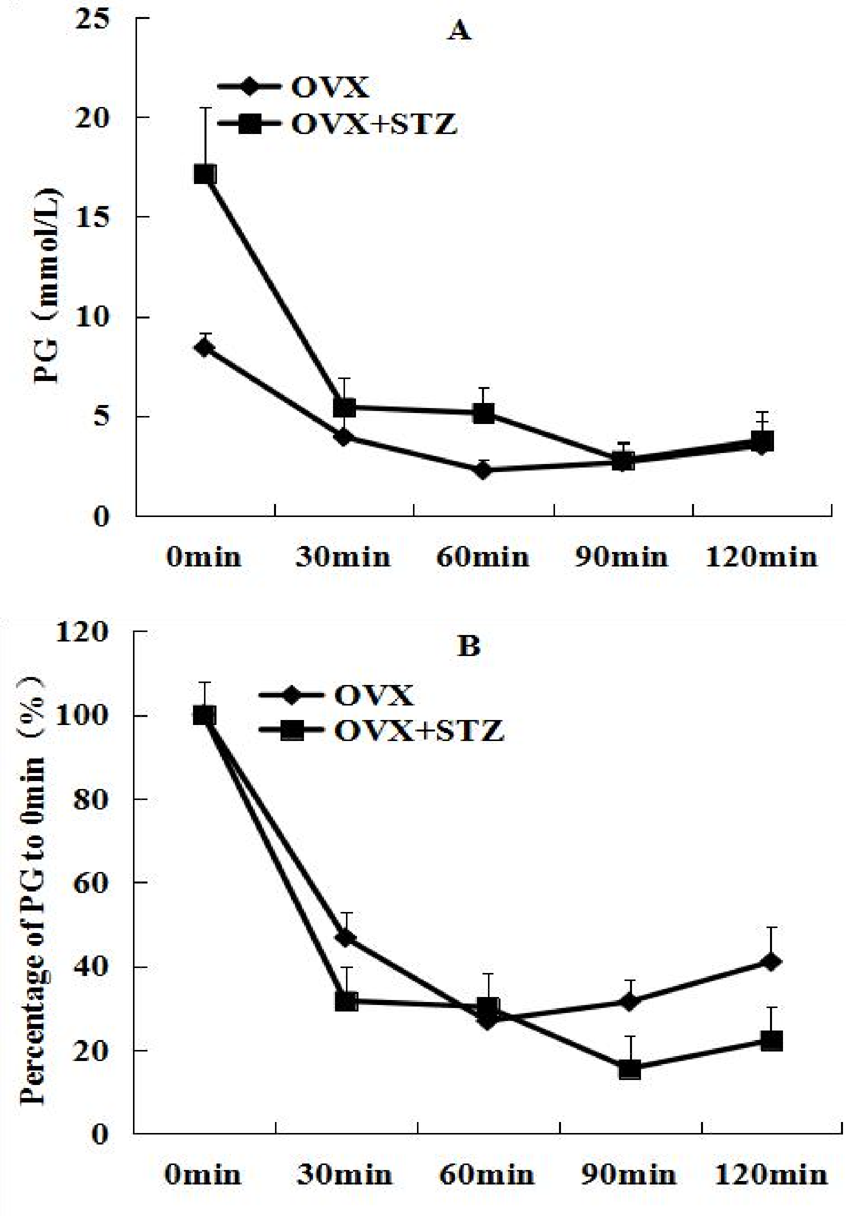

Fig. 2 The insulin resistance experiment of type 1 diabetes mice

图 2 1 型糖尿病小鼠胰岛素抵抗实验

Insulin resistance test in ovariectomized mice (OVX) and in ovariectomized streptozotocin-induced diabetes mice (OVX+STZ). The plasma glucose (PG) changes after insulin injection (A), Percentage change in plasma glucose at different time points after insulin injection relative to the plasma glucose level before administrtion insulin (B). The results represent means±SD (n=6).

万方数据

3.2 17β-雌二醇对糖尿病小鼠空腹体重和血糖的影响

如 Fig. 3 所示，与模型组 OVX+STZ 相比，三种剂量（0.01、0.1 和 1mg/kg 体重）17β-雌二醇使 STZ 所致糖尿病小鼠体重有升高趋势（$P>0.05$）（Fig. 3A）；其中 0.1 和 1mg/kg 体重的雌二醇给药时，小鼠在术后第二周末（也就是给药第一周末）空腹血糖明显下降（$P<0.01$），在术后第三周、第四周、第五周末（也就是给药第二周、第三周和第四周末）血糖下降更为显著（$P<0.001$）（Fig. 3B）。

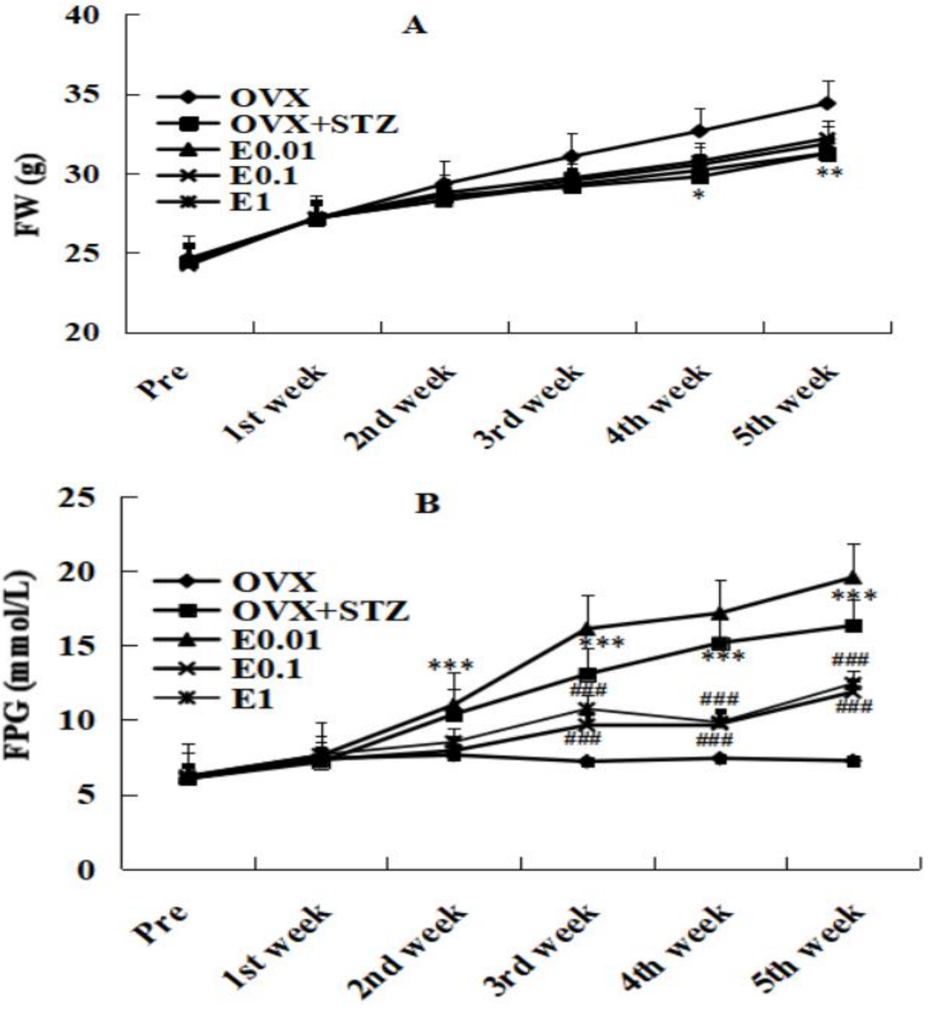

Fig. 3　Effects of 17β-estradiol on fasting body weight (A) and plasma glucose (B) in diabetic mice

图 3　17β-雌二醇对糖尿病小鼠空腹体重和血糖的影响

Fasting weight (FW) and Fasting plasma glucose (FPG) in ovariectomized mice (OVX), in ovariectomized and streptozotocin-induced diabetes mice (OVX+STZ), in ovariectomized and streptozotocin-induced diabetes mice treated with 17β-estradiol at the dose of 0.01mg/kg body weight (E0.01), 0.1mg/kg body weight (E0.1) and 1mg/kg body weight (E1) . The results represent means±SD. (n=12). $^{**}P<0.01$, $^{***}P<0.001$ versus OVX; $^{\#\#}P<0.01$, $^{\#\#\#}P<0.001$ versus OVX+STZ.

万方数据

3.3 黄体酮对糖尿病小鼠空腹体重和血糖的影响

与模型组相比，黄体酮给药组（0.1、2 和 8mg/kg 体重）体重增加均快于模型组，黄体酮（2mg/kg 体重）对 1 型糖尿病小鼠体重的影响在术后第五周和第六周末（也就是给药第四周和第五周末）具有显著性差异（*P*<0.05）（Fig. 4A）；黄体酮给药组血糖在术后第四周后（也就是给药第三周后）均低于模型组，但 2mg/kg 体重黄体酮给药组在术后第四周、第五周和第六周末（也就是给药第三周、第四周和第五周末）血糖下降具有显著性差异（*P*<0.001）（Fig. 4B）。

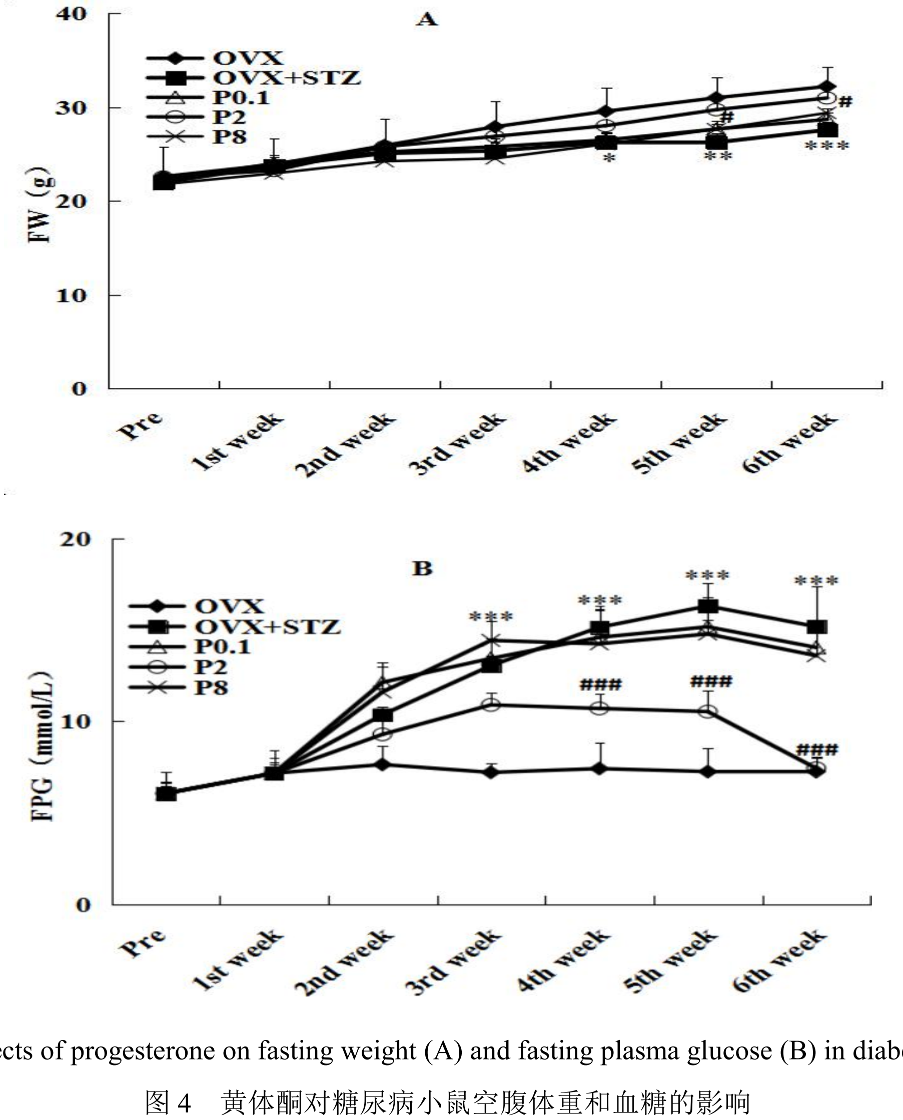

Fig. 4 Effects of progesterone on fasting weight (A) and fasting plasma glucose (B) in diabetic mice

图 4 黄体酮对糖尿病小鼠空腹体重和血糖的影响

Fasting weight (FW) and Fasting plasma glucose (FPG) in ovariectomized mice (OVX), in ovariectomized and streptozotocin-induced diabetes mice (OVX+STZ), in ovariectomized and streptozotocin-induced diabetes mice respectively treated with progesterone at the dose of 0.1mg/kg body weight (P0.1), 2mg/kg body weight (P2) or 8mg/kg body weight (P8). The results represent means±SD. (n=12). $^{*}P<0.05$, $^{**}P<0.01$, $^{***}P<0.001$ versus OVX; $^{\#}P<0.05$, $^{\#\#}P<0.01$, $^{\#\#\#}P<0.001$ versus OVX+STZ.

万方数据

3.4 17β-雌二醇和黄体酮联用对体重和血糖的影响

与模型组（OVX+STZ）相比，17β-雌二醇（0.1mg/kg 体重）单独给药使体重变化有升高趋势；17β-雌二醇和黄体酮联合组（EP0.1、EP2、EP8）体重增长也较快，但与 17β-雌二醇单独给药组相比，联用时体重变化并无显著性差异（Fig. 5A）；17β-雌二醇（0.1mg/kg 体重）单独给药使糖尿病小鼠的血糖在术后第三周、第四周、第五周和第六周末（也就是给药第二周、第三周、第四周和第五周末）显著下降（P<0.001）；17β-雌二醇和黄体酮联合组（EP0.1、EP2、EP8）血糖相对模型组（OVX+STZ）始终处于较低水平，且 EP0.1 和 EP2 组与 E0.1 组相比在术后第五和第六周末（也就是给药第四和第五周末）具有显著性差异（P<0.01）（Fig. 5B）。

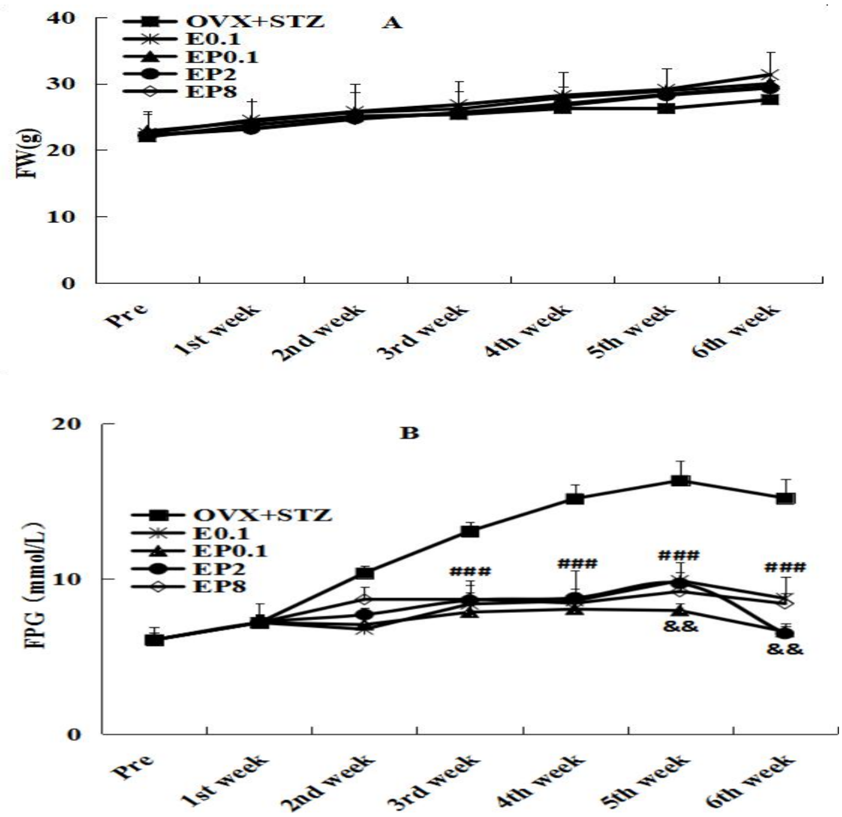

Fig. 5 Effects of 17β-estradiol and progesterone combination on fasting weight (A) and fasting plasma glucose (B) in diabetic mice

图 5 17β-雌二醇和黄体酮联用对 STZ 所致糖尿病小鼠空腹体重和血糖的影响

Fasting weight (FW) and Fasting plasma glucose (FPG) in ovariectomized mice (OVX), in ovariectomized and streptozotocin-induced diabetes mice (OVX+STZ), in ovariectomized and streptozotocin-induced diabetes mice treated with 0.1mg/kg body weight 17β-estradiol (E0.1), or treated with the combination of 0.1mg/kg body weight 17β-estradiol and 0.1mg/kg body weight progesterone (EP0.1), 2mg/kg body weight progesterone (EP2), or 8mg/kg body weight progesterone (EP8). The results represent means±SD. (n=6). $^{\#}P$<0.05, $^{\#\#\#}P$<0.001 versus OVX+STZ; $^{\&\&}P$<0.01 versus E0.1.

万方数据

3.5 17β-雌二醇和黄体酮对糖尿病小鼠糖耐量的影响

如图 Fig. 6 所示，给药干预五周后，禁食不禁水 12h，进行葡萄糖耐量实验，腹腔注射葡萄糖（1.5g/kg 体重），分别在注射葡萄糖后的 0min、30min、60min、90min、120min 测血糖，模型组与对照组相比，糖耐量受损且具有显著性差异（P<0.001），三种剂量的黄体酮对 1 型糖尿病小鼠的糖耐量损伤无明显改善；而 0.1mg/kg 体重 17β-雌二醇及 17β-雌二醇和黄体酮联用，其中 EP2 联用组糖耐量损伤有一定改善改善，在注射葡萄糖后的 90min 达到显著水平（P<0.05）。

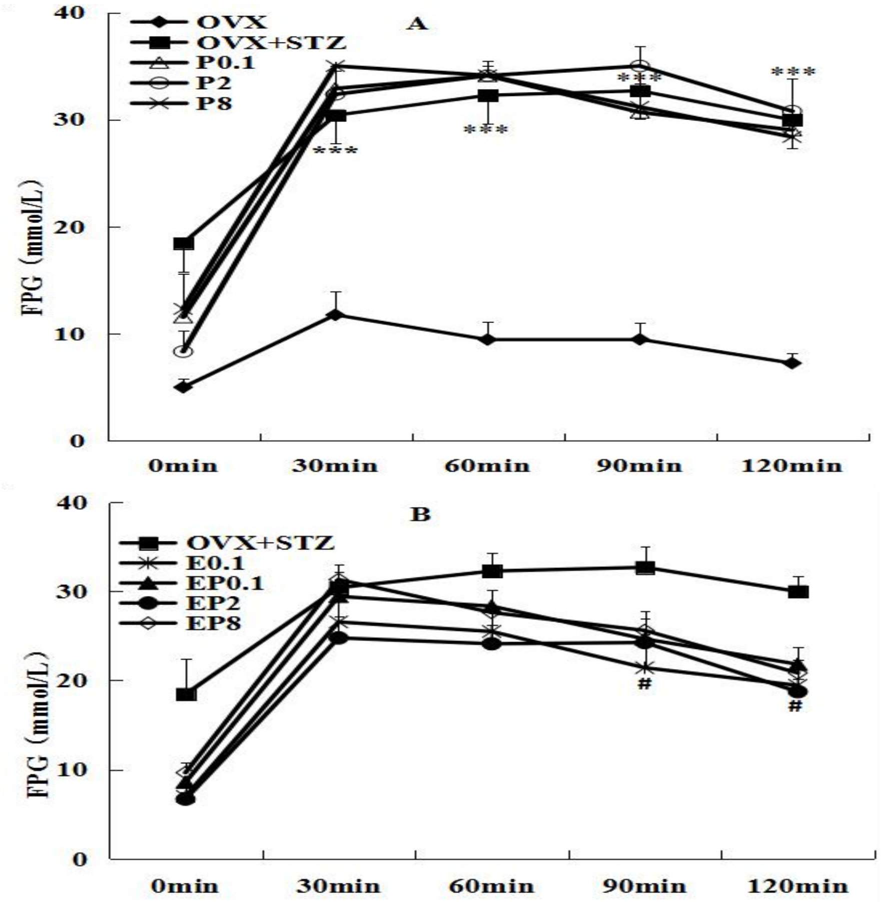

Fig. 6 Effects of 17β-Estradiol and progesterone on glucose tolerance in diabetic mice

图 6 17β-雌二醇和黄体酮对糖尿病小鼠糖耐量的影响

Glucose tolerance test (GTT) in ovariectomized mice (OVX), in ovariectomized and streptozotocin-induced diabetes mice (OVX+STZ), in ovariectomized and streptozotocin-induced diabetes mice treated with 0.1mg/kg body weight progesterone (P0.1), 2mg/kg body weight progesterone (P2), 8mg/kg body weight progesterone (P8), 0.1mg/kg body weight 17β-estradiol (E0.1) or treated with the combination of 0.1mg/kg body weight 17β-estradiol and 0.1mg/kg body weght progesterone (EP0.1), 2mg/kg body weight progesterone (EP2) or 8mg/kg body weight progesterone (EP8). The results represent means±SD. (n=6). $^{***}P$<0.001 versus OVX; $^{\#}P$<0.05, $^{\#\#}P$<0.01, $^{\#\#\#}P$<0.001 versus OVX+STZ.

万方数据

3.6 17β-雌二醇和黄体酮对糖尿病小鼠胰岛的影响

胰岛苏木精—伊红染色（Hematoxylin-eosin staining, HE）结果如 Fig. 7 所示，模型组胰岛细胞和对照组相比，形态不规则，萎缩严重，胰岛细胞数量减少，而给药组胰岛形态变化趋向于规整，胰岛细胞数量有所增加（Fig. 8）。

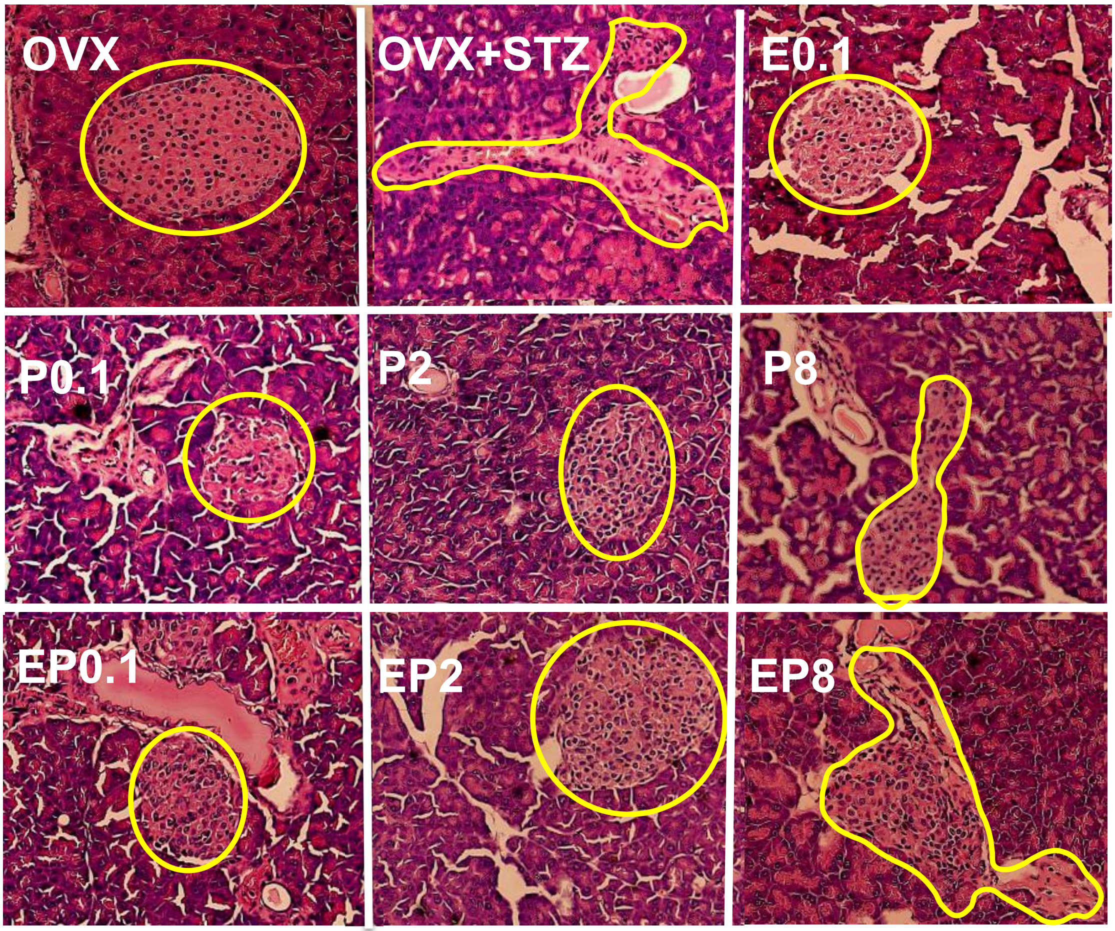

Fig. 7 The picture of Islets morphology

图 7 胰岛形态图

Effects of 17β-estradiol and progesterone on pancreas were visualized by hematoxylin-eosin (HE) staining in ovariectomized mice (OVX), in ovariectomized and streptozotocin-induced diabetes mice (OVX+STZ), in ovariectomized and streptozotocin-induced diabetes mice treated with 0.1mg/kg body weight 17β-estradiol (E0.1), 0.1mg/kg body weight progesterone (P0.1), 2mg/kg body weight progesterone (P2), 8mg/kg body weight progesterone (P8), or treated with the combination 0.1mg/kg body weight 17β-estradiol and 0.1mg/kg body weight progesterone (EP0.1), 2mg/kg body weight progesterone (EP2), or 8mg/kg body weight progesterone (EP8) (n=3).

万方数据

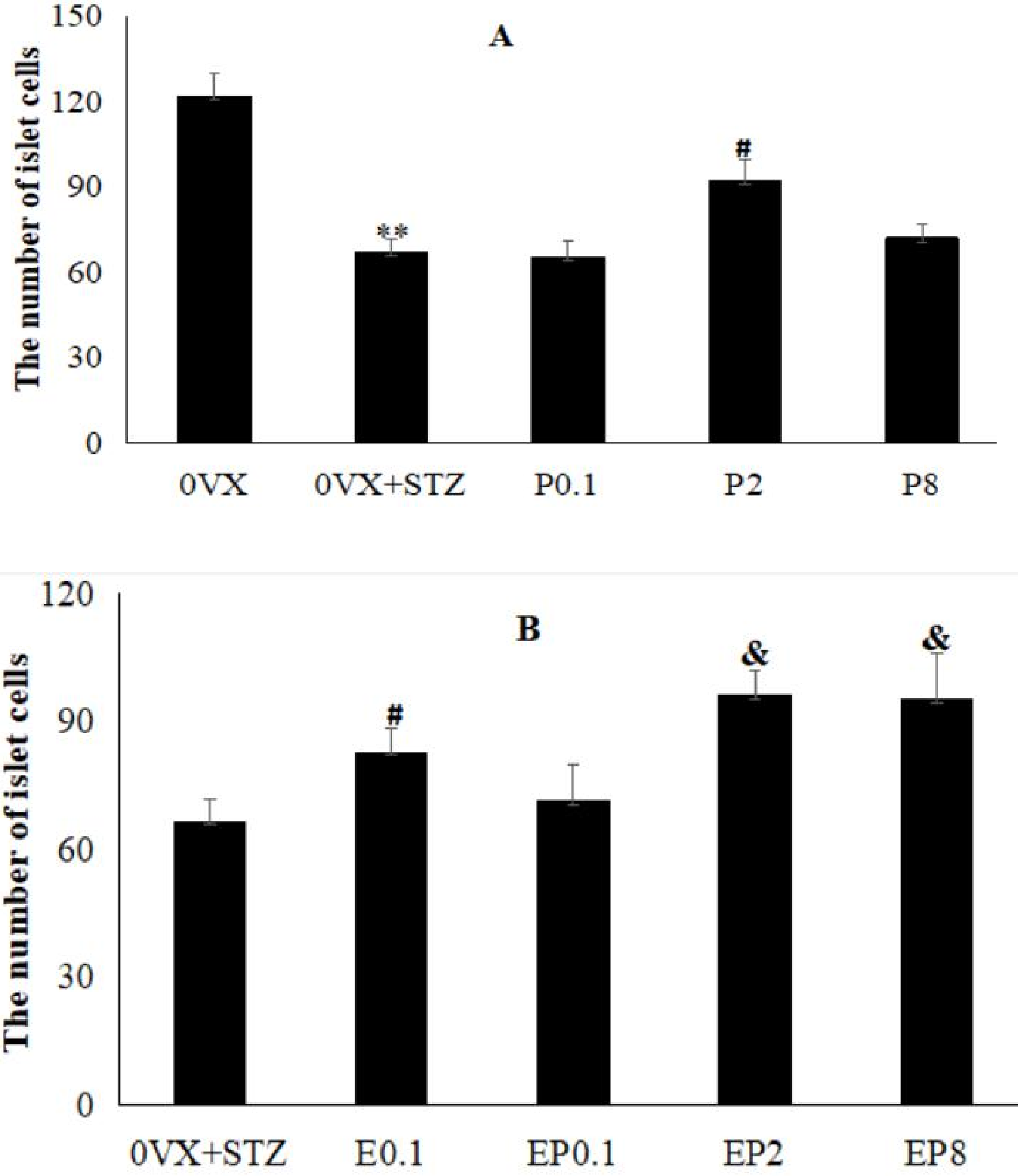

Fig. 8　The number of islet cells

图 8　胰岛细胞数量

Effects of 17β-estradiol and progesterone on pancrea islet cell numbers, the number of islet cells were counted by hematoxylin-eosin (HE) staining in ovariectomized mice (OVX), in ovariectomized and streptozotocin-induced diabetes mice (OVX+STZ), in ovariectomized and streptozotocin-induced diabetes mice treated with 0.1mg/kg body weight progesterone (P0.1), 2mg/kg body weight progesterone (P2), 8mg/kg body weight progesterone (P8), 0.1mg/kg body weight 17β-estradiol (E0.1) or treated with the combination of 0.1mg/kg body weight 17β-estradiol and 0.1mg/kg body weight progesterone (EP0.1), 2mg/kg body weight progesterone (EP2), or 8mg/kg body weight progesterone (EP8)(n=3). The results represent means±SD. (n=6). $^{**}P<0.01$ versus OVX; $^{\#}P<0.05$ versus OVX+STZ. $^{\&}P<0.05$ versus E0.1.

万方数据

3.7 血清性激素及 C 肽和胰岛素水平检测

给药 5 周后，眼球采血收集小鼠血清、化学发光法检测血清中 17β-雌二醇和黄体酮的含量，Elisa 法检测血清中 C 肽和胰岛素水平。从 Table 1 和 Table 2 中可以看出模型组小鼠 C 肽和胰岛素含量均比对照组显著减少；而 0.1mg/kg 体重 17β-雌二醇和 2mg/kg 体重黄体酮单独给药，血清 C 肽和胰岛素水平明显提高；17β-雌二醇和黄体酮联用，血清 C 肽和胰岛素含量均有所增加，但低剂量（0.1mg/kg 体重）黄体酮有协同作用，大剂量（8mg/kg 体重）有拮抗作用（Table 2）。

Table 1 Plasma concentrations of sex hormone C-Pepitide and insulin

表 1 不同组小鼠血清性激素及 C 肽和胰岛素水平

Groups	Estrodiol(pg.mL^{-1})	Progesterone(ng.mL^{-1})	C-peptide (ng.mL^{-1})	Insulin(mIU.mL^{-1})
OVX	86.77±13.3	0.37±0.3	5.75±1.1	29.56±2.3
OVX+STZ	84.49±12.2	0.37±0.3	3.63±0.8*	19.48±2.2 ***
P0.1	84.09±11.3	0.57±0.1	4.22±0.2	20.13±0.3
P2	88.5±22.2	1.77±1.3	5.31±0.3#	25.95±6.3 #
P8	81.1±8.1	7.78±4.8	4.65±0.2	22.24±5.2

Plasma concentrations of 17β-estradiol, progesterone, C-Pepitide and insulin in ovariectomized mice (OVX), in ovariectomized and streptozotocin-induced diabetes mice (OVX+STZ), in ovariectomized and streptozotocin-induced diabetes mice treated with 0.1mg/kg body weight progesterone (P0.1), 2mg/kg body weight progesterone (P2), 8mg/kg body weight progesterone (P8). The results represent means±SD. (n=6). $^{*}P<0.05$, $^{***}P<0.001$ versus OVX; $^{\#}P<0.05$ versus OVX+STZ.

Table 2 Plasma concentrations of sex hormone C-Pepitide and insulin

表 2 17β-雌二醇和黄体酮联用组小鼠血清性激素及 C 肽和胰岛素水平

Groups	Estrodiol(pg.mL^{-1})	Progesterone(ng.mL^{-1})	C-peptide (ng.mL^{-1})	Insulin(mIU.mL^{-1})
OVX+STZ	84.49±12.2	0.37±0.3	3.63±0.8	19.48±2.2
E0.1	186.53±66.1	0.34±0.3	5.99±1 #	31.71±2.7###
EP0.1	128.85±6.1	0.65±0	19.28±1.5&&&	78.22±8.2&&&
EP2	210.28±99.6	1.77±1.3	5.12±0.4	27.81±9.8
EP8	221.62±77.5	7.78±4.8	4.78±0.1&	23.6±2.1&&&

Plasma concentrations of 17β-estradiol, progesterone, C-Pepitide and insulin in ovariectomized mice （OVX), in ovariectomized and streptozotocin-induced diabetes mice (OVX+STZ), in ovariectomized and streptozotocin-induced diabetes mice treated with 0.1mg/kg body weight 17β-estradiol (E0.1), or treated with the combination of 0.1mg/kg body weight 17β-estradiol and 0.1mg/kg body weight progesterone (EP0.1), 2mg/kg body weight progesterone (EP2), or 8mg/kg body weight progesterone (EP8). The results represent means±SD. (n=6). $^{\#}P<0.05$, $^{\#\#\#}P<0.001$versus OVX+STZ; $^{\&}P<0.05$, $^{\&\&\&}P<0.001$ versus E0.1.

万方数据

3.8 17β-雌二醇和黄体酮对糖尿病小鼠胰岛素受体表达的影响

如 Fig. 9 所示，肝脏胰岛素受体 mRNA（Fig. 9 A 和 B）和蛋白（Fig. 9 C 和 D）表达在模型组（OVX+STZ）、对照组（OVX）以及给药组之间无显著性差异。如 Fig. 10 所示，免疫荧光检测显示肝脏和骨骼肌胰岛素受体表达在模型组（OVX+STZ）与对照组（OVX）之间也无明显差异。

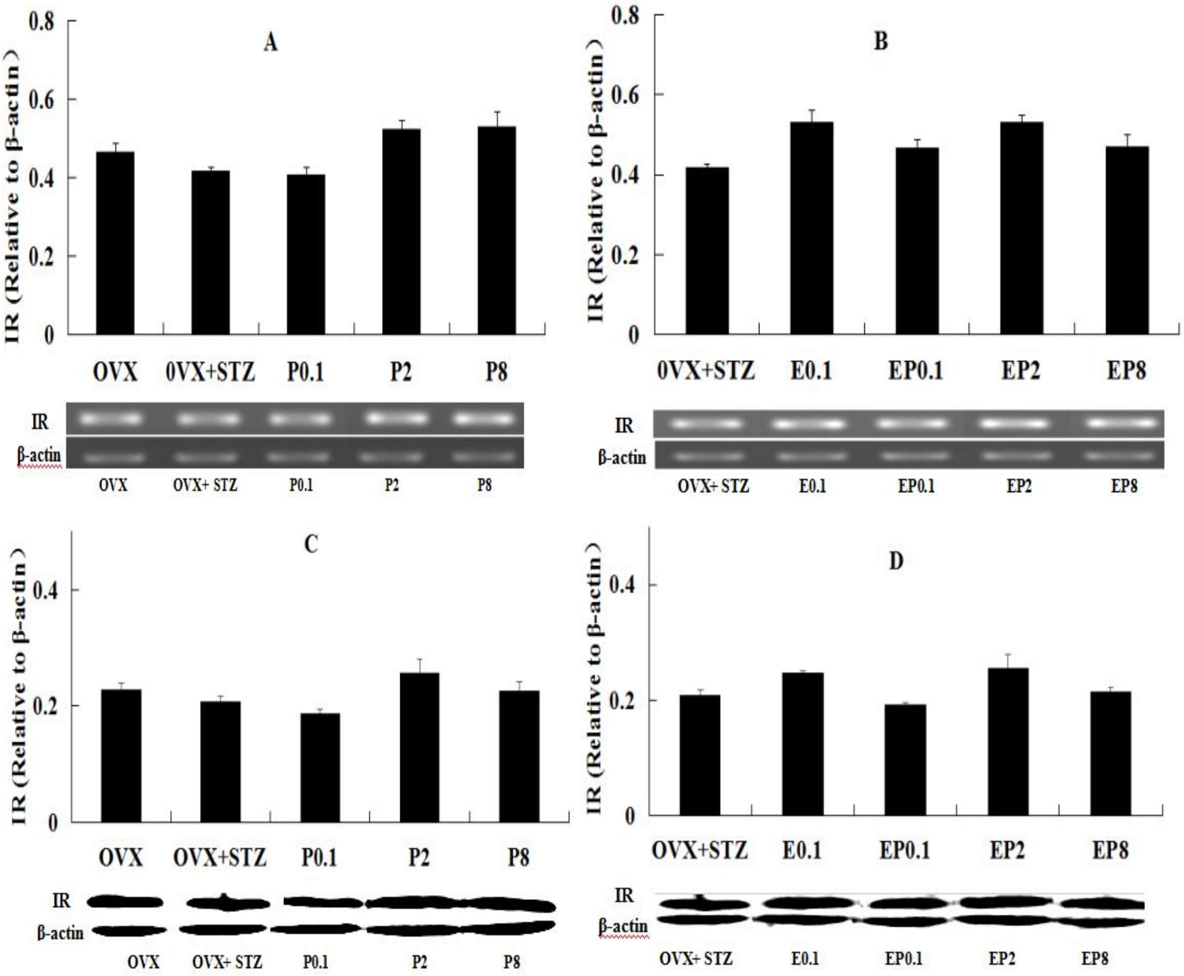

Fig. 9 The expressions of liver insulin receptor

图 9 肝脏胰岛素受体表达

Effects17β-estradiol and progesterone on liver insulin receptor mRNA (A, B) and protein (C, D) expressions in ovariectomized mice (OVX), in ovariectomized and streptozotocin-induced diabetes mice (OVX+STZ), in ovariectomized and streptozotocin-induced diabetes mice treated with 0.1mg/kg body weight progesterone (P0.1), 2mg/kg body weight progesterone (P2), 8mg/kg body weight progesterone (P8), 0.1mg/kg body weight 17β-estradiol (E0.1) or treated with the combination of 0.1mg/kg body weight 17β-estradiol and 0.1mg/kg body weight progesterone (EP0.1), 2mg/kg body weight progesterone (EP2) or 8mg/kg body weight progesterone (EP8). The results represent means±SD. (n=3).

万方数据

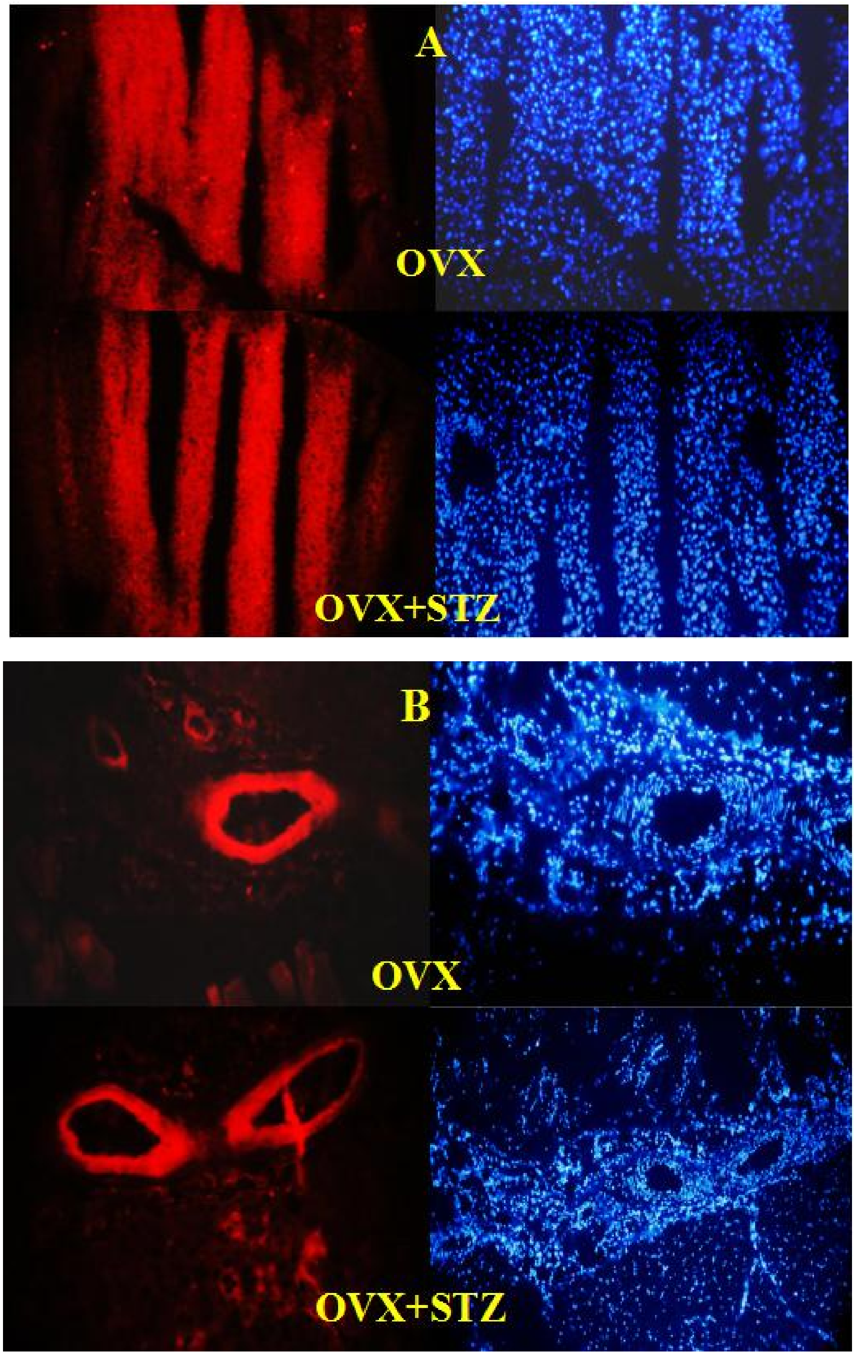

Fig. 10 Immunofluorescence detection of insulin receptor expression in liver and seletal muscle

图 10 免疫荧光法检测肝脏和骨骼肌胰岛素受体表达

Liver (A) and skeletal muscle (B) insulin receptor expressions in ovariectomized (OVX) and in ovariectomized and streptozotocin-induced diabetes mice (OVX+STZ) were visualized by immunofluorescent staining using anti-insulin receptor (fluorescein, red) antibody and DAPI staining for visualizing nuclei (blue) (n=3).

万方数据

3.9 17β-雌二醇和黄体酮对 GLUT mRNA 表达的影响

3.9.1 17β-雌二醇和黄体酮对骨骼肌 GLUT4 mRNA 表达的影响

如 Fig. 11 所示，模型组（OVX+STZ）骨骼肌 GLUT4 mRNA 表达相比对照组（OVX）显著降低（*P*<0.001），17β-雌二醇和黄体酮单独给药可以上调模型组骨骼肌 GLUT4 mRNA 的表达，P0.1 和 P2 组上调明显，E0.1 组上调作用具有显著性（*P*<0.001）；0.1mg/kg 体重 17β-雌二醇与 2mg/kg 体重黄体酮可显著增强 17β-雌二醇的上调作用（*P*<0.001），具有协同作用，但 0.1 和 8mg/kg 体重黄体酮对 17β-雌二醇的作用具有拮抗效应。

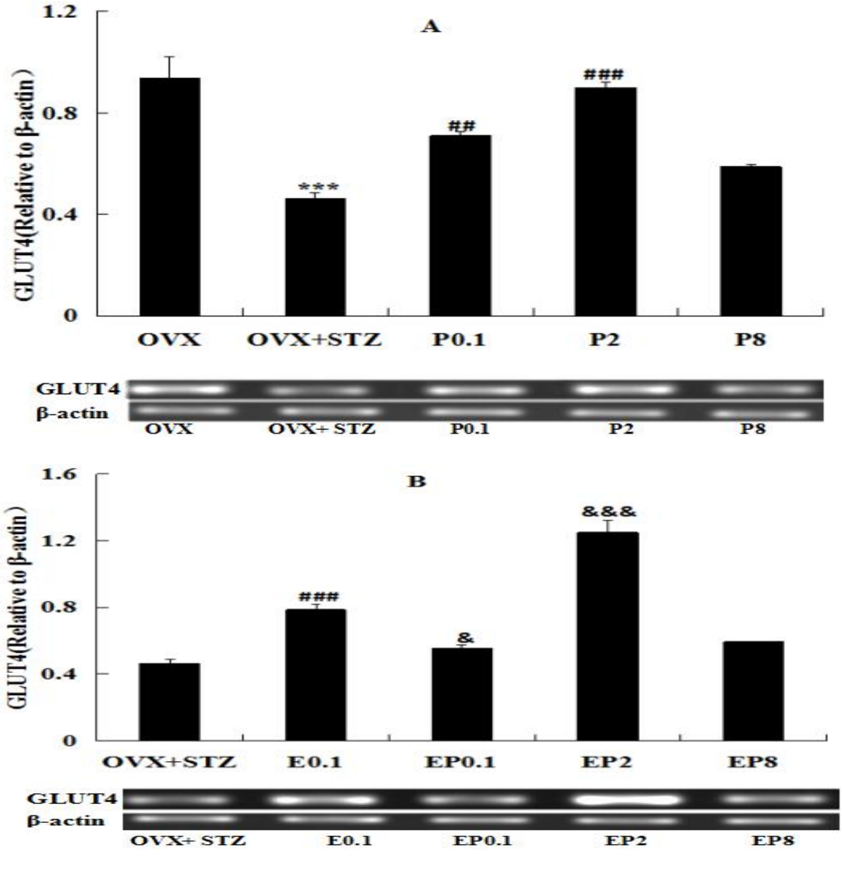

Fig. 11 Effects of 17β-estradiol and progesterone on GLUT4 mRNA expression in skeletal muscle

图 11 17β-雌二醇和黄体酮对骨骼肌 GLUT4 mRNA 表达的影响

The GLUT4 mRNA expressions of skeletal muscle in ovariectomized mice (OVX), in ovariectomized and streptozotocin-induced diabetes mice (OVX+STZ), in ovariectomized and streptozotocin-induced diabetes mice treated with 0.1mg/kg body weight progesterone (P0.1), 2mg/kg body weight progesterone (P2), 8mg/kg body weight progesterone (P8), 0.1mg/kg body weight 17β-estradiol (E0.1) or treated with the combination of 0.1mg/kg body weight 17β-estradiol and 0.1mg/kg body weight progesterone (EP0.1), 2mg/kg body weight progesterone (EP2) or 8mg/kg body weight progesterone (EP8). The results represent means±SD. (n=3). $^{***}P<0.001$ versus OVX; $^{\#\#}P<0.01$, $^{\#\#\#}P<0.001$ versus OVX+STZ; $^{\&}P<0.05$, $^{\&\&\&}P<0.001$ versus E0.1.

万方数据

3.9.2 17β-雌二醇和黄体酮对肝脏 GLUT2 mRNA 表达的影响

如 Fig. 12 所示，模型组（OVX+STZ）肝脏 GLUT2 mRNA 表达相比对照组（OVX）明显增加（$P<0.001$）；17β-雌二醇和黄体酮单独给药可以下调模型组肝脏 GLUT2 mRNA 的表达，P2、P8 和 E0.1 组下调作用明显（$P<0.001$）；17β-雌二醇和黄体酮联用时，同样下调其表达，但与单独 17β-雌二醇给药组相比无显著性差异。

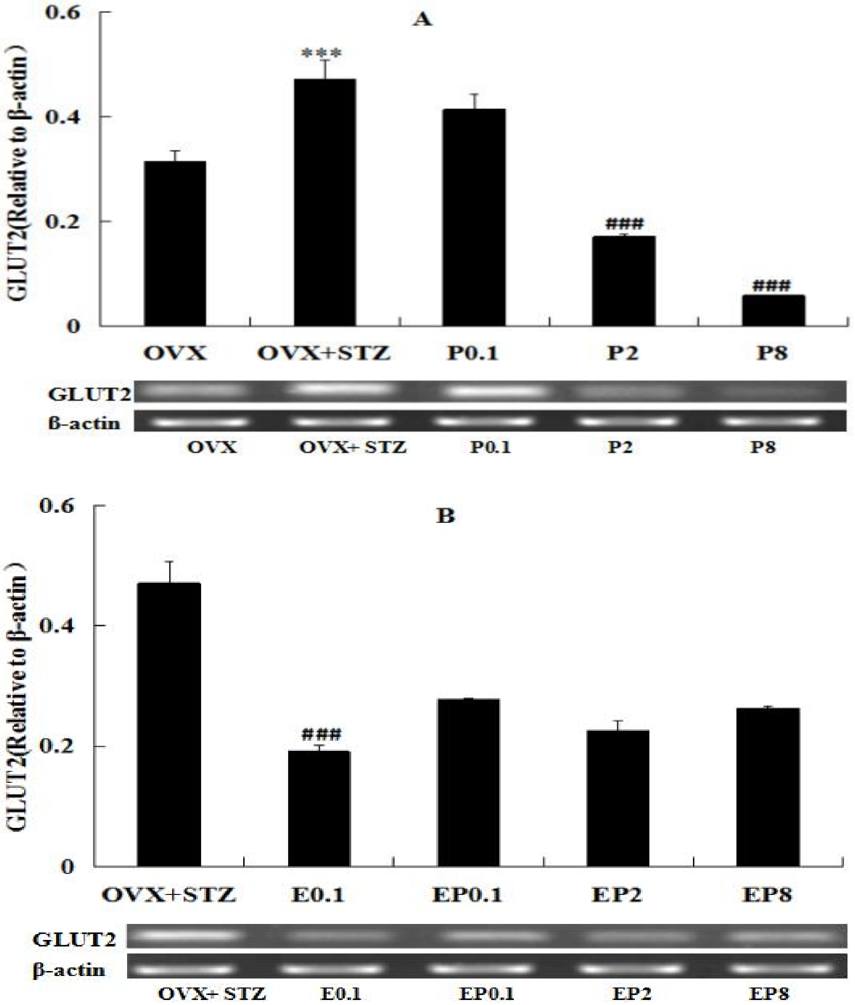

Fig. 12 The expressions of liver GLUT2 mRNA

图 12 肝脏 GLUT2 mRNA 表达

The liver GLUT2 mRNA expression in ovariectomized mice (OVX), in ovariectomized and streptozotocin-induced diabetes mice (OVX+STZ), in ovariectomized and streptozotocin-induced diabetes mice treated with 0.1mg/kg body weight progesterone (P0.1), 2mg/kg body weight progesterone (P2), 8mg/kg body weight progesterone (P8), 0.1mg/kg body weight 17β-estradiol (E0.1) or treated with the combination of 0.1mg/kg body weight 17β-estradiol and 0.1mg/kg body weight progesterone (EP0.1), 2mg/kg body weight progesterone (EP2) or 8mg/kg body weight progesterone (EP8). The results represent means±SD. (n=3). $^{***}P<0.001$ versus OVX; $^{\#\#\#}P<0.001$ versus OVX+STZ.

万方数据

3.10 17β-雌二醇和黄体酮对肝脏糖代谢酶表达的影响

3.10.1 17β-雌二醇和黄体酮对肝脏 Gck mRNA 表达的影响

如 Fig. 13 所示，模型组肝脏 Gck mRNA 表达相比 OVX 组显著减少（$P<0.001$）；17β-雌二醇和黄体酮单独给药组可以上调肝脏 Gck mRNA 表达，0.1 和 2mg/kg 体重黄体酮及 0.1mg/kg 体重 17β-雌二醇上调作用显著（$P<0.001$）；17β-雌二醇和黄体酮联用组同样上调 Gck mRNA 表达，但低剂量（0.1mg/kg 体重）和大剂量（8mg/kg 体重）黄体酮与 17β-雌二醇（0.1mg/kg 体重）联用可明显拮抗 17β-雌二醇的上调作用（Fig. 13B）。

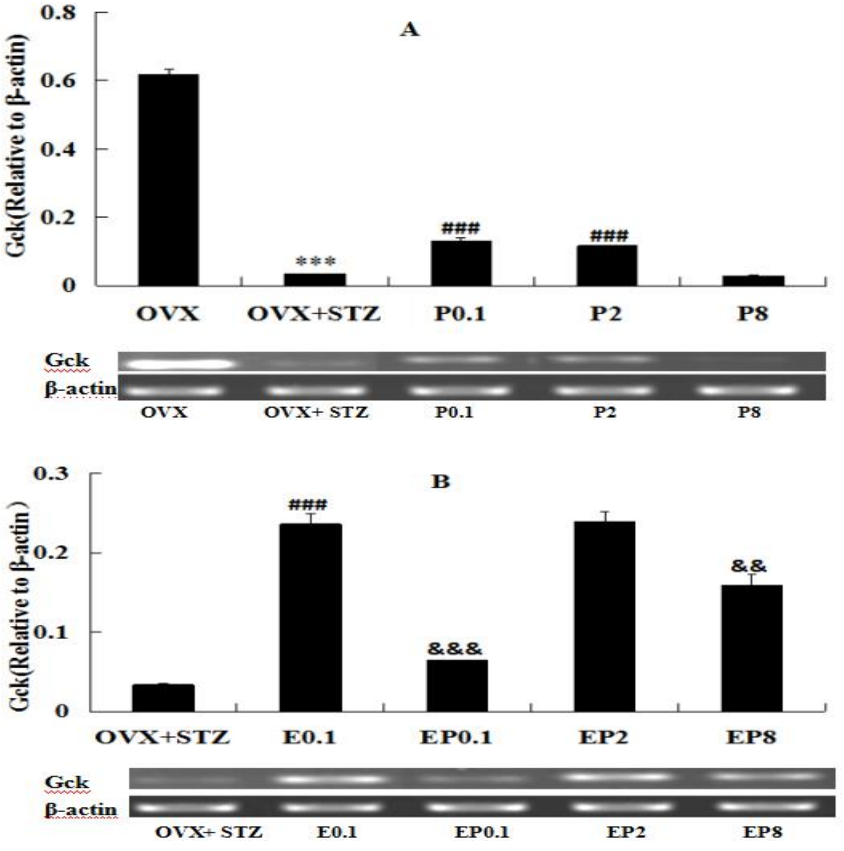

Fig. 13 The expressions of liver Gck mRNA

图 13　肝脏 Gck mRNA 表达

The effects of progesterone and 17β-estradiol on liver Gck mRNA expression in ovariectomized mice (OVX), in ovariectomized and streptozotocin-induced diabetes mice (OVX+STZ), in ovariectomized and streptozotocin-induced diabetes mice treated with 0.1mg/kg body weight progesterone (P0.1), 2mg/kg body weight progesterone (P2), 8mg/kg body weight progesterone (P8), 0.1mg/kg body weight 17β-estradiol (E0.1) or treated with the combination of 0.1mg/kg body weight 17β-estradiol and 0.1mg/kg body weight progesterone (EP0.1), 2mg/kg body weight progesterone (EP2) or 8mg/kg body weight progesterone (EP8). The results represent means±SD. (n=3). $^{***}P<0.001$ versus OVX; $^{\#\#\#}P<0.001$ versus OVX+STZ; $^{\&\&}P<0.01$, $^{\&\&\&}P<0.001$ versus E0.1.

万方数据

3.10.2 17β-雌二醇和黄体酮对肝脏 G-6-P 和 PCK mRNA 表达的影响

如 Fig. 14 所示，模型组（OVX+STZ）肝脏 G-6-P 和 PCK mRNA 表达相比对照组（OVX）明显提高（*P*<0.05）；17β-雌二醇和黄体酮单独或联合给药时均可显著下调肝脏 G-6-P mRNA 表达（*P*<0.001），也可下调 PCK mRNA 表达，但不同剂量的黄体酮与 17β-雌二醇联用，对 17β-雌二醇下调 G-6-PmRNA（Fig. 14B）和 PCK mRNA（Fig. 14D）作用无明显影响。

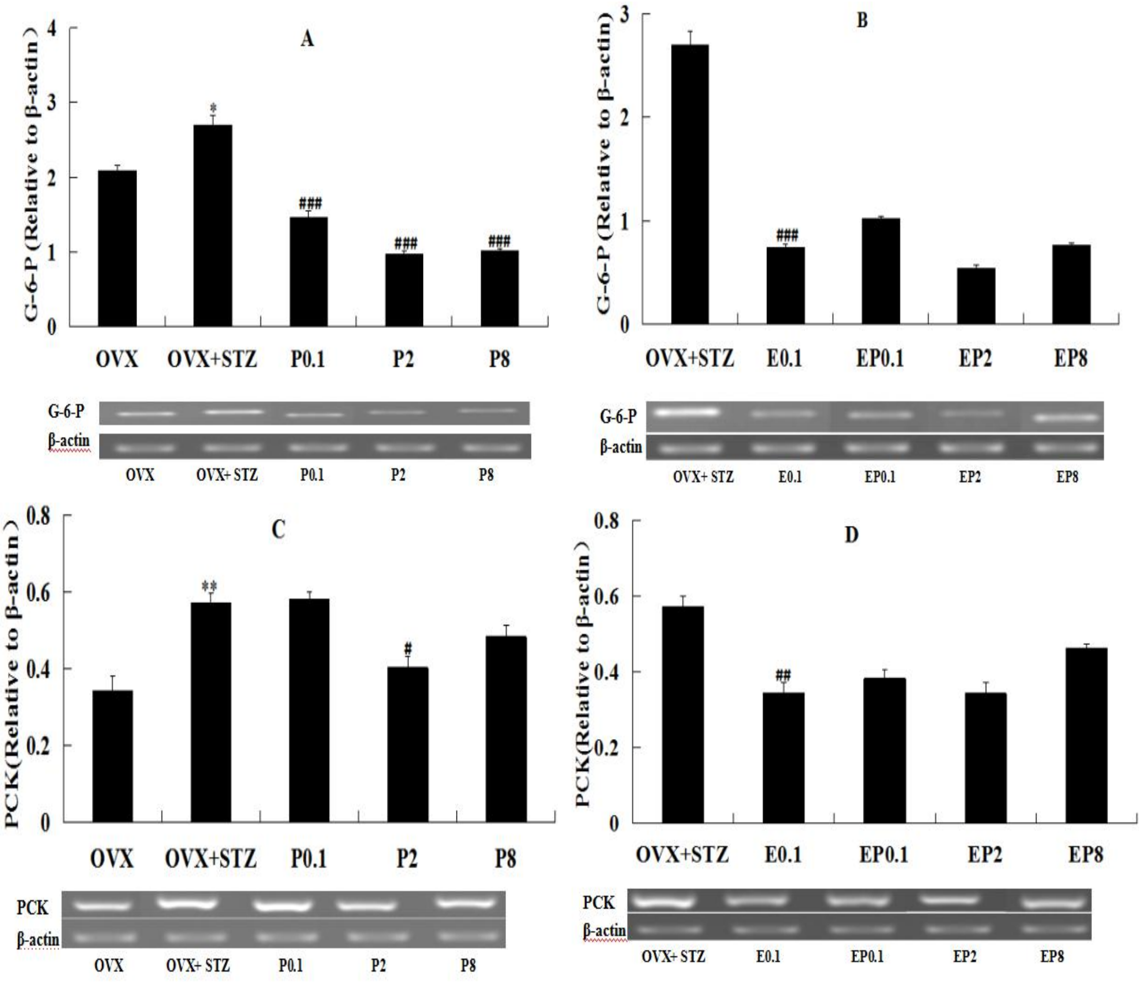

Fig. 14 The expressions of liver G-6-P and PCK mRNA

图 14 肝脏 G-6-P 和 PCK mRNA 的表达

The effects of progesterone and 17β-estradiol on liver G-6-P and PCK mRNA expressions were measured by RT- PCR in ovariectomized mice (OVX), in ovariectomized and streptozotocin-induced diabetes mice (OVX+STZ), in ovariectomized and streptozotocin-induced diabetes mice treated with 0.1mg/kg body weight progesterone (P0.1), 2mg/kg body weight progesterone (P2), 8mg/kg body weight progesterone (P8), 0.1mg/kg body weight 17β-estradiol (E0.1) or treated with the combination of 0.1mg/kg body weight 17β-estradiol and 0.1mg/kg body weight progesterone (EP0.1), 2mg/kg body weight progesterone (EP2) or 8mg/kg body weight progesterone (EP8). The results represent means±SD. (n=3). $^{*}P$<0.05, $^{**}P$<0.01 versus OVX; $^{\#}P$<0.05, $^{\#\#}P$<0.01, $^{\#\#\#}P$<0.001 versus OVX+STZ.

万方数据

第四章　讨　论

糖尿病（Diabetes mellitus，DM）是由遗传和环境等多种因素的共同作用引起，并且会导致多器官病变而严重危害患者健康的慢性疾病。1 型糖尿病患者的胰岛细胞因自身免疫性破坏，胰岛素绝对缺乏，血糖波动较大。流行病学调查显示[111]，约有 44％的绝经期女性存在胰岛素抵抗，绝经期女性与正常未绝经妇女相比，存在糖耐量受损，空腹血糖和胰岛素水平明显升高等现象，在对其进行雌激素替代治疗后，可增加其胰岛素分泌，降低血糖，改善糖耐量。已有动物实验证实[112]，雌性大鼠在卵巢切除后胰岛素敏感性、摄取葡萄糖和合成糖原的能力均降低，生理剂量的雌激素能够增强胰岛素的敏感性，而过高或过低剂量都会引起胰岛素抵抗。因此认为，雌激素可能在维持胰岛素正常敏感性方面起到一定的作用，但剂量效应关系和机制还不完全清楚。尽管较多的研究资料表明雌二醇（17β-estradiol，E）和黄体酮（Progesterone，P）可改善胰岛素抵抗，降低糖尿病的发病率，然而文献[113]报道妊娠期妇女体内相对较高水平的雌、孕激素可诱导机体产生胰岛素抵抗。可见雌激素和孕激素与糖尿病发生的关系与机体所处的生理状态及血中雌、孕激素水平有关。

4.1 小鼠 1 型糖尿病模型

1 型糖尿病是慢性疾病，其病因涉及遗传倾向以及促进疾病发展和发病的多种环境因素。1 型糖尿病的特征在于 T 细胞介导的β细胞自身免疫性破坏，胰岛素分泌不足[114]，临床治疗主要依赖外源性胰岛素，虽然 1 型糖尿病患者占糖尿病患者人数的比例不到 10%，但该病发病机制复杂且伴随多种并发症，严重影响患者的身体状况和生活质量。因此，对该病进行相关的研究对于预防和治疗此病具有重要意义。利用 STZ 造模是一种普遍使用的传统造模方法，STZ 能够选择性破坏胰岛β细胞，大剂量注射时可造成 1 型糖尿病模型[115,116]。本文通过腹腔注射 STZ，建立 1 型糖尿病模型，并根据 1 型和 2 型糖尿病的区别，通过胰岛素抵抗实验，来区别鉴定所建立糖尿病模型类型。结果表明，本文采用的建模方法可导致正常小鼠血糖升高，体重减轻，尿液明显增加，且与 OVX 正常组相比没有出现胰岛素抵抗，初步的实验结果符合 1 型糖尿病“三多一少的”基本表现，模型组血清 C 肽和胰岛素水平明显下降。说明该模型也符合 1 型糖尿病胰岛素分泌不足的本质特征。且本方法具有造模周期短，成模率高，可操作性强和重复性高，死亡率低（5.6%）等优点。

4.2 雌二醇对 STZ 所致小鼠 1 型糖尿病发生的延缓作用

雌激素通过雌激素受体（ER）可以保护胰腺β细胞并调节其功能，促进胰岛素分泌，提高胰岛素敏感性，改善肝脏、脂肪和骨骼肌等组织细胞的糖代谢功能，加强组

万方数据

织细胞对血液中葡萄糖的摄取和利用[117-118]。De Lauzon-Guillain[119]等人研究报道绝经后雌激素治疗能够降低女性糖尿病的发病率，本文通过腹腔注射 0.01、0.1 和 1mg/kg 体重的雌二醇，并检测血清中 17β-雌二醇和血糖含量，结果统计分析显示：低剂量（0.01mg/kg 体重）17β-雌二醇对糖尿病小鼠血糖没有明显的改善作用，0.1 和 1mg/kg 体重的 17β-雌二醇可明显降低糖尿病小鼠血糖，皮下注射 17β-雌二醇 0.1mg/kg 体重可以使小鼠血清雌二醇浓度达到生理浓度（186.53±66.11.3pg/mL）范围。

4.3 黄体酮对 STZ 所致小鼠 1 型糖尿病发生的延缓作用

黄体酮可能通过促进胰腺胰岛素的分泌，预防胰腺胰岛素合成受到损害而引起的 1 型糖尿病患者的血糖波动。有实验表明，对非孕妇给予黄体酮，能增加胰岛素分泌和糖原合成。孕妇易出现糖耐量异常或糖尿病已为临床所证实，且与孕妇体内雌激素、黄体酮、绒毛生长激素、肾上腺皮质激素抗胰岛素的分泌有关[119]。本文通过皮下注射 0.1、2 和 8mg/kg 体重黄体酮，同时检测其血清含量，结果发现可使血清含量达到生理范围内的黄体酮（黄体酮给药剂量 2mg/kg 体重时测得小鼠血清黄体酮含量为 1.77±1.3ng/mL，正常未切除卵巢小鼠血清黄体酮含量约为 4.3ng/mL，孕晚期小鼠血清黄体酮含量约为 20.08ng/mL）可以明显降低糖尿病小鼠的血糖。

4.4 17β-雌二醇和黄体酮联用对 STZ 所致小鼠 1 型糖尿病发生的延缓作用

雌激素和黄体酮低剂量联合治疗对胰岛素敏感性具有协同作用，其机制与促进 GLUT4 的易位，胰岛素与膜胰岛素受体（IR）的结合，与激活受体自磷酸化和受体酪氨酸激酶活性，胰岛素受体底物（IRS）的酪氨酸磷酸化有关，并能够改善胰岛素抵抗，降低糖尿病的发病率[120-123]，且联合时黄体酮可以降低雌激素治疗时女性患子宫内膜癌的风险[124-126]。本文将 0.1mg/kg 体重 17β-雌二醇分别与 0.1、2 和 8mg/kg 体重的黄体酮联合给药，结果显示低剂量和高剂量黄体酮与 17β-雌二醇联用时主要表现拮抗作用，而生理剂量的 17β-雌二醇与黄体酮联用可以协同保护胰岛β细胞，促进胰岛素分泌，改善尿糖现象，明显降低糖尿病小鼠血糖。

4.5 17β-雌二醇和黄体酮延缓糖尿病发生与胰岛β细胞的保护有关

17β-雌二醇能够保护胰岛β细胞，抑制其凋亡并促进胰岛素分泌，改善葡萄糖耐受和胰岛素抵抗[127]。Khoo[128]等人研究报道雌激素替代疗法可以控制高血糖并降低绝经后女性 2 型糖尿病的发病率。本文通过观察胰岛β细胞的 HE 染色，结果发现 17β-雌二醇和黄体酮能够保护腹腔注射 STZ 所引起的胰岛β细胞的损伤，促进其修复，增加胰岛β细胞的数量。同时，黄体酮、17β-雌二醇单独或二者联用可促进血清中 C 肽和胰岛素分泌，其中 2mg/kg 体重黄体酮，0.1mg/kg 体重 17β-雌二醇以及 0.1mg/kg

万方数据

体重 17β-雌二醇分别与 0.1 和 2mg/kg 体重黄体酮联用时促进 C 肽和胰岛素分泌的作用明显增强。通过胰岛素抵抗实验发现，糖尿病模型组小鼠与 OVX 对照组胰岛素抵抗和胰岛素受体表达无显著性差异。而糖耐量实验结果中，17β-雌二醇以及 17β-雌二醇和黄体酮联用可改善糖尿病小鼠糖耐量。因此，雌二醇和黄体酮通过保护胰岛β细胞，促进内源性胰岛素分泌，从而改善葡萄糖耐量，降低糖尿病小鼠血糖。

4.6 17β-雌二醇和黄体酮延缓糖尿病发生与促进糖转运有关

葡萄糖转运体 4（GLUT4）主要在骨骼肌（Skeletal muscle, SM）和白色脂肪组织(White adipose tissue, WAT)中表达，其表达、易位或对膜的锚定出现异常可导致胰岛素抵抗，扰乱葡萄糖稳态。当胰岛素与 SM 和 WAT 细胞膜上的受体结合时，可以启动磷酸化级联传导信号，使含有葡萄糖转运蛋白 GLUT4 的囊泡易位并锚定至细胞膜，从而将血液中的葡萄糖转运至细胞内供机体代谢利用。有研究发现骨骼肌 GLUT4 表达与雌激素受体 ERα和 ERβ相关，且两种雌激素受体对 GLUT4 调节作用相反。在 SM 中，ERα是 GLUT4 表达的正调控因子，而 ERβ具有抑制作用，雌激素的净效应取决于两种受体亚型之间的平衡[129]。

GLUT2 分布于肝细胞、胰岛细胞、肠上皮细胞基底外侧膜和肾小管上皮细胞。GLUT2 是一种低亲和力但具有很高转运能力的膜蛋白。在肝细胞中，GLUT2 具有双向转运葡萄糖的作用[130]，生理条件下，葡萄糖可经 GLUT2 转运进入细胞内，该蛋白与葡萄糖激酶共同形成葡萄糖感受器，GLUT2 与所处环境周围的葡萄糖浓度相适应，从而促进细胞膜两侧糖浓度的快速平衡。当血糖浓度发生变化时，GLUT2 能及时感应，从而对葡萄糖的转运也随之增加，在肝细胞和β细胞中 GLUT2 和高 km值的IV型葡萄糖激酶相配合，起葡萄糖感受器的作用。而 GLUT2 的转运速度往往高于葡萄糖激酶的反应速度，在肝细胞和β细胞中葡萄糖激酶是葡萄糖摄入的限制酶[131,132]。

本文中糖尿病小鼠模型组骨骼肌 GLUT4 mRNA 表达下调，肝脏 GLUT2 mRNA 表达上调，黄体酮、17β-雌二醇或二者联合均可促进骨骼肌 GLUT4 表达，下调肝脏 GLUT2 mRNA 表达。单独给药时，2mg/kg 体重黄体酮和 0.1mg/kg 体重 17β-雌二醇可显著上调骨骼肌 GLUT4 mRNA 的表达，下调肝脏 GLUT2 mRNA 表达，二者联合时 2mg/kg 体重黄体酮协同 17β-雌二醇促进 GLUT4 mRNA 的表达，而对 GLUT2 mRNA 表达表现出拮抗效果，但与 0.1mg/kg 体重 17β-雌二醇相比无显著性差异；0.1 和 8mg/kg 体重的黄体酮拮抗 17β-雌二醇对 GLUT4 mRNA 表达的上调作用。因此，17β-雌二醇、黄体酮及二者联合后降低血糖、延缓糖尿病发生的机制可能与其促进骨骼肌糖的利用和肝脏糖的储存有关。

万方数据

4.7 17β-雌二醇和黄体酮延缓糖尿病发生与肝脏糖代谢限速酶表达有关

葡萄糖激酶（Glucokinase , Gck）是肝细胞内主要的葡萄糖磷酸激酶，是一种 50kD 的单体代谢酶蛋白。Gck 主要在肝细胞和胰腺β细胞中表达，是糖酵解关键酶，催化葡萄糖磷酸化为葡萄糖-6-磷酸。参与糖酵解和糖原合成的第一步。在肝脏和胰腺β细胞中，Gck 是葡萄糖的传感器[126,128]。磷酸烯醇式丙酮酸羧激酶（Phosphoenolpyruvatecarboxykinase , PCK）是催化糖异生限速步骤，将草酰乙酸转化为磷酸烯醇式丙酮酸的限速酶，而糖异生关键酶葡萄糖-6-磷酸酶（Glucose-6-phosphatase, G-6-P）催化糖异生的最后一步，使葡萄糖 6 磷酸转化为葡萄糖。

本实验结果中模型组肝脏 PCK 和 G-6-P 比对照组表达明显升高，而两者的高表达可扰乱整个糖脂稳态，诱导葡萄糖失耐受、空腹高血糖和外周胰岛素抵抗。由于 STZ 建立的 1 型糖尿病可以直接或间接的影响肝脏糖异生关键酶 G-6-P 和 PCK 及糖酵解关键酶 Gck mRNA 的表达[130-133]。为进一步探讨黄体酮、17β-雌二醇及二者联合对糖尿病模型的影响，我们检测了肝脏 Gck、G-6-P 和 PCK mRNA 表达水平。本文中糖尿病模型组小鼠肝脏 Gck 表达显著下调，G-6-P 和 PCK 表达显著上调，部分给药组可上调 Gck 表达，下调 G-6-P 表达，其中 0.1 和 2mg/kg 体重黄体酮，0.1mg/kg 体重 17β-雌二醇显著上调 Gck，联合时 2mg/kg 体重黄体酮协同 17β-雌二醇上调 Gck 的表达，0.1 和 2mg/kg 体重黄体酮显著拮抗 17β-雌二醇对 Gck 表达的上调作用。0.1、2 和 8mg/kg 体重黄体酮及 0.1mg/kg 体重 17β-雌二醇均可下调 G-6-P 表达，联合时 2mg/kg 体重黄体酮协同 17β-雌二醇下调 G-6-P 的表达，0.1 和 8mg/kg 体重黄体酮拮抗 17β-雌二醇对 G-6-P 表达的下调作用。0.1mg/kg 体重 17β-雌二醇可显著下调 PCK 表达，而黄体酮单独给药或 17β-雌二醇与黄体酮联合时对 PCK 表达无显著性差异。因此，17β-雌二醇和黄体酮可能通过影响肝脏糖代谢酶 Gck、G-6-P、PCK 表达，促进肝糖原合成、抑制糖异生、影响肝脏糖代谢，降低血糖，延缓糖尿病的发生。

综上所述，17β-雌二醇和黄体酮单独给药均可延缓 STZ 所致小鼠 1 型糖尿病的发生，剂量不同作用表现不同，但不表现剂量效应关系。中剂量的 17β-雌二醇（0.1mg/kg 体重）和黄体酮（2mg/kg 体重）可使血药浓度达到生理剂量范围，其作用效果最佳；17β-雌二醇和黄体酮延缓糖尿病发生的机制是多方面的，可能与其保护胰岛细胞、促进胰岛素释放、改变骨骼肌葡萄糖转运及肝脏糖代谢有关；生理剂量的黄体酮与 17β-雌二醇联用对 17β-雌二醇延缓糖尿病的发生及影响葡萄糖转运蛋白和肝脏糖代谢限速酶的表达具有一定的协同效应，可加强 17β-雌二醇对糖尿病的预防作用。

万方数据

第六章 结 论

1. 17β-雌二醇和黄体酮单独给药均可延缓 STZ 所致小鼠 1 型糖尿病的发生，但不表现剂量效应关系；皮下注射中剂量的 17β-雌二醇（0.1mg/kg 体重）和黄体酮（2mg/kg 体重）可使血药浓度达到生理剂量范围，可明显抑制 STZ 所致小鼠血糖的升高；

2. 17β-雌二醇和黄体酮延缓糖尿病发生机制是多方面的，可能与其保护胰岛细胞、促进胰岛素释放、改变骨骼肌葡萄糖转运及肝脏糖代谢有关；

3. 生理剂量的黄体酮与 17β-雌二醇联用对 17β-雌二醇延缓糖尿病的发生及影响糖转运和糖代谢具有一定的协同效应，可加强 17β-雌二醇对糖尿病的预防作用。

万方数据

参考文献

1. Duarte-Guterman P, Navarro-Martín L, Trudeau V L. Mechanisms of crosstalk between endocrine systems: regulation of sex steroid hormone synthesis and action by thyroid hormones. Gen Comp Endocrinol. 2014; 203: 69.
2. Nouhjah S, Shahbazian H, Shahbazian N, Jahanshahi A, Jahanfar S, Cheraghian B. Incidence and Contributing Factors of Persistent Hyperglycemia at 6–12 Weeks Postpartum in Iranian Women with Gestational Diabetes: Results from LAGA Cohort Study. Diabetes Res. 2017; 9786436. doi:10. 1155/ 2017/ 9786436.

3 Nilsson S, Mäkelä S, Treuter E, Tujague M, Thomsen J, Andersson G. Mechanisms of estrogen action. Physiol Rev. 2001; 81: 1535–1565.

4. Safe S, Kim K. Non-classical genomic estrogen receptor (ER) /specificity protein and ER / activating protein-1 signaling pathways. Mol Endocrinol. 2008; 41: 263–275.
5. Kleinberger JW, and TI Pollin, Personalized medicine in diabetes mellitus: current opportunities and future prospects. Ann N Y Acad Sci. 2015; 1346(1): 45-56.
6. Paech K, Webb P, Kuiper GG, Nilsson S, Gustafsson J, Kushner PJ, Scanlan TS. Differential ligand activation of estrogen receptors ERα and ERβ at AP1 sites. Science. 1997; 277: 1508-1510.
7. Santen RJ, Allred DC, Ardoin SP, Archer DF, Boyd N, Braunstein GD, Burger HG, Colditz GA, Davis SR, Gambacciani M, Gower BA, Henderson VW, Jarjour WN, Karas RH, Kleerekoper M, Lobo RA, Manson JE, Marsden J, Martin KA, Martin L, Pinkerton JV, Rubinow DR, Teede H, Thiboutot DM, Utian WH; Endocrine Society. Postmenopausal hormone therapy: an Endocrine Society scientific statement. Clin Endocrinol Metab. 2010; 95(7 suppl 1): s1–s66.
8. Powell E, Xu W. Intermolecular interactions identify ligand-selective activity of estrogen receptor α/β dimers. Proc Natl Acad Sci USA. 2008; 105: 19012–19017.
9. Charn TH, Liu ET, Chang EC, Lee YK, Katzenellenbogen JA, Katzenellenbogen BS. Genome-wide dynamics of chromatin binding of estrogen receptors α and β: mutual restriction and competitive site selection. Mol Endocrinol. 2010; 24: 47–59.
10. Shang Y, Hu X, DiRenzo J, Lazar MA, Brown M. Cofactor dynamics and sufficiency in estrogen receptor-regulated transcription. Cell. 2000; 103: 843–852.
11. Carr MC. The emergence of the metabolic syndrome with menopause. Clin Endocrinol Metab. 2003; 88: 2404–2411.
12. Blaustein JD, Wade GN. Ovarian influences on the meal patterns of female rats. Physiol Behav. 1976; 17: 201–208.
13. Drewett RF. Sexual behaviour and sexual motivation in the female rat. Nature. 1973; 242: 476–477.
14. Wallen WJ, Belanger MP, Wittnich C. Sex hormones and the selective estrogen receptor modulator tamoxifen modulate weekly body weights and food intakes in adolescent and adult rats. Nutr. 2001; 131: 2351–2357.
15. Geary N, Asarian L, Korach KS, Pfaff DW, Ogawa S. Deficits in E2-dependent control of feeding, weight gain, and cholecystokinin satiation in ER-α null mice. Endocrinology. 2001; 142: 4751–4757.

万方数据

16. Gao Q, Mezei G, Nie Y, Rao Y, Choi CS, Bechmann I, Leranth C, Toran-Allerand D, Priest CA, Roberts JL, Gao XB, Mobbs C, Shulman GI, Diano S, Horvath TL. Anorectic estrogen mimics leptin's effect on the rewiring of melanocortin cells and Stat3 signaling in obese animals. Nat Med. 2007; 13: 89–94.

17. Clegg DJ, Riedy CA, Smith KA, Benoit SC, Woods SC. Differential sensitivity to central leptin and insulin in male and female rats. Diabetes. 2003; 52: 682–687.

18. Jones ME, Thorburn AW, Britt KL, Hewitt KN, Wreford NG, Proietto J, Oz OK, Leury BJ, Robertson KM, Yao S, Simpson ER. Aromatase-deficient (ArKO) mice have a phenotype of increased adiposity. Proc Natl Acad Sci USA. 2000; 97: 12735–12740.

19. Heine PA, Taylor JA, Iwamoto GA, Lubahn DB, Cooke PS. Increased adipose tissue in male and female estrogen receptor-α knockout mice. Proc Natl Acad Sci USA. 2000; 97: 12729–12734.

20. Ribas V, Nguyen MT, Henstridge DC, Nguyen AK, Beaven SW, Watt MJ, Hevener AL. Impaired oxidative metabolism and inflammation are associated with insulin resistance in ERα deficient mice. Am J Physiol Endocrinol Metab. 2010; 298: E304–E319.

21. Rogers NH, Perfield JW, II, Strissel KJ, Obin MS, Greenberg AS. Reduced energy expenditure and increased inflammation are early events in the development of ovariectomy-induced obesity. Endocrinology. 2009; 150: 2161–2168.

22. Smith GP. The controls of eating: a shift from nutritional homeostasis to behavioral neuroscience. Nutrition. 2000; 16: 814–820.

23. Lisa M Davis, Michael Michaelides, Lawrence J Cheskin, Timothy H Moran, Susan Aja, Paul A. Watkins, Zhengtong Pei, Carlo Contoreggi, Karen McCullough, Bruce Hope, Gene Jack Wang, Nora D Volkow, Panayotis K Thanos. Bromocriptine administration reduces hyperphagia and adiposity and differentially affects dopamine D2 receptor and transporter binding in leptin-receptor-deficient Zucker rats and rats with diet-induced obesity. Neuroendocrinology, 2009, 89(2): 152-162.

24. Ritchie E Brown, Radhika Basheer, James T McKenna, Robert E Strecker, Robert W McCarley. Control of sleep and wakefulness. Physiol Rev. 2012; 92(3): 1087.

25. Kim Y J, Bi S. Knockdown of neuropeptide Y in the dorsomedial hypothalamus reverses high fat diet-induced obesity and impaired glucose tolerance in rats. Am J Physiol Regul Integr Comp Physiol. 2015; 310(2): ajpregu. 00174.

26. Frank A, Brown L M, Clegg D J. The Role of Hypothalamic Estrogen Receptors in Metabolic Regulation. Front Neuroendocrinol. 2014; 35(4): 550-557.

27. Merchenthaler I, Lane MV, Numan S, Dellovade TL. Distribution of estrogen receptor α and β in the mouse central nervous system: in vivo autoradiographic and immunocytochemical analyses. J Comp Neurol. 2004; 473: 270–291.

28. Lin Zhu, Melissa N. Martinez, Christopher H. Emfinger, Brian T. Palmisano, John M. Stafford. Estrogen signaling prevents diet-induced hepatic insulin resistance in male mice with obesity. Am J Physiol Endocrinol Metab. 2014, 306(10): E1188.

29. Roesch DM. Effects of selective estrogen receptor agonists on food intake and body weight gain in rats. Physiol Behav. 2006; 87: 39–44.

30. Santollo J, Wiley MD, Eckel LA. Acute activation of ER α decreases food intake, meal size, and body weight in ovariectomized rats. Am J Physiol Regul Integr Comp Physiol. 2007; 293:

万方数据

R2194–R2201.

31. Liang YQ, Akishita M, Kim S, Ako J, Hashimoto M, Iijima K, Ohike Y, Watanabe T, Sudoh N, Toba K, Yoshizumi M, Ouchi Y. Estrogen receptor β is involved in the anorectic action of estrogen. Int J Obes Relat Metab Disord. 2002; 26: 1103–1109.
32. Yepuru M, Eswaraka J, Kearbey JD, Barrett CM, Raghow S, Veverka KA, Miller DD, Dalton JT, Narayanan R. Estrogen receptor-β selective ligands alleviate high-fat-diet and ovariectomy-induced obesity in mice. J Biol Chem. 2010; 285: 31292–31303.
33. Langer G, Bader B, Meoli L, Isensee J, Delbeck M, Noppinger PR, Otto C. A critical review of fundamental controversies in the field of GPR30 research. Steroids. 2010; 75: 603–610.
34. Elmquist JK, Elias CF, Saper CB. From lesions to leptin: hypothalamic control of food intake and body weight. Neuron. 1999; 22: 221–232.
35. Schwartz MW, Woods SC, Porte DJ, Seeley RJ, Baskin DG. Central nervous system control of food intake. Nature. 2000; 404: 661–671.
36. Myers MG, Jr, Leibel RL, Seeley RJ, Schwartz MW. Obesity and leptin resistance: distinguishing cause from effect. Trends Endocrinol Metab. 2010; 21: 643–651.
37. Van Dijk G, Thiele TE, Donahey JC, Campfield LA, Smith FJ, Burn P, Bernstein IL, Woods SC, Seeley RJ. Central infusion of leptin and GLP-1 (7–36) amide differentially stimulate c-Fos-like immunoreactivity in the rat brain. Am J Physiol. 1996; 271: R1096–R1100.
38. Desai B N, Harris R B. Integrated Effects of Leptin in the Forebrain and Hindbrain of Male Rats. Endocrinology. 2013; 154(8): 2663.
39. Haifei Shi, Joyce E. Sorrell, Deborah J. Clegg, Stephen C. Woods, Randy J. Seeley. The roles of leptin receptors on POMC neurons in the regulation of sex-specific energy homeostasis. Physiol Behav. 2010, 100(2): 165.
40. Bennett PA, Lindell K, Wilson C, Carlsson LM, Carlsson B, Robinson IC. Cyclical variations in the abundance of leptin receptors, but not in circulating leptin, correlate with NPY expression during the oestrous cycle. Neuroendocrinology. 1999; 69: 417–423.
41. Lindell K, Bennett PA, Itoh Y, Robinson IC, Carlsson LM, Carlsson B. Leptin receptor 5′untranslated regions in the rat: relative abundance, genomic organization and relation to putative response elements. Mol Cell Endocrinol. 2001; 172: 37–45.
42. Ainslie DA, Morris MJ, Wittert G, Turnbull H, Proietto J, Thorburn AW. Estrogen deficiency causes central leptin insensitivity and increased hypothalamic neuropeptide Y. Int J Obes Relat Metab Disord. 2001; 25: 1680–1688.
43. Clegg DJ, Brown LM, Woods SC, Benoit SC. Gonadal hormones determine sensitivity to central leptin and insulin. Diabetes. 2006; 55: 978–987.
44. Chavez M, van Dijk G, Arkies BJ, Woods SC. 1995 ventricular insulin infusion attenuates NPY-induced feeding at the level of the paraventricular nucleus. Obes Res. 1995; 3: 335.
45. Shin J Lee, Saurabh Verma, Stephanie E Simonds, Melissa A Kirigiti, Paul Kievit, Sarah R Lindsley, Alberto Loche, M Susan Smith, Michael A Cowley, Kevin L Grove. Leptin Stimulates Neuropeptide Y and Cocaine Amphetamine-Regulated Transcript Coexpressing Neuronal Activity in the Dorsomedial Hypothalamus in Diet-Induced Obese Mice. J Neurosci. 2013; 33(38): 15306-17.
46. Cone RD, Cowley MA, Butler AA, Fan W, Marks DL, Low MJ. The arcuate nucleus as a conduit

万方数据

for diverse signals relevant to energy homeostasis. Int J Obes Relat Metab Disord. 2001; 25(suppl 5): S63–S67.

47. Herzog H. Neuropeptide Y and energy homeostasis: insights from Y receptor knockout models. Eur J Pharmacol. 2003; 480: 21–29.

48. Nadal A, Alonso-Magdalena P, Soriano S, Quesada I, Ropero AB. The pancreatic β-cell as a target of estrogens and xenoestrogens: implications for blood glucose homeostasis and diabetes. Mol Cell Endocrinol. 2009; 304: 63–68.

49. Barros RP, Morani A, Moriscot A, Machado UF. Insulin resistance of pregnancy involves estrogen-induced repression of muscle GLUT4. Mol Cell Endocrinol. 2008; 295: 24–31.

50. Wild SH, Roglic G, Green A, Sicree R, King H. Global prevalence of diabetes: estimates for the year 2000 and projections for 2030. Diabetes Care. 2004; 27(5): 1047–1053.

51. Health Quality Ontario. Pancreas Islet Transplantation for Patients With Type 1 Diabetes Mellitus: A Clinical Evidence Review. Ontario Health Technology Assessment Series. 2015; 15(16): 1-84.

52. International Diabetes Federation. IDF Diabetes Atlas: Global estimates of diabetes prevalence for 2017 and projections for 2045.

53. Neth BJ, Craft S. Insulin Resistance and Alzheimer’s Disease: Bioenergetic Linkages. Frontiers in Aging Neuroscience. 2017; 9: 345.

54. Naylor RN, Greeley SAW, Bell GI, Genetics and pathophysiology of neonatal diabetes mellitus.Philipson LH. Journal of Diabetes Investigation. 2011; 2(3): 158-169. doi:10.1111/j. 2040-1124. 2011. 00106. x.

55. Greeley SAW, Naylor RN, Philipson LH, Bell GI. Neonatal Diabetes: An Expanding List of Genes Allows for Improved Diagnosis and Treatment. Current diabetes reports. 2011; 11(6): 519-532. doi: 10. 1007/s11892-011-0234-7.

56. Aune D, Sen A, Henriksen T, Saugstad OD, Tonstad S. Physical activity and the risk of gestational diabetes mellitus: a systematic review and dose–response meta-analysis of epidemiological studies. European Journal of Epidemiology. 2016; 31(10): 967-997. doi: 10. 1007/s10654-016-0176-0.

57. Abell SK, De Courten B, Boyle JA, Teede HJ. Inflammatory and Other Biomarkers: Role in Pathophysiology and Prediction of Gestational Diabetes Mellitus. Baker PN, ed. International Journal of Molecular Sciences. 2015; 16(6): 13442-13473.

58. Nouhjah S, Shahbazian H, Shahbazian N, Jahanshahi A, Jahanfar S, Cheraghian B. Incidence and Contributing Factors of Persistent Hyperglycemia at 6–12 Weeks Postpartum in Iranian Women with Gestational Diabetes: Results from LAGA Cohort Study. Diabetes Research. 2017; 2017: 9786436. doi: 10.1155/2017/9786436.

59. Sun J, Zhao M, Jia P, Wang L, Wu Y, Iverson C, Zhou Y, Bowton E, Roden DM, Denny JC, Aldrich MC, Xu H, Zhao Z. Deciphering Signaling Pathway Networks to Understand the Molecular Mechanisms of Metformin Action. Zhou XJ, ed. PLoS Computational Biology. 2015; 11(6): e1004202.

60. Nakamura K, Oe H, Kihara H, Shimada K, Fukuda S, Watanabe K, Takagi T, Yunoki K, Miyoshi T, Hirata K, Yoshikawa J, Ito H. DPP-4 inhibitor and alpha-glucosidase inhibitor equally improve endothelial function in patients with type 2 diabetes: EDGE study. Cardiovascular Diabetology. 2014; 13: 110.

万方数据

61. Bhargava - Shah A, Foygel K, Devulapally R, Paulmurugan R. Orlistat and antisense - miRNA-loaded PLGA-PEG nanoparticles for enhanced triple negative breast cancer therapy. Nanomedicine. 2016; 11: 235-247.
62. Park YW, Zhu S, Palaniappan L, Heshka S, Carnethon MR, Heymsfield SB. The metabolic syndrome: prevalence and associated risk factor findings in the US population from the Third National Health and Nutrition Examination Survey, 1988–1994. Arch Intern Med. 2003; 163: 427–436.
63. Yki-Jarvinen H. Sex and insulin sensitivity. Metabolism. 1984; 33: 1011–1015.
64. Hevener AL, Olefsky JM, Reichart D, Nguyen MT, Bandyopadyhay G, Leung HY, Watt MJ, Benner C, Febbraio MA, Nguyen AK, Folian B, Subramaniam S, Gonzalez FJ, Glass CK, Ricote M. Macrophage PPAR γ is required for normal skeletal muscle and hepatic insulin sensitivity and full antidiabetic effects of thiazolidinediones. J Clin Invest. 2007; 117: 1658–1669.
65. Choi CS, Fillmore JJ, Kim JK, Liu ZX, Kim S, Collier EF, Kulkarni A, Distefano A, Hwang YJ, Kahn M, Chen Y, Yu C, Moore IK, Reznick RM, Higashimori T, Shulman GI. Overexpression of uncoupling protein 3 in skeletal muscle protects against fat-induced insulin resistance. J Clin Invest. 2007; 117: 1995–2003.
66. Hong J, Stubbins RE, Smith RR, Harvey AE, Nunez NP. Differential susceptibility to obesity between male, female and ovariectomized female mice. Nutr J. 2009; 8: 11.
67. Frias JP, Macaraeg GB, Ofrecio J, Yu JG, Olefsky JM, Kruszynska YT. Decreased susceptibility to fatty acid-induced peripheral tissue insulin resistance in women. Diabetes. 2001; 50: 1344–1350.
68. Andrei Mihai Malutan, Mihu Dan, Costin Nicolae, Mihu Carmen. Proinflammatory and anti-inflammatory cytokine changes related to menopause. Prz Menopauzalny. 2014; 13(3): 162-168.
69. Sites CK, Toth MJ, Cushman M, L'Hommedieu GD, Tchernof A, Tracy RP, Poehlman ET. Menopause-related differences in inflammation markers and their relationship to body fat distribution and insulin-stimulated glucose disposal. Fertil Steril. 2002; 77: 128–135.
70. Campbell SE, Febbraio MA. Effect of the ovarian hormones on GLUT4 expression and contraction-stimulated glucose uptake. Am J Physiol Endocrinol Metab. 2002; 282: E1139–E1146.
71. Stubbins RE, Holcomb VB, Hong J, Nunez NP. Estrogen modulates abdominal adiposity and protects female mice from obesity and impaired glucose tolerance. Eur J Nutr. 2012; 51: 861–870.
72. Ding EL, Song Y, Manson JE, Rifai N, Buring JE, Liu S. Plasma sex steroid hormones and risk of developing type 2 diabetes in women: a prospective study. Diabetologia. 2007; 50: 2076–2084.
73. Kalyani RR, Franco M, Dobs AS, Ouyang P, Vaidya D, Bertoni A, Gapstur SM, Golden SH. The association of endogenous sex hormones, adiposity, and insulin resistance with incident diabetes in postmenopausal women. J Clin Endocrinol Metab. 2009; 94: 4127–4135.
74. Bryzgalova G, Gao H, Ahren B, Zierath JR, Galuska D, Steiler TL, Dahlman-Wright K, Nilsson S, Gustafsson JA, Efendic S, Khan A. Evidence that oestrogen receptor-α plays an important role in the regulation of glucose homeostasis in mice: insulin sensitivity in the liver. Diabetologia. 2006; 49: 588–597.
75. Brown R J, Cochran E, Gorden P. Metreleptin improves blood glucose in patients with insulin

万方数据

receptor mutations. J Clin Endocrinol Metab. 2013; 98(11): 1749-56.

76. Hong J, Stubbins RE, Smith RR, Harvey AE, Nunez NP. Differential susceptibility to obesity between male, female and ovariectomized female mice. Nutr J. 2009; 8: 11.

77. Bryzgalova G, Lundholm L, Portwood N, Gustafsson JA, Khan A, Efendic S, Dahlman-Wright K. Mechanisms of antidiabetogenic and body weight-lowering effects of estrogen in high-fat diet-fed mice. Am J Physiol Endocrinol Metab. 2008; 295: E904–E912.

78. Gao H, Bryzgalova G, Hedman E, Khan A, Efendic S, Gustafsson JA, Dahlman-Wright K. Long-term administration of estradiol decreases expression of hepatic lipogenic genes and improves insulin sensitivity in ob/ob mice: a possible mechanism is through direct regulation of signal transducer and activator of transcription 3. Mol Endocrinol. 2006; 20: 1287–1299.

79. Lundholm L, Bryzgalova G, Gao H, Portwood N, Fält S, Berndt KD, Dicker A, Galuska D, Zierath JR, Gustafsson JA, Efendic S, Dahlman-Wright K, Khan A. The estrogen receptor α-selective agonist propyl pyrazole triol improves glucose tolerance in ob/ob mice; potential molecular mechanisms. J Endocrinol. 2008; 199: 275–286.

80. Riant E, Waget A, Cogo H, Arnal JF, Burcelin R, Gourdy P. Estrogens protect against high-fat diet-induced insulin resistance and glucose intolerance in mice. Endocrinology. 2009; 150: 2109–2117.

81. Tiano JP, Mauvais-Jarvis F. Importance of oestrogen receptors to preserve functional β-cell mass in diabetes. Nat Rev Endocrinol. 2012; 8: 342–351.

82. Wong WP, Tiano JP, Liu S, Hewitt SC, Le May C, Dalle S, Katzenellenbogen JA, Katzenellenbogen BS, Korach KS, Mauvais-Jarvis F. Extranuclear estrogen receptor-α stimulates NeuroD binding to the insulin promoter and favors insulin synthesis. Proc Natl Acad Sci USA. 2010; 107: 13057–13062.

83. Tiano JP, Mauvais-Jarvis F. Molecular mechanisms of estrogen receptors' suppression of lipogenesis in pancreatic β-cells. Endocrinology. 2012; 153: 2997–3005.

84. Alonso-Magdalena P, Ropero AB, Carrera MP, Cederroth CR, Baquié M, Gauthier BR, Nef S, Stefani E, Nadal A. Pancreatic insulin content regulation by the estrogen receptor ER α. Plos One. 2008; 3: e2069.

85. Tiano JP, Delghingaro-Augusto V, Le May C, Liu S, Kaw MK, Khuder SS, Latour MG, Bhatt SA, Korach KS, Najjar SM, Prentki M, Mauvais-Jarvis F. Estrogen receptor activation reduces lipid synthesis in pancreatic islets and prevents β cell failure in rodent models of type 2 diabetes. J Clin Invest. 2011; 121: 3331–3342.

86. Choe SS, Choi AH, Lee JW, Kim KH, Chung JJ, Park J, Lee KM, Park KG, Lee IK, Kim JB. Chronic activation of liver X receptor induces β-cell apoptosis through hyperactivation of lipogenesis: liver X receptor-mediated lipotoxicity in pancreatic β-cells. Diabetes. 2007; 56: 1534–1543.

87. Soriano S, Ropero AB, Alonso-Magdalena P, Ripoll C, Quesada I, Gassner B, Kuhn M, Gustafsson JA, Nadal A. Rapid regulation of K(ATP) channel activity by 17β-estradiol in pancreatic β-cells involves the estrogen receptor β and the atrial natriuretic peptide receptor. Mol Endocrinol. 2009; 23: 1973–1982.

88. Soriano S, Alonso-Magdalena P, García-Arévalo M, Novials A, Muhammed SJ, Salehi A, Gustafsson JA, Quesada I, Nadal A. Rapid insulinotropic action of low doses of bisphenol-A on

万方数据

mouse and human islets of Langerhans: role of estrogen receptor β. Plos One. 2012; 7: e31109.

89. Tiano J, Mauvais-Jarvis F. Selective estrogen receptor modulation in pancreatic β-cells and the prevention of type 2 diabetes. Islets. 2012; 4: 173–176.

90. Balhuizen A, Kumar R, Amisten S, Lundquist I, Salehi A. Activation of G protein-coupled receptor 30 modulates hormone secretion and counteracts cytokine-induced apoptosis in pancreatic islets of female mice. Mol Cell Endocrinol. 2010; 320: 16–24.

91. Kumar R, Balhuizen A, Amisten S, Lundquist I, Salehi A. Insulinotropic and antidiabetic effects of 17β-estradiol and the GPR30 agonist G-1 on human pancreatic islets. Endocrinology. 2011; 152: 2568–2579.

92. Sharma G, Prossnitz ER. Mechanisms of estradiol-induced insulin secretion by the G protein-coupled estrogen receptor GPR30/GPER in pancreatic β-cells. Endocrinology. 2011; 152: 3030–3039.

93. Kang L, Zhang X, Xie Y, Tu Y, Wang D, Liu Z, Wang ZY. Involvement of estrogen receptor variant ER-α36, not GPR30, in nongenomic estrogen signaling. Mol Endocrinol. 2010; 24: 709–721.

94. Contreras JL, Smyth CA, Bilbao G, Young CJ, Thompson JA, Eckhoff DE. 17β-Estradiol protects isolated human pancreatic islets against proinflammatory cytokine-induced cell death: molecular mechanisms and islet functionality. Transplantation. 2002; 74: 1252–1259.

95. Salonia A, Lanzi R, Scavini M, Pontillo M, Gatti E, Petrella G, Licata G, Nappi RE, Bosi E, Briganti A, Rigatti P, Montorsi F. Sexual function and endocrine profile in fertile women with type 1 diabetes. Diabetes Care. 2006; 29: 312–316.

96. DiMarchi RD, Finan B, Yang B, Ottaway N, Stemmer K, Müller TD, Yi CX, Habegger K, Schriever SC, García-Cáceres C, Kabra DG, Hembree J, Holland J, Raver C, Seeley RJ, Hans W, Irmler M, Beckers J, de Angelis MH, Tiano JP, Mauvais-Jarvis F, Perez-Tilve D, Pfluger P, Zhang L, Gelfanov V, Tschöp MH. Targeted estrogen delivery reverses the metabolic syndrome. Nat Med. 2012; 18: 1847–1856.

97. Tiano J, Finan B, DiMarchi R, Mauvais-Jarvis F. A glucagon-like peptide-1-estrogen fusion peptide shows enhanced efficacy in preventing insulin-deficient diabetes in mice. Endocr Rev. 2012; 33: OR21–OR26.

98. Yuefeng Rao, Feiyue Zheng, Xingguo Zhang, Jianqing Gao, Wenquan Liang. In vitro percutaneous permeation and skin accumulation of finasteride using vesicular ethosomal carriers. AAPS PharmSciTech. 2008; 9(3): 860-865.

99. Good WR, John VA, Ramirez M, Higgins JE. Comparison of Alora estradiol matrix transdermal delivery system with oral conjugated equine estrogen therapy in relieving menopausal symptoms. Alora Study Group. Climacteric. 1999; 2: 29–36.

100. James H. Pickar, Charles Bon, Julia M. Amadio, Sebastian Mirkin. Pharmacokinetics of the first combination 17β-estradiol/progesterone capsule in clinical development for menopausal hormone therapy. Brian Bernick Menopause. 2015; 22(12): 1308-1316.

101. Casanova G, Spritzer P M. Effects of micronized progesterone added to non-oral estradiol on lipids and cardiovascular risk factors in early postmenopause: a clinical trial. Lipids Health Dis. 2012; 11(1):133.

102. Chu MC, Cosper P, Nakhuda GS, Lobo RA. A comparison of oral and transdermal short-term

万方数据

estrogen therapy in postmenopausal women with metabolic syndrome. Fertil Steril. 2006; 86: 1669–1675.

103. Chu MC, Cushman M, Solomon R, Lobo RA. Metabolic syndrome in postmenopausal women: the influence of oral or transdermal estradiol on inflammation and coagulation markers. Am J Obstet Gynecol. 2008; 199: 526. e1–526. e7.

104. Salpeter SR, Walsh JM, Ormiston TM, Greyber E, Buckley NS, Salpeter EE. Meta-analysis: effect of hormone-replacement therapy on components of the metabolic syndrome in postmenopausal women. Diabetes Obes Metab. 2006; 8: 538–554.

105. Salpeter SR, Cheng J, Thabane L, Buckley NS, Salpeter EE. Bayesian meta-analysis of hormone therapy and mortality in younger postmenopausal women. Am J Med. 2009; 122: 1016–1022.

106. Rossouw JE, Anderson GL, Prentice RL, LaCroix AZ, Kooperberg C, Stefanick ML, Jackson RD, Beresford SA, Howard BV, Johnson KC, Kotchen JM, Ockene J, Writing Group for the Women's Health Initiative Investigators. Risks and benefits of estrogen plus progestin in healthy postmenopausal women: principal results from the Women's Health Initiative randomized controlled trial. JAMA. 2002; 288: 321–333.

107. Khalil R A. Estrogen, vascular estrogen receptor and hormone therapy in postmenopausal vascular disease[J]. Biochem Pharmacol. 2013; 86(12): 1627-42.

108. Herrington DM, Vittinghoff E, Lin F, Fong J, Harris F, Hunninghake D, Bittner V, Schrott HG, Blumenthal RS, Levy R; HERS Study Group. Statin Therapy, Cardiovascular Events, and Total Mortality in the Heart and Estrogen/Progestin Replacement Study (HERS). Circulation. 2002; 105(25): 2962-2967.

109. Andrea Z LaCroix, Rowan T Chlebowski, JoAnn E Manson, Aaron K Aragaki, Karen C Johnson, Lisa Martin, Karen L Margolis, Marcia L Stefanick, Robert Brzyski, J David Curb, Barbara V Howard, Cora E Lewis, Jean Wactawski-Wende. Health Risks and Benefits after Stopping the Women's Health Initiative Trial of Conjugated Equine Estrogens in Postmenopausal Women with Prior Hysterectomy. JAMA. 2011; 305: 1305–1314.

110. Michael Y L, Gold R, Manson J A E. Hormone therapy and physical function change among older women in the Women's Health Initiative: a randomized controlled trial. Menopause. 2010; 17(2): 295.

111. Susan B Racette, Ellen M Evans, Edward P Weiss, James M Hagberg, John O Holloszy. Abdominal Adiposity Is a Stronger Predictor of Insulin Resistance Than Fitness Among 50–95 Year Old. Diabetes Care. 2006; 29(3): 673-678.

112. Barros R P, Gabbi C, Morani A, et al. Participation of ERalpha and ERbeta in glucose homeostasis in skeletal muscle and white adipose tissue[J]. American Journal of Physiology Endocrinology & Metabolism, 2009, 297(1):E124.

113. Bone HG, Lindsay R, McClung MR, Perez AT, Raanan MG, Spanheimer RG. Effects of pioglitazone on bone in postmenopausal women with impaired fasting glucose or impaired glucose tolerance: a randomized, double-blind, placebo-controlled study. J Clin Endocrinol Metab. 2013; 98(12): 4691-701.

114. Archer J, S Coffler M, Yu J, Wachs D, Yoo R & Chang R. Effect of estrogen and progesterone on free fatty acid-induced insulin resistance in postmenopausal women. Fertility & Sterility, 2005; 84(141), S117-S118.

万方数据

115. Bo Yi, Gan Huang, and Zhiguang Zhou. Different Role of Zinc Transporter 8 between Type 1 Diabetes Mellitus and Type 2 Diabetes Mellitus. J Diabetes Investigation. 2016; 7(4): 459–465.

116. Jiayin Zheng, Yue Wang, Song Han,Yanlin Luo, Xiuli Sun, Ning Zhu,Li Zhao, and Junfa Li. Identification of Protein Kinase C Isoforms Involved in Type 1 Diabetic Encephalopathy in Mice. J Diabetes Res. doi: 10.1155/2018/8431249.

117. Labazi H, Teng B, Mustafa S J. Functional changes in vascular reactivity to adenosine receptor activation in type I diabetic mice. European Journal of Pharmacology. 2018; 820:191-197.

118. Faulds MH, Zhao C, Dahlman-Wright K, Gustafsson JÅ. The diversity of sex steroid action: regulation of metabolism by estrogen signaling. Endocrinol. 2012; 212: 3–12.

119. De Lauzon-Guillain B, Fournier A, Fabre A, Simon N, Mesrine S, Boutron-Ruault MC. Menopausal hormone therapy and new-onset diabetes in the French Etude Epidemiologique de Femmes de la Mutuelle Générale de l’Education Nationale (E3N) cohort. J Diabetologia. 2009; 52(10): 2092–2100.

120. Pang Y, Thomas P. Additive effects of low concentrations of estradiol-17β and progesterone on nitric oxide production by human vascular endothelial cells through shared signaling pathways. J Steroid Biochemistry & Molecular Biology. 2016; 165(Pt B).

121. J de Castro, J Sevillano, J Marciniak, R Rodriguez, C González-Martín, M Viana, O H Eun-suk, S Hauguel de Mouzon, E Herrera, M P Ramos. Implication of low level inflammation in the insulin resistance of adipose tissue at late pregnancy. Endocrinology. 2011; 152(11): 4094-4105.

122. S Wild, G Roglic, A Green, R Sicree, H King, Global prevalence of diabetes: estimates for the year 2000 and projections for 2030. Diabetes Care. 2004; 27 (5): 1047–1053.

123. Alexandra Kautzky-Willer, Jürgen Harreiter, Giovanni Pacini. Sex and Gender Differences in Risk, Pathophysiology and Complications of Type 2 Diabetes Mellitus. Endocr Rev. 2016; 37(3): 278-316.

124. K L Margolis, D E Bonds, R J Rodabough, L Tinker, L S Phillips, C Allen, T Bassford, G Burke, J Torrens, B V Howard, Women’s Health Initiative Investigators. Effect of oestrogen plus progestin on the incidence of diabetes in postmenopausal women: results from the Women’s Health Initiative Hormone Trial. Diabetologia. 2004; 47 (7) : 1175–1187.

125. D C Felmeden, G Y Lip. Hormone replacement therapy and hypertension. Blood Press. 2009; 246–249.

126. B Ettinger, Rationale for use of lower estrogen doses for postmenopausal hormone therapy. Maturitas. 2007; 57: 81–84.

127. Jazbutyte, V Arias-Loza, P A, Hu K, Widder J Govindaraj V, & Von, P C. Ligand-dependent activation of ERβ lowers blood pressure and attenuates cardiac hypertrophy in ovariectomized spontaneously hypertensive rats. Cardiovascular Research. 2008; 77(4): 774-81.

128. Khoi Chu, Cedric Le May,Min Hu, Christina S Ortega, Evan R. Simpson, Kenneth S Korach, Ming-Jer Tsai, Franck Mauvais-Jarvis. Estrogens protect pancreatic β-cells from apoptosis and prevent insulin-deficient diabetes mellitus in mice. Proc Natl Acad Sci U S A. 2006; 103(24): 9232–9237.

129. Catherine Kim, Sharon L. Edelstein, Jill P Crandall, Dana Dabelea, Abbas E Kitabchi, Richard FHamman, Maria G Montez, Leigh Perreault, Mary A. Foulkes, Elizabeth Barrett-Connor. Menopause and risk of diabetes in the Diabetes Prevention Program. Menopause. 2011; 18(8):

万方数据

857-68.

130. Sung T C, Huang J W, Guo H R. Association between Arsenic Exposure and Diabetes: A Meta-Analysis. Biomed Res Int. 2015; 2015(17): 368087.

131. Thorens B. GLUT2, glucose sensing and glucose homeostasis. Diabetologia. 2015; 58: 221–32.

132. Christopher P. Corpe, Peter Eck, Jin Wang, Hadi Al-Hasani, and Mark Levine. Intestinal Dehydroascorbic Acid (DHA) Transport Mediated by the Facilitative Sugar Transporters, GLUT2 and GLUT8. J Biol Chem. 2013; 288(13): 9092–9101.

133. Jin-Sik Bae, Tae-Hyun Kim, Mi-Young Kim, Joo-Man Park, and Yong-Ho Ahn. Transcriptional Regulation of Glucose Sensors in Pancreatic β-Cells and Liver: An Update.Sensors (Basel). 2010; 10(5): 5031–5053.

万方数据

附　录

专业词汇英汉对照表

英文缩写	英文全称	中文
AMPK	AMP-activated protein kinase	AMP 蛋白激酶
ARC	Arcuate nucleus	弓形核
C-P	C-Peptide	C 肽
CEE	Conjugated equine estrogen	共轭马雌激素
E1	Estrone	雌酮
E2 or E	17-β estradiol	17-β 雌二醇
E3	Estriol	雌三醇
E4	Estetrol	雌四醇
ER	Estrogen receptor	雌激素受体
ERE	Estrogen response element	雌激素反应元件
EST	Estrogen sulfotransferase	雌激素磺基转移酶
GLUT2	Glucose transporter type2	葡萄糖转运蛋白 2
GLUT4	Glucose transporter type4	葡萄糖转运蛋白 4
Gck	Glucokinase	葡萄糖激酶
G-6-P	Glucose 6-phosphatease	葡萄糖-6 -磷酸酶
GLP-1	Glucagon-like peptide-1	胰高血糖素样肽-1
GLP-1R	GLP-1 receptor	GLP-1 受体
GPER	G protein-coupled ER	G 蛋白耦联 ER
HFD	High fat diet	高脂肪饮食
HGP	Hepatic glucose production	肝葡萄糖生产
HRT	Hormone replacement therapy	激素替代疗法
INS	Insulin	胰岛素
IR	Insulin Receptor	胰岛素受体
LDL	Low-density lipoprotein	低密度脂蛋白
Leprb	Leptin receptor	瘦素受体
LXR	Liver X receptor	肝 X 受体
NPY	Neuropeptide Y	神经肽 Y
OVX	Ovariectomy	卵巢切除
P	Progesterone	黄体酮

万方数据

PCK	Phos phoenolpyruvatecarboxykinase	磷酸烯醇式丙酮酸羧激酶
PPT	Propylpyrazole triol	丙基吡唑三醇
SERM	Selective estrogen receptor modulator	选择性雌激素受体调节剂
SREBP-1c	Sterol regulatory element-binding protein 1c	固醇调节元件结合蛋白 1c
STZ	Streptozotocin	链脲佐菌素
VMH	Ventromedial hypothalamus	腹内侧下丘脑
VMN	Ventromedial nucleus	腹内侧核
WAT	White adipose tissue	白色脂肪组织

万方数据

硕士期间科研成果

一、发表论文

1. Qiankun Liang, Lanfang Mao, Xiaojuan Du, Yunxia Li, **Yuan Yan**, Jingjing Liang, Junhong Liu, Longde Wang, Hongfang Li*. Pingwei Capsules improve gastrointestinal motility in rats with functional dyspepsia. Journal of Traditional Chinese Medicine. 2018; 38(1-8).

2. Qiankun Liang, **Yuan Yan**, Lanfang Mao, Xiaojuan Du, Jingjing Liang, Junhong Liu, Longde Wang, Hongfang Li*. Evaluation of a modified rat model for functional dyspepsia. Saudi Journal of Gastroenterology. 2018 in press; doi: 10. 4103/sjg. SJG_505_17.

3. Yunxia Li, Jinbing Huang, **Yuan Yan**, Jingjing Liang, QianKun Liang, Yanyu Lu, Zhifeng Tian, Hongfang Li. Preventative effects of resveratrol and estradiol on streptozotocin-induced diabetes in ovariectomized mice and the related mechanisms. Plos one. under review.

4. Xiao-Yan Pang, Yun-Xia Li, Yuan Gong, **Yuan Yan** , Hong-Fang Li, Ying Zhu. Sesquiterpenes from the whole plants of Parasenecio roborowskii. FITOTERAPIA. 2017; 116: (24-33).

5. Dong-Dong Xu, **Yuan Yan**, Chun-Xiao Jiang, Jing-Jing Liang, Li Hongfang, Quan-Xiang Wu, Ying Zhu. Sesquiterpenes and diterpenes with cytotoxic activities from the aerial parts of Carpesium humile . FITOTERAPIA. 2018; 128: (50-56).   .

6. **剡 媛**, 梁晶晶, 赵 丽, 岳建云, 鲁 彦，李红芳. 黄体酮对 STZ 所致糖尿病发生的影响及机制研究. 中国糖尿病杂志, 审稿中

7. 参编专著《胃肠动力学基础与中西医临床》. 主编 李红芳 兰州大学出版社.

二、参与科研项目

1. 国家自然科学基金

（1）以第 6 完成人参与《基于脑肠轴探讨肝郁脾虚症功能性消化不良多肽组学及疏肝健脾法干预的研究》项目 编号：81360540

（2）以第 6 完成人参与项目《几种青藏高原藏药植物中抗癌新倍半萜的发现与机制研究》 项目编号：21272103

（3）以第 6 完成人参与项目《基于脑肠轴对肝郁脾虚型肠易综合症（IBS）肠道微生态及水液代谢组学的研究》项目 编号：8166150142

2. 以第 2 完成人参与甘肃省中药管理局项目：《含白黎芦醇中药制剂对 2 型糖尿病胰岛素抵抗的干预研究》项目 编号：GZK-2015-23

三、参加学术会议

1. 王炳和《Carbon Monoxide as a Terapeutic Agent》 2017-6-6 逸夫科学馆报告厅
2. 于金明《精准肿瘤学新实践 》 2017-6-22 本部学生活动中心学术报告厅
3.《如何实现损伤组织的修复和完美再生》2017-7-14 逸夫科学馆报告厅
4. 林建生《Brain Structures and Mechanisms Responsible for Cortical Activation and Wakefulness 》 2017-10-27 逸夫科学馆报告厅
5.《甘肃省生理科学会第十四届代表大会暨学术交流的大会》 甘肃省生理科学会 主办 2016-12-3 逸夫科学馆报告厅

万方数据

致　谢

时光荏苒,我的硕士生涯已接进尾声。这几年的时光既漫长又短暂,其中充满了酸甜苦辣,更有收获和成长。几年来，感谢陪我一起度过美好时光的每位尊敬的老师和亲爱的同学，正是你们的帮助，我才能克服困难，正是你们的指导，我才能解决疑惑，顺利完成学业。

本人的学位论文是在我的导师李红芳教授的殷切关怀和耐心指导下完成的,衷心感谢我的导师对我的悉心指导和关怀。从课题的选择、项目的实施，直至论文的最终完成，李老师都始终给予我耐心的指导和支持，我取得的每一点成绩都凝聚着恩师的汗水和心血。恩师开阔的视野、严谨的治学态度、精益求精的工作作风，深深地感染和激励着我，在此谨向我的导师致以衷心的感谢和崇高的敬意！

感谢兰州市解放军第一人民医院检验科主任鲁彦和岳建云医生在血清雌二醇和黄体酮测定实验中给予的支持和帮助!

感谢方泉教授和张楠老师及生理所其他老师的的帮助和关心！

感谢梁乾坤师兄，李云霞、杜晓娟和毛兰芳师姐以及梁晶晶和赵丽师妹给予我的关心和帮助!

感谢邱小青和卢研宇老师的支持和帮助,感谢辅导员李森和教秘张元元老师的关心和付出！

万方数据
